# Ultrafast Nonlinear Vibrational Spectroscopy of Aqueous Solutions at Surfaces and in Heterogeneous Environments

**DOI:** 10.1021/acs.chemrev.6c00516

**Published:** 2026-10-27

**Authors:** Arsh S. Hazrah, Johannes Hunger, Yair Litman, Benjamin P. Fingerhut, Minhaeng Cho, Mischa Bonn

**Affiliations:** 1 28308 Max Planck Institute for Polymer Research, Ackermannweg 10, 55128 Mainz, Germany; 2 Department of Chemistry and Center for NanoScience, 9183 Ludwig-Maximilians-Universität München, Butenandtstr. 5-13, 81377 München, Germany; 3 Center for Molecular Spectroscopy and Dynamics, Institute for Basic Science (IBS), Seoul 02841, Republic of Korea.; 4 Department of Chemistry, Korea University, Seoul 02841, Republic of Korea

## Abstract

Water and aqueous electrolytes at surfaces, interfaces, and in confinement behave differently from the bulk, yet linear spectroscopy rarely combines the chemical specificity, surface selectivity, and temporal resolution needed to resolve electric double-layer formation, specific-ion adsorption, and hydration at interfaces and under nanoconfinement. This review assesses how ultrafast nonlinear vibrational spectroscopy, comprising two-dimensional infrared spectroscopy, vibrational sum-frequency generation (SFG), and time-resolved SFG, probes the hydrogen-bond network and the dynamics of interfacial water and electrolytes on femtosecond-to-picosecond timescales. We first establish vibrational benchmarks for bulk water and simple electrolytes, then treat soft aqueous boundaries, including air–water, hydrophobic and hydrophilic, lipid, surfactant, and nucleic acid interfaces. We next examine solid–liquid and electrochemical systems, spanning metal oxides, minerals, and field-induced effects, before turning to water and proton dynamics in nanoconfined geometries and complex architectures. Throughout, experimental results are set against theoretical and computational frameworks, from polarizable force fields to machine-learning potentials. Our aim is to identify which molecular-level features of interfacial water are now firmly established, where the prevailing mean-field description of the electric double layer breaks down, and which experiments would resolve the outstanding discrepancies.

## Graphical abstract

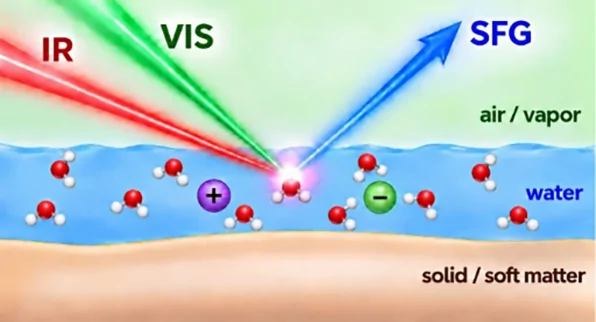

## 1 Introduction

Water is unusual among liquids because every molecule can both donate and accept two hydrogen bonds. The liquid is therefore best described not as a collection of independent molecules but as a fluctuating, three-dimensional hydrogen-bond network.^1,2^ This network is directional and cooperative enough to impose local tetrahedral order, yet weak enough that individual bonds break and reform on a picosecond time scale.^3–5^ It is precisely this combination of structure and lability that underlies water’s anomalous thermodynamic behavior,^6^ its high dielectric constant, and its extraordinary capacity to solvate ions^7^ and to support proton transport.^8,9^ Dissolved ions perturb the network in ways that reach well beyond simple continuum electrostatics: an ion replaces water–water hydrogen bonds with water–ion interactions whose strength, directionality, and polarizability are set by its charge density, thereby reshaping both the local hydrogen-bond topology and the collective dynamics of the surrounding molecules.^7,10^ Small, strongly hydrated ions slow the reorientation of water in their first solvation shell, whereas large, weakly hydrated ions leave it largely intact.^10^ How far this perturbation extends, and whether single-ion effects are additive at all, remains contested: early femtosecond studies found essentially no influence beyond the first shell for any ion,^11^ whereas specific cation–anion combinations were later shown to slow water cooperatively over several solvation shells, an effect that cannot be recovered by treating the two ions independently.^12^ At the high concentrations relevant to batteries and biological compartments, the very distinction between “bulk” and “hydration” water dissolves.^13,14^ Interfaces impose a second, complementary perturbation. They truncate the hydrogen-bond network, break translational symmetry, and generate strong, spatially varying electric fields over nanometer length scales.^15,16^ The central theme of this Review is what happens when these two perturbations act together, how the hydrogen-bond network reorganizes, structurally and dynamically, when water is simultaneously bounded by an interface and populated with ions, and how nonlinear vibrational spectroscopy allows us to observe that reorganization with molecular and femtosecond resolution.^17,18^ Answering these questions is relevant for a wide range of systems: aqueous electrolytes are found in a wide variety of natural and technological environments, and are fundamental to charge transport and chemical reactions across the atmosphere, oceans, mineral surfaces, batteries, catalysts, membranes, aerosols, and biological cells (Figure 1).^19–21^ Even when considering bulk solutions, ions that may appear simple at first glance can reorganize the hydrogen-bond network of water, reshape the local solvation environment, alter collective molecular fluctuations, and influence key properties such as transport, acidity/basicity, ion conductivity, and intermolecular interactions.^7,22^

**Figure 1:**
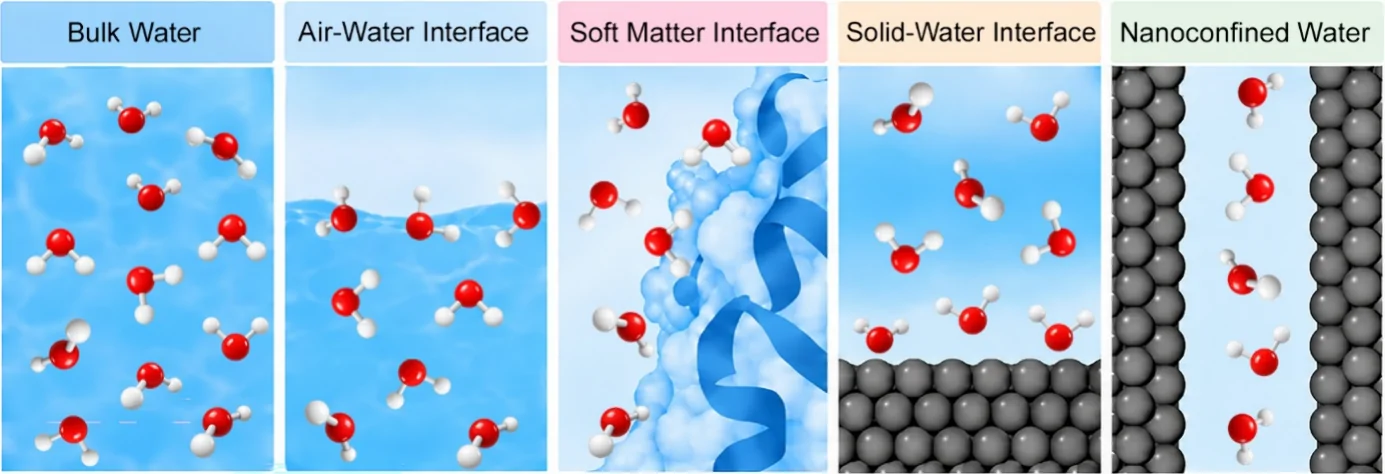
Schematic representation of water in bulk, at the air–water interface, in contact with soft (biomolecular) interfaces, at a solid–liquid interface, and under nanoscale confinement. These environments impose distinct structural constraints on the hydrogen-bond network, ranging from bulk-like three-dimensional organization to interfacial orientational ordering and confinement-induced perturbations.

In more complex settings, such as at interfaces or within heterogeneous environments, these effects become more pronounced. The solvent environment is no longer uniform or isotropic. Instead, factors such as molecular packing, spatial variations of the dielectric response, solution composition, and electrostatic potential can vary over nanometer or even subnanometer length scales. This gives rise to highly localized and structurally diverse regions, further enhancing the complexity of the aqueous electrolyte behavior.

Aqueous systems are characterized not only by spatial heterogeneity at interfaces and within heterogeneous environments but also by extraordinary diversity of dynamic behaviors. These dynamics unfold across a broad spectrum of time scales, each contributing uniquely to the overall properties of the system. At the fastest end, inertial motions on the order of tens of femtoseconds encompass the vibrational movements of water molecules. These rapid movements set the stage for subsequent slower processes, such as hindered rotational and translational motions, which occur over slightly longer intervals. One of the most important aspects of aqueous dynamics is the continual making and breaking of hydrogen bonds, which typically take place over a span of a few picoseconds,^3,23–27^ illustrating the ongoing reorganization within the local hydrogen bond network. Such rearrangements are essential for maintaining the structural flexibility that gives rise to many of the unique properties of water and its aqueous solutions. Over even longer durations, diffusion processes become prominent. These involve the transport of ions and extend from tens of picoseconds to nanoseconds. The interaction between rapid molecular motions and slower, collective changes in hydrogen bonding networks is particularly significant in determining the behavior of aqueous solutions, especially at interfaces and within confined spaces.

Therefore, the motivation for this Review centers on a fundamental challenge in recent solution and interfacial science. More specifically, our current understanding of how water and dissolved ions behave when the translational symmetry is disrupted will be reviewed. Such a disruption can occur due to the presence of a surface, the application of an external field, or spatial confinement. Under these conditions, the uniform properties typical of the bulk liquid no longer hold, leading to profound changes in the system’s physical and chemical characteristics. A central objective is therefore to determine how to characterize these behaviors at the molecular level, especially on timescales relevant to structural relaxation, molecular and ion transport, and chemical transformations. This question is of significant importance because a wide range of technologically and chemically critical processes take place under heterogeneous, complex conditions rather than in homogeneous, isotropic bulk liquids. Thus, gaining insights into these interfacial and confined environments is essential for advancing our understanding of numerous practical and fundamental phenomena.

To gain insight into the behavior of aqueous solutions, it is helpful to begin with classical theoretical frameworks that have shaped our understanding of these systems. One of the foundational models is Debye–Hückel (DH) theory,^28^ which systematically describes electrostatic screening in dilute electrolyte solutions. According to DH theory, the presence of solvent or ions in solution reduces the effective electrostatic interactions between charged species, as their interactions are screened or modulated. Such a screening effect is central to explaining why ionic interactions in solution are often much weaker than in a vacuum, and the theory has provided a quantitative approach to predicting how ions behave in dilute conditions.

In addition to DH theory, the Gouy–Chapman–Stern (GCS) model^29,30^ provides a more detailed perspective on the structure of the electric double layer (EDL) that forms at charged interfaces. The GCS model begins by distinguishing between two key regions: the diffuse layer and the Stern layer. The diffuse layer is where ions are distributed in a gradient resulting from a balance of thermal motion and electrostatic attraction to the charged wall. The Stern layer is where ions accumulate more closely to the surface in a relatively compact region. The Stern layer typically extends about 2–5 Å from the interface and is characterized by a higher concentration of specifically adsorbed ions. By assuming the existence of these two regions, the GCS model clarifies how charge is compensated at interfaces and how localized and diffuse ionic distributions together shape the properties of aqueous interfaces.

As the salt concentration increases, ions tend to form clusters. So, further concepts such as ion pairing,^7^ solvent-shared and contact ion configurations, ion cluster formation, and the distinction between inner- and outer-sphere adsorption have been developed, and they provide a descriptive language for characterizing how ions approach surfaces and interact with one another in water, still in the context of *comparatively* dilute salt solutions. These frameworks collectively provide a basis for describing the spatial arrangement and screened interactions of ions as they move toward surfaces and one another, setting the stage for a deeper discussion of the aqueous solution behavior.

In highly concentrated salt solutions,^13,31^ the solution structure departs significantly from dilute systems and the hydrogen-bonding network of water is largely disrupted.^31,32^ In these systems, ions form interconnected networks, where the classical Debye–Hückel (DH) framework is no longer applicable. A comprehensive liquid theory that accurately describes the behavior of such concentrated aqueous electrolytes has yet to be developed, leaving the field with significant theoretical challenges in capturing the intricacies of these systems.

At interfaces, aqueous solutions often exceed the limits of the continuum mean-field descriptions. These models treat water as a spatially averaged dielectric medium and ions as point charges, whereas interfacial water shows distance-dependent changes in orientation, hydrogen bonding, dynamics, and ion solvation structure. Ions have finite size, hydration shells, polarizability, and chemical specificity, while water actively participates in electrostatic screening, ion solvation, proton transfer, and interfacial reorganization.^12,33^ At phase boundaries, broken symmetry, dielectric discontinuities, and locally modified intermolecular interactions, further drive the breakdown of mean-field approximations.^34^

Given the spatial and temporal heterogeneities in complex environments, it is useful to identify recurring conceptual themes that can provide a unifying framework across different soft interfaces. One such theme is how hydrophobic and hydrophilic hydration are encoded in molecular-level spectroscopic signatures. These interfaces include air–water, oil–water, and water-biomolecule boundaries.^35–37^ In addition, solid interfaces such as those formed by oxides, minerals, metals, and two-dimensional materials present their own distinct hydration behaviors. Understanding these differences at the molecular level is essential for characterizing the role of hydration in complex aqueous systems and ultimately, their functions. Another key focus involves elucidating how specific ions adsorb to interfaces. This includes examining the extent to which ions retain their hydration shells upon adsorption and investigating the impact of ion adsorption on the structure and dynamics of interfacial water. Furthermore, it is important to explore how adsorbed ions compensate the charge of interfaces and the organization of the electric double layer. These questions are vital to a comprehensive understanding of the behavior of ions and water at complex interfaces like battery electrodes.

In the study of aqueous interfaces, it is also important to clarify the distinction between the physical and chemical changes occurring in these systems. On the one hand, there are primarily physical transformations. These include wetting transitions, in which the extent of water coverage on the surface changes; phase changes, such as melting or crystallization; conformational shifts of molecules or assemblies at the interface; and dielectric reorganization, in which the distribution of charges and dipoles adapts to the local environment. On the other hand, truly chemical changes are also possible at the interfaces. These processes involve the making and breaking of chemical bonds and may include proton transfer, ligand exchange, chemical adsorption of reactive intermediates onto surfaces, charge transfer between species, and formation or cleavage of chemical bonds. Distinguishing between these physical and chemical phenomena is essential for understanding the mechanisms underlying the interfacial reactivity. In fact, this distinction is especially significant at electrochemical interfaces, where interfacial electric fields can profoundly influence the behavior and reactivity of the molecules. Such fields can bias molecular orientation at the surface, shift the vibrational frequencies of interfacial species under spectroscopic investigation, alter the symmetry of hydration shells, and redirect the energy flow along reaction pathways. As a result, the interplay between physical and chemical changes at these interfaces determines the unique reactivity and selectivity observed in the electrochemical systems.

A key question in aqueous interfacial science is whether bulk water benchmarks can predict interfacial behavior. The challenge lies in assessing the applicability of bulk-derived parameters to interfaces, where emergent properties may differ substantially from bulk behavior. Soft interfaces, such as those found between liquids or at biomolecular (proteins, membranes, nucleic acids, etc.) or polymer boundaries, can exhibit markedly different characteristics from atomically structured solid interfaces. In solid–liquid systems, factors like well-defined surface sites, the presence of defects, and diverse charging mechanisms introduce pronounced spatial heterogeneity, which can fundamentally alter interfacial behavior compared to that observed in more uniform or ‘soft’ environments. Additionally, when water and ions are confined within nanoscale or even Ångström-scale volumes, a new set of questions emerges. Confinement can profoundly alter the properties of water itself: its dielectric constant,^38,39^ melting temperature,^40^ and phase diagram,^41^ can all shift substantially, with direct consequences for ion solvation,^42,43^ ion pairing,^44^ and transport.^44^ The description of electrostatic interactions becomes substantially more challenging as well: the dielectric response of the confined medium can be highly anisotropic^45,46^ and spatially heterogeneous, going beyond continuum descriptions that assume uniform permittivity. Furthermore, the absence of a bulk-like region prevents the formation of a well-defined diffuse layer, limiting the applicability of concepts such as the electric double layer.^42^ Under such extreme confinement, do the established principles governing screening, hydration, proton activity, and transport continue to apply, or do these processes follow fundamentally different rules and exhibit extraordinary behaviors? Addressing these issues is crucial for building a comprehensive picture of aqueous systems across a range of heterogeneous environments.

All these deviations from simple mean-field descriptions require a molecular-level understanding of liquid structure, including the positions, orientations, and hydration environments of ions and water molecules, as well as the persistence of these structures and their susceptibility to perturbations. As these properties are inherently linked to molecular dynamics, resolving aqueous interfaces requires methods that can probe both static structure and time-dependent fluctuations. Vibrational spectroscopy is particularly well suited for this task because infrared (IR), Raman, and nonlinear vibrational spectroscopies report on hydrogen-bond strength, ion coordination, solvation structure, molecular orientation, and chemical speciation. These considerations motivate the structure of this Review, which progresses from bulk reference systems to soft interfaces, then to solid–liquid interfaces, and finally to nanoconfined architectures. This organization allows similarities and differences to be compared as the environment changes, providing a framework for assessing the transferability of molecular-level insights across diverse interfacial contexts. It is important to keep in mind that answering these questions involves considering both structure and dynamics. Static spectral signatures, e.g., IR absorption and Raman scattering spectra, provide invaluable information about average hydrogen-bond strength, ion coordination, solvation structure, and the chemical species at phase boundaries. However, many crucial properties are dynamic, including spectral diffusion,^47–54^ chemical exchange,^55–58^ orientational relaxation,^59^ vibrational population decay,^60,61^ energy transfer,^62,63^ and proton or ion transport.^64,65^ More importantly, how water molecules reorganize within the electric double-layer requires understanding in the time domain. In complex systems, a broad spectral feature may represent a mix of several subensembles, each differing in local hydration structure, solvation energy, orientation, and concentration, as well as their unique environments and fluctuation timescales.

Therefore, a comprehensive description of aqueous electrolytes at interfaces demands techniques that can connect the local structure to time-dependent behavior with high molecular selectivity. This requirement is especially important when one seeks to separate interfacial water from bulk-like water or solutes, distinguish the subnanometer Stern layer response from the diffuse-layer response, isolate adsorbed reaction intermediates from solvated spectators, or compare interfacial water to core water under confinement. In this regard, ultrafast nonlinear vibrational spectroscopy is exceptionally well-suited for this task because vibrational modes are direct reporters of local bonding, charge distribution, electrostatics, and hydration.^66–68^ Nonlinear signal generation provides a level of selectivity not accessible in linear spectroscopy. In practice, sequences of femtosecond laser pulses spanning the THz-to-visible range are used to coherently excite vibrational transitions and track their temporal and spectral evolution in real-time during ongoing physical or chemical transformations.

Formally, the interaction of these pulse sequences with the sample induces a macroscopic polarization *P*(*t*), which can be expanded in a perturbation series of the applied electric field *E*(*t*):^69,70^

1 $$P\left(t\right)=P^{\left(1\right)}\left(t\right)+P^{\left(2\right)}\left(t\right)+...+P^{\left(n\right)}\left(t\right)+...$$

Linear spectroscopy arises from the first-order response, whereas higher-order terms give rise to nonlinear optical signals. In interfacial systems, this hierarchy is particularly important: second-order responses provide intrinsic sensitivity to symmetry-broken environments, while higher-order contributions can capture bulk-mediated effects. The nonlinear response function encodes the microscopic dynamics of the interfacial molecular system, including vibrational eigenstates, dephasing processes, anharmonic couplings, energy-transfer pathways, and the evolution of the energy landscape under structural fluctuations, thereby linking the molecular structure to experimentally observed spectra.

The symmetry properties of the nonlinear response tensor dictate the selection rules for observed spectroscopy. By controlling the polarization, time delays, and frequencies of the input fields and by experimentally isolating specific laboratory-frame tensor elements, one can probe different phenomena. For instance, conventional sum-frequency generation (SFG) is a second-order process governed by a response tensor that inherently vanishes in centrosymmetric bulk media within the electric dipole approximation but survives at interfaces because of the broken symmetry.^71^ Two-dimensional infrared (2D-IR) spectroscopy relies on the third-order polarization, whose response tensor encodes bulk vibrational frequency correlations and population dynamics.^70,72–74^ Extending this to interfaces, 2D-SFG,^75^ first demonstrated on a molecular monolayer by Bredenbeck et al.^76^ and placed on a theoretical footing by Liang et al.^77^ and Ishiyama et al.,^78^ isolates the fourth- or even higher-order responses,^79^ combining the true surface specificity of even-order tensor elements with the dynamic correlation capabilities of multidimensional spectroscopy. The complementary information provided by the IR and SFG spectra of coupled systems is illustrated by simulations of a mechanosensitive channel.^80^

In this Review, we focus on those ultrafast nonlinear vibrational methods that (i) are inherently interface-specific or can be made selectively sensitive to interfacial layers and (ii) provide temporally resolved spectral information. These include vibrational SFG in both reflection and scattering geometries (SFS), as well as heterodyne-detected SFG (HD-SFG),^17,81–84^ which is a phase-sensitive probe of interfacial structure, 2D-IR spectroscopy in bulk and confined geometries, and 2D vibrational sum-frequency generation (2D-VSFG) at interfaces.

In the remainder of this Review, we build on this methodological foundation to discuss how these techniques have transformed our understanding of aqueous electrolytes at interfaces and in heterogeneous environments, rather than on all applications of multidimensional vibrational spectroscopy to water. We first summarize fundamental benchmarks from bulk water^27^ and simple electrolyte solutions, highlighting primarily insights essential for interpreting interfacial behavior. We then turn to electrolyte surfaces and soft interfaces, followed by solid–liquid and electrochemical interfaces, where nonlinear vibrational spectroscopy directly probes electric double-layer structure, specific adsorption, charge transfer, and vibrational energy flow. Subsequent sections address nanoconfinement and complex architectures, including porous and membrane systems, in which water and ions experience intrinsically heterogeneous environments (see Figure 1). We close with an outlook that integrates theory and simulation with experiment and identifies key open questions. Topics including supercooled water, broad Hofmeister and biomolecular surveys, and nonaqueous electrolytes are considered only briefly, insofar as they provide useful contrasts for understanding aqueous interfacial electrolytes.

## 2 Fundamental Benchmarks: Bulk Water, Simple Electrolytes, and the Air–Water Interface

In this section, we provide an overview of the structural and dynamic properties of liquid water and dilute electrolyte solutions for reference. Time-resolved techniques, such as pump–probe, polarization-resolved transient absorption, and 2D-IR spectroscopy, have enabled us to obtain a close-to-quantitative picture of hydrogen-bond (H-bond) dynamics in bulk liquid water over the last two decades. Results for liquid water are compared to those for dilute electrolyte solutions, revealing differences in hydration dynamics within their solvation shells. We start by defining the fundamentals of the vibrational structure of water, i.e., the relevant intra- and intermolecular modes and their relation to H-bond structure, the broadening of vibrational bands, and intermode couplings. We then transition to the vibrational dynamics of water, focusing initially on phenomena quantified by phase evolution (the off-diagonal elements of the density matrix). The loss of phase relation encodes the time evolution of randomization of the sample, characterizing spectral diffusion and the underlying H-bond dynamics. We then proceed to population-based vibrational dynamics (the diagonal elements of the density matrix), which provides insight into vibrational lifetimes and ionization dynamics. Finally, we compare it to the canonical hydrophobic air–water interface. The section concludes with a mechanistic comparison of bulk and interfacial hydrogen bond dynamics.

### 2.1 Vibrational Structure of Water

#### 2.1.1 Vibrational Modes, Frequency Correlation, and H-Bond Strength

The intramolecular OH stretching vibration serves as the primary reporter of the local hydrogen-bonding environment in liquid water and dilute aqueous electrolytes. In the gas phase, the symmetric and asymmetric stretches of H_2_O are well separated and narrowly defined, but in the condensed phase, the corresponding OH stretching manifold is strongly red-shifted and broadened by hydrogen bonding, frequency–frequency fluctuations, and intermolecular coupling.^1,27,85^ As a result, the OH stretch frequency and line shape (Figure 2) encode local variations in hydrogen bond strength and geometry,^86^ and have therefore become the central observable for ultrafast nonlinear vibrational spectroscopy of aqueous systems.^87^ At lower frequencies, intramolecular bending, librational, bend+libration combination, intermolecular bending, and hydrogen bond stretching motions exhibit a pronounced collective character that reflects mesoscale organization of the hydrogen bond network.

**Figure 2:**
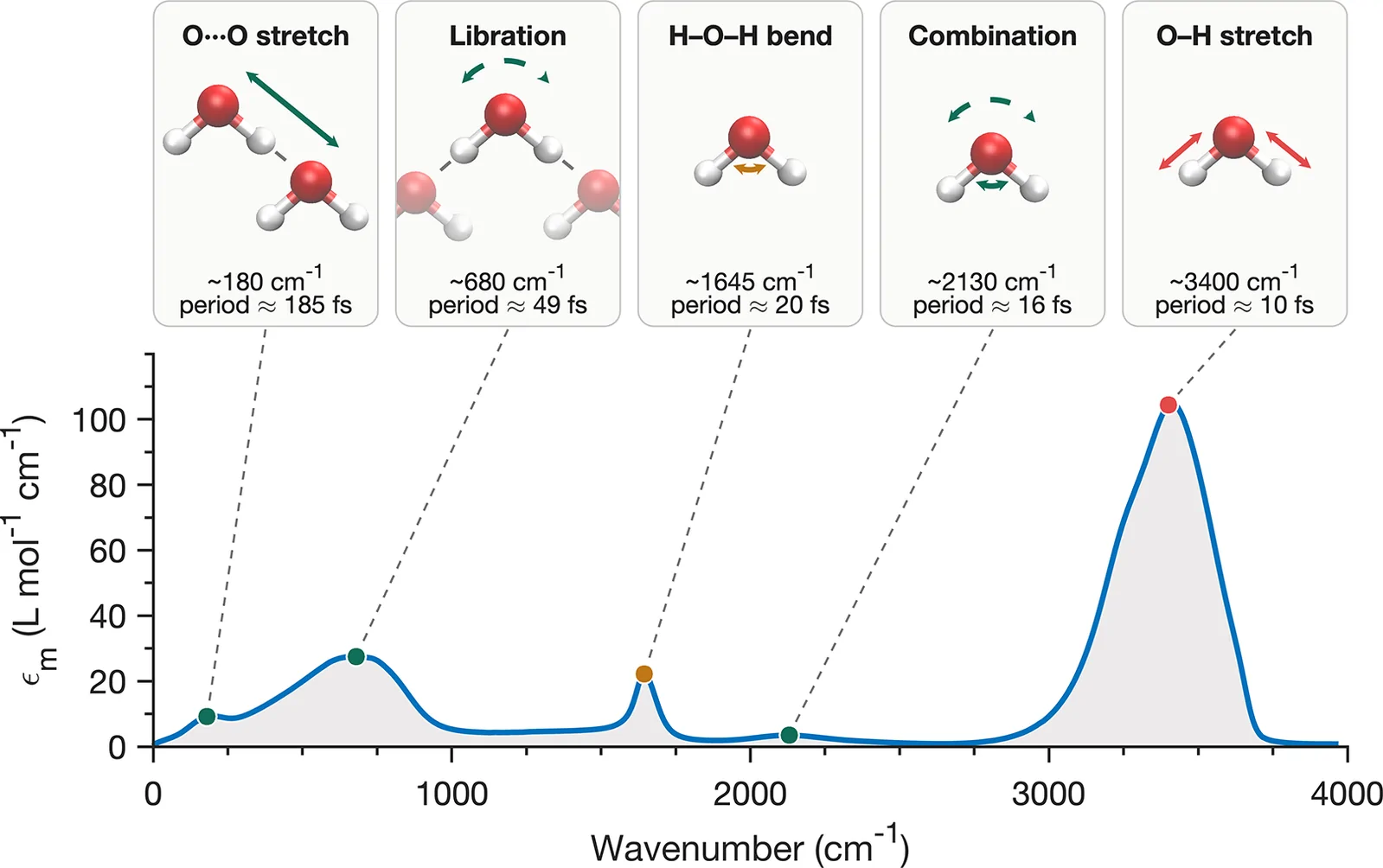
Vibrational modes of liquid water. Top: schematic of vibrational modes ordered by increasing wavenumber; approximate oscillation periods are indicated. Arrows symbolize atomic displacements. Bottom: Molar absorption coefficient (*ε*_m_) spectrum of liquid water at 25 °C, taken from Bertie and Lan on a linear scale from 0 to 4000 cm^–1^.^97^

The HOH bending vibration in liquid water is blue-shifted relative to the gas-phase value (to ν_2_ ≈ 1645 cm^–1^ in the liquid, Figure 2), reflecting the modified intra- and intermolecular force constants in the condensed-phase hydrogen-bond network. In recent years, this mode has experienced a renaissance as a structural and dynamical probe,^88–92^ because it is unique to water and absent in other solutes with OH-groups. As such, it has been particularly employed in interfacial studies, where its frequency position, line width, and coupling to other low-frequency modes have been shown to encode local hydrogen-bond geometry and connectivity more transparently than the OH stretching band. In contrast, interpretation of the stretching continuum can be complicated by strong anharmonic couplings and Fermi resonances with the overtone of the bend (cf. Section 2.1.3), making the bending vibration an increasingly attractive complementary observable for resolving interfacial water structure.

The water bend+libration combination band, centered near 2130 cm^–1^, has emerged as a useful intrinsic and collective reporter of the hydrogen-bonding network in liquid water. Unlike site-specific probes such as the OD stretch of dilute HOD, this band arises from the intermolecular anharmonic coupling between the molecular HOH bending mode and collective librational motions. Consequently, its frequency directly reflects the overall rigidity and strength of the aqueous H-bonded network.^93^ Comprehensive IR spectroscopy studies have demonstrated that this band is highly sensitive to diverse solutes, showing distinct responses to Hofmeister ions: chaotropic ions induce a red-shift indicative of a weakened network, while kosmotropic ions typically cause a blue-shift or negligible change.^93^ Beyond simple electrolytes, the combination band reveals unique ways in which denaturants and osmolytes modulate their environment. The band also shows exceptional sensitivity to hydration changes in crowded or confined environments.

At lower frequencies extending into the far-infrared region, prominent intermolecular vibrations are identified, commonly attributed to a broad librational band (500–1000 cm^–1^) arising from the hindered rotations of water molecules in the H-bond network and intermolecular H-bond stretching vibrations (∼180 cm^–1^). These modes are not efficiently probed by mid-IR spectroscopy but are naturally accessed by terahertz,^94^ low-frequency Raman,^95^ or Kerr techniques,^96^ which provide complementary information on collective water dynamics and their modification by ions, interfaces, and confinement.

#### 2.1.2 Broadening Mechanisms

The broad OH stretching band of liquid water, centered around 3400 cm^–1^, spans > 400 cm^–1^ (full width at half maximum) and reflects the distribution of instantaneous H-bonds and geometries sampled by thermal fluctuations. Such an interpretation suggests that the dominant broadening mechanism of the OH stretching band is from the structural inhomogeneities in the liquid rather than fast motional narrowing. Disentangling the two contributions is a central question addressed by nonlinear vibrational spectroscopy of water and the strong motivation for 2D-IR spectroscopy. Frequency-frequency correlation functions quantifying the time-dependent randomization of the OH stretching frequencies (i.e., structure) and extracted from the spectral diffusion dynamics of 2D-IR spectra provide a quantitative characterization of the broadening mechanism (cf. Section 2.2).

The interpretation of structural inhomogeneities as a dominant contribution to the width of the OH stretching band is further supported by extensive simulations of its spectral envelope. The electrostatic field frequency maps^66,86,98^ account accurately for a large fraction of the line width and shape, and more refined polarizable models^99,100^ provide an almost quantitative description of the line shape, explaining the asymmetry of the band due to the underlying structural H-bond distribution. Notably, only a small number of hybrid-GGA (revPBE0-D3) and meta-GGA functionals (PW6B95-D3 with and r2SCAN without exact Hartree–Fock exchange) provide an accurate description of the vibrational spectrum of liquid water in first-principles density functional simulations^101–103^ that are comparable to the quality of state-of-the-art models^100,104^ and machine-learning (ML) approaches.^105–110^

#### 2.1.3 Mode Couplings and Mode Delocalization

The vibrational spectrum of liquid water is affected by a multitude of coupling mechanisms that operate at different length scales, thereby determining properties such as line shape and relaxation dynamics, e.g., in 2D-IR experiments. At the intramolecular level, a Fermi resonance between the fundamental of the OH stretching vibration (ν_3_ ∼ 3400 cm^–1^) and the first overtone of the HOH bending vibration (2ν_2_ ∼ 3290 cm^–1^)^113^ is consequential in defining the line shape (note the characteristic low-frequency shoulder ∼ 3200 cm^–1^ in the IR spectrum of Figure 2)^114^ and energy relaxation dynamics^60,61^ (cf. Section 2.3.1). A most direct measurement of the stretch–bend Fermi resonance is the observation of characteristic, broad excited-state absorption (ESA; 1500–1900 cm^–1^) upon HOH bend excitation in broadband 2D-IR spectra,^64^ corresponding to a transition from the bend excited state to a state with strong mixed stretch–bend and excitonic character.

At the intermolecular level, dipole–dipole coupling between the OH stretching vibrations of water molecules^62^ creates fluxional vibrational excitons,^63^ delocalized across the stretching vibrations of multiple H_2_O molecules. The OH stretching band of liquid water is thus best viewed as collective excitonic vibrations that, due to thermal structural fluctuations, constantly reorganize on a ∼ 80 fs time scale.^115,116^ Such complications can be mitigated by using isotopically diluted samples (HOD in H_2_O) with a local OD vibrational probe. Compared to the coupling of OH stretching vibrations, the intermolecular coupling of bending vibrations is substantially weaker^90^ due to the much smaller transition dipole moment of the bend vibration. The observation of the rapid randomization of the HOH bend orientation was attributed to relatively strong bend-librational coupling, a pathway suggested by Hynes and co-workers^117^ as dominant for excess-energy dissipation and equilibration of liquid water (see Section 2.3.1).

#### 2.1.4 Isotope Exchange and Nuclear Quantum Effects

Most molecular dynamics (MD) simulations assume a classical description of the nuclei. Nevertheless, the quantitative interpretation of the liquid water vibrational spectroscopy requires accounting for nuclear quantum effects (NQE). Evidently, the neglect of NQE for H_2_O and D_2_O will yield (besides the mass effect) the same equilibrium properties (like solvation structures), but in reality, properties differ due to the existence of NQE. An intriguing example in the context of interfacial water is the structure of H_2_O, D_2_O, and HDO at the water-vapor interface,^118,119^ where the breaking of the intramolecular symmetry in HDO is reflected in a preference of OD bonds being oriented toward the bulk. It is well established that a subtle balance of competing NQEs is operational at room temperature.^120,121^ NQE on the OH stretching vibration reflect two competing contributions. Intermolecular zero-point energy and proton tunneling destabilize the hydrogen-bonding network, promoting delocalization and a blue shift of the OH stretch. This is counteracted by intramolecular zero-point motion, which modifies the average water monomer geometry, increases the dipole moment, and strengthens intermolecular interactions—effectively red-shifting the OH stretch and partially offsetting the intermolecular quantum effects. The net effect of NQE is then a 10–20% increase in the frequency variance of the OH stretching band at room temperature, in line with recent findings^122^ that NQEs are relevant in defining dynamical properties. In contrast, their effect on the average structural properties of water is minor.^122^ By analyzing the excess/depletion of interfacial HOD, H_2_O, and D_2_O molecules, the near-complete cancellation of competing NQEs was recently quantified experimentally,^123^ explaining the overall moderate NQE in liquid water. Recent theoretical decomposition of the IR and anisotropic Raman spectrum of liquid water into coupled, uncoupled, and inter/intramolecular contributions showed that, besides the frequency redshift, vibrational coupling in the fundamental stretching band is largely classical,^124^ consistent with early mixed quantum-classical treatments in which the intramolecular coupling of two local OH oscillators, evaluated on classical MD configurations, captured the essential two-band structure of the interfacial SFG spectrum and its symmetric/antisymmetric mode character,^125^ in contrast to combination bands, overtones, and Fermi resonances that are truly quantum in nature.^114,126,127^

The substitution of hydrogen (H) by deuterium (D) leads to a slightly stronger (shorter) H-bond in D_2_O compared to H_2_O^128^ due to the smaller zero-point motion of D (Ubbelohde effect, see ref 129 for the anti-Ubbelohde effect, depending on the strength of the hydrogen bond). Data from correlated vibrational spectroscopy^130^ are consistent with the notion that D_2_O forms about 9% more H-bonds than H_2_O. 2D-IR spectroscopy suggests that the nuclear motions of D_2_O are qualitatively different from those of H_2_O due to a shift in the balance between intra- and intermolecular couplings.^131^

#### 2.1.5 Hydration Shell of Ions

The distinct spectroscopic signatures of ionic species in aqueous electrolyte solutions (see previous reviews on the impact on water structure,^7^ Hofmeister phenomena,^132^ and ion pairing^133^) can be employed as reporters of solvation structure and dynamics. For ionic species with molecular vibrations (e.g., pseudohalides,^134,135^ carbonyls,^136,137^ or tetrahedral ions, like perchlorate, tetrafluoroborate, sulfate,^138^ and phosphates^139^), such modes serve as built-in probes with a particularly local reporter character.^68^

Solvation of excess protons H^+^ in aqueous solution leads to the emergence of new characteristic bands, in addition to the vibrational bands of neat water, specifically a broad, unstructured, continuous IR absorption (Zundel continuum) covering the 1000–3500 cm^–1^ range and a ‘proton transfer mode’ (PTM) at 1100–1300 cm^–1^. The latter has recently been identified as a hallmark of Zundel-cation-type hydration structures (H_5_O_2_^+^)^140^ with characteristic inverse anharmonicity and ultrashort vibrational lifetime (20–40 fs, cf. Section 2.2.3). The structural lifetime of the H_5_O_2_^+^ complex was estimated to exceed 480 fs.^141–143^ The hydroxide ion (OH^–^) acts as a strong hydrogen-bond acceptor and is solvated in a hyper-coordinated [OH^–^(H_2_O)_4_] complex with a characteristic high frequency OH stretch vibration (ν ∼ 3600 cm^–1^). Similar to H^+^, OH^–^ solvation leads to the emergence of an IR continuum.^65^ See also ref 144 for a review on proton and OH^–^ solvation and transport.

Taken together, these observations show that even the seemingly simple vibrational spectrum of liquid water emerges from a subtle interplay of local hydrogen-bond geometry, collective intermolecular coupling, nuclear quantum effects, and interactions with solutes. The OH stretch, HOH bend, and low-frequency intermolecular modes each emphasize different aspects of this interplay, providing a complementary set of reporters on structure from the single-molecule to the mesoscale. Establishing this multidimensional spectroscopic “fingerprint” of bulk and weakly perturbed aqueous systems is essential, as it defines the reference against which all subsequent deviations at interfaces, in the electric double layer, and under nanoconfinement must be interpreted.

### 2.2 Vibrational Dynamics of Water: Phase Dynamics

#### 2.2.1 Spectral Diffusion and H-Bond Dynamics

Spectral diffusion is a central observable in 2D-IR and 2D-SFG spectroscopy that quantitatively characterizes the broadening mechanism behind the ∼ 400 cm^–1^ width of water’s OH stretching band. The frequency–frequency correlation function (FFCF, *C*(*t*) = ⟨δω­(*t*)­δω­(0)⟩) quantifies the time-dependent randomization of structural inhomogeneities in the H-bond network. For the OH stretch of dilute HOD in D_2_O, the FFCF exhibits an initial ∼ 50 fs decay, an oscillatory feature near 180 fs associated with underdamped intermolecular vibrations, and a subsequent ∼ 1.4 ps exponential relaxation associated with collective reorganization of the liquid.^145^ The FFCF can be extracted from the three-pulse photon-echo peak shift (3PEPS)^54^ or from the evolution of 2D-IR spectra as a function of waiting time τ_2_.^52,146^ A 2D-IR spectrum recorded at short τ_2_ typically shows some degree of elongation/ellipticity along the diagonal axis that indicates a correlation of the excitation frequency and detection frequency (Figure 3), i.e., memory of the system due to structural inhomogeneities. For increasing waiting time τ_2_, the 2D-IR line shape evolves toward a circular shape, and spectral diffusion erases the memory (loss of the phase relation between excitation and detection frequencies). Following the nodal-line slope (NLS) and center-line slope (CLS) of 2D spectra thus provides a means to extract the temporal characteristic of the FFCF.^52,146^ The FFCF is related to the line shape function, and the multitime FFCF defines the full 2D-line shape and its time evolution during waiting time in 2D spectra (cf. Section 2.2.4).^52,69,146,147^

**Figure 3:**
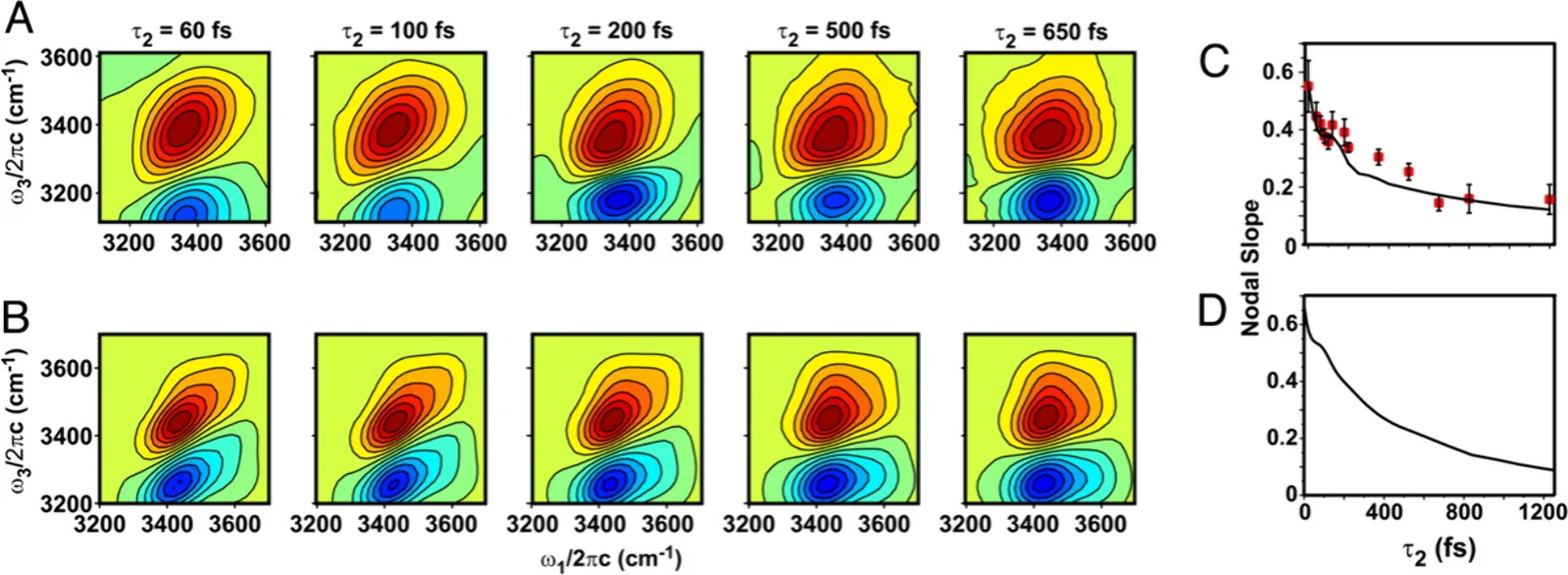
Experimental (a) and simulated 2D-IR spectra (b) of HOD in D_2_O. Both the experimental and simulated 2D-IR spectra show a reshaping from an elliptic to a round and upright line shape with waiting times τ_2_ on a 500–600 fs time scale. (c and d) Plots of the slope of the node by fitting between the fundamental and *v* = 2 ← 1 for experiment (c) and simulation (d) as a function of waiting time τ_2_. The slope of the node (squares) was calculated by fitting a straight line through the node of each surface between the limits ω_1_/2π*c* = 3250 cm^–1^ to 3450 cm^–1^. The solid line plotted in (c) is the predicted behavior based on the measured *C*(*t*) of previous data.^111^ Reproduced with permission from Eaves et al., *Proc. Natl. Acad. Sci. U.S.A.* 2005, 102, 13019–13022.^112^ Copyright 2005 National Academy of Sciences.

The overwhelming majority of studies characterizing the FFCF rely on isotopically diluted samples (e.g., HOD in D_2_O),^3,148^ measuring the spectral diffusion dynamics of local OH or OD oscillators. In neat H_2_O, energy transfer between excitonically coupled OH modes on neighboring H_2_O molecules complicates the picture. Cowan et al.^23^ demonstrated in 2D-IR experiments with a ∼ 500 nm thin film that orientational memory is lost within 50 fs in neat H_2_O (Figure 4), and a slightly slower loss of frequency correlation (176 fs) has been reported from broadband 2D-IR experiments,^116^ i.e., much faster than the H-bond exchange dynamics (∼2.5 ps).^4,149^ Librational motions on the sub-100 fs time scale together with Förster-type energy transfer^62^ among excitonically coupled H_2_O molecules^115^ establish a new structural equilibrium within ∼ 300 fs after excitation, consistent with recent structural results from ultrafast electron scattering experiments.^150^ Thus, the isotope dilution strategy provides the cleanest view for accessing the structural relaxation timescales of liquid water (Figure 3).

**Figure 4:**
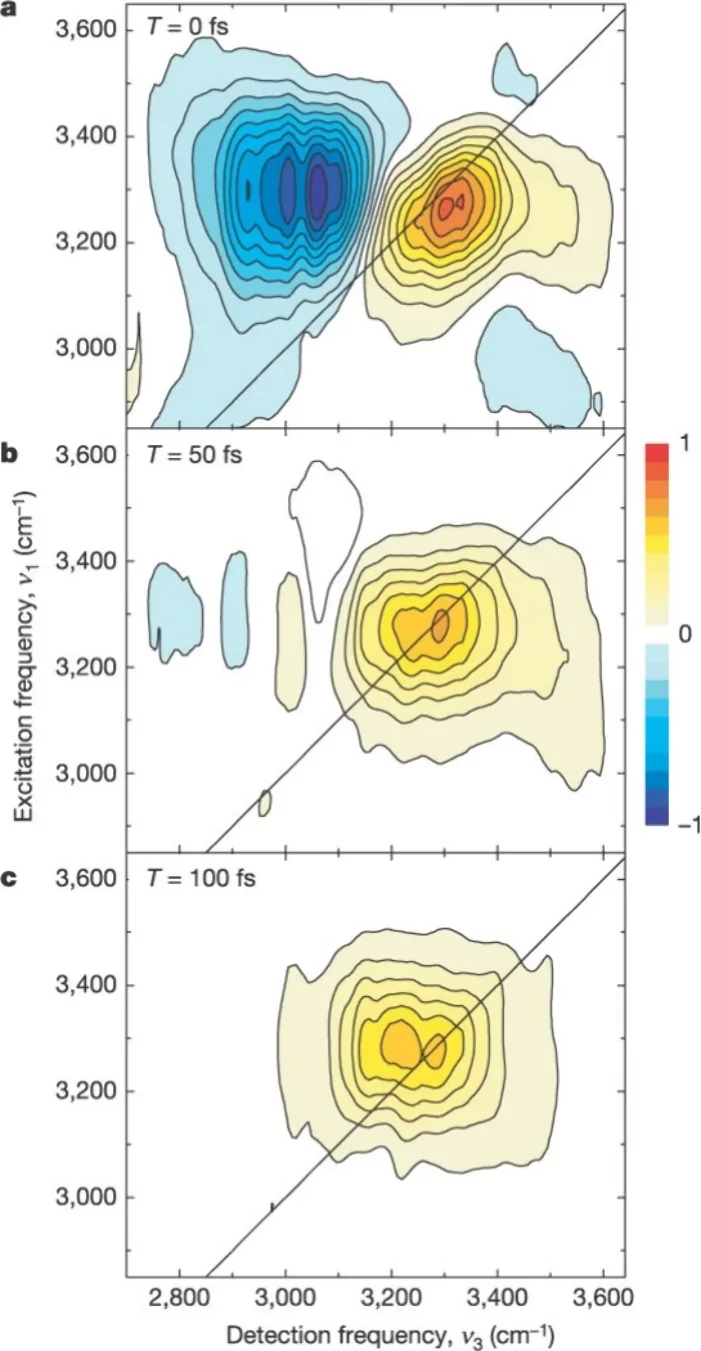
Absorptive components of the two-dimensional infrared echo spectra of pure liquid H_2_O for different population times (*T* = τ_2_). **a**, *T* = 0 fs; **b**, *T* = 50 fs; **c**, *T* = 100 fs. The inhomogeneity indicated by the stretching of the positive peak along the diagonal in the *T* = 0 spectrum has almost completely decayed by *T* = 50 fs, showing the very rapid dephasing and loss of memory in the system, clearly much faster than that revealed in the 2D-IR spectra of HOD in D_2_O (cf. Figure 3). Reproduced with permission from Cowan et al., *Nature* 2005, 434, 199–202.^23^ Copyright 2005 Springer Nature Limited.

#### 2.2.2 Cation and Anion Effects on Solvation Shell Dynamics

The sub-200-fs dynamics and ∼ 1.4 ps relaxation of the OH-stretch FFCF of dilute HOD in D_2_O provide a quantitative reference for assessing how dissolved ions or surfaces affect water dynamics.^145^ The macroscopic picture of ‘structure making or breaking’ ions (Hofmeister series, cf. Section 3.3.2) motivated extensive ultrafast vibrational spectroscopy studies to elucidate the effect of dissolved ions on the water H-bond dynamics. It was established from polarization-resolved pump–probe spectroscopy on the anisotropy of HOD in halide solutions that ion-induced retardation of the water dynamics is restricted largely to the first solvation shell^11^ and water molecules in the second solvation shell and beyond (>3–4 Å) show the same ∼ 2.5 ps anisotropy decay dynamics as bulk H_2_O. Tielrooij et al. extended these investigations to divalent cations, such as Mg^2+^, Ca^2+^, and Ba^2+^, revealing a cooperative retardation of the water dynamics for H_2_O molecules in the second hydration shell.^12^ The analysis of the CLS of 2D-IR spectra (cf. Section 2.2.4) by Gaffney and co-workers suggests that anions modulate the amplitude of the slow component τ_2_ of the FFCF but not the time scale.^151^ Recent 2D-IR experiments utilizing the spectral diffusion of the asymmetric stretching vibrations of the SO_4_^2–^ ion showed a cooperative retardation of the spectral diffusion dynamics.^138^ The slowdown of Mg^2+^/SO_4_^2–^ compared to Na^+^/SO_4_^2–^ is associated with the H-bonding structure in solvent-separated ion pairs of Mg^2+^/SO_4_^2–^. On the microscopic level, the timescales of librational motions are unaffected, but due to strong Coulomb interactions in the solvation geometries, the amplitude of the fast component is reduced. Similarly, 2D-IR experiments using the asymmetric phosphate stretching vibrations of phosphoric acid at varying pH (PO_4_^3–^, HPO_4_^2–^, H_2_PO_4_^–^, H_3_PO_4_) suggest that the protonation state is linked to the relative amplitudes of the FFCF, determining spectral diffusion dynamics around the ions.^152^

Chemical exchange dynamics of H_2_O molecules in the hydration shell of ions were monitored via cross-peaks between anion-coordinated and bulk-like OH frequencies in 2D-IR spectra, yielding a lifetime of 20–80 ps.^153^ A similar approach allowed for distinguishing and monitoring the exchange of contact ion pairs and solvent-separated ion pairs, utilizing the molecular vibrations of aqueous HNO_3_.^154^

The collective, intermolecular H-bond vibrations of aqueous electrolytes were directly addressed by 2D-Raman-THz spectroscopy,^155^ suggesting that the structure-forming properties of cations (Li^+^ > Na^+^ > K^+^) correlate with the degree of inhomogeneity in the H-bond network. Time-resolved THz-Raman spectroscopy showed that cations and anions have opposite effects on rotational-to-translational energy transfer.^156^ A direct, linear relation between macroscopic ionic conductivity and the sub-ps ion translational motion was established from linear THz spectroscopy.^157^ Addressing properties at high ion concentrations, Cho and co-workers used graph theory to show critical ion aggregation and percolation behaviors in highly concentrated salt solutions.^32^ Later, Lewis et al. identified a water-in-salt percolation threshold of ∼ 10 M (LiTFSI) above which the FFCF collapses to a single, fast librational component (∼ 0.5 ps),^158^ characterizing the dynamics of isolated water molecules in their ion cages.

#### 2.2.3 Proton and Hydroxide Dynamics

Time-resolved vibrational spectroscopy, in particular 2D-IR spectroscopy, has provided detailed insights into the hydration dynamics of excess protons in water over the past decade. The experiments addressed the extent to which solvation of excess protons by water resembles the limiting structures of the Zundel (H_5_O_2_^+^) or the Eigen (H_9_O_4_^+^) cations, i.e., if protons are better described as shared between two water molecules or localized to a single water molecule. The analysis of the stretch–bend cross-peak in broadband 2D-IR spectra (1500–4000 cm^–1^) suggested a structural lifetime > 480 fs of the Zundel complex H_5_O_2_^+^ in aqueous HCl.^141^ By isolating the proton transfer mode around ∼ 1200 cm^–1^, first in acetonitrile solvent at low H_2_O content^143^ and for HI in water,^142^ it was shown that within a given H-bond, protons undergo large-amplitude excursions, consistent with a confining low-barrier double well potential of a Zundel H_5_O_2_^+^ structure with inverse anharmonicity (Figure 5). The 2D-IR line shape of H^+^ in water is dominated by an ultrafast, sub-100-fs correlation loss between excitation and detection frequencies across the full > 200 cm^–1^ excitation window, pointing to a negligible inhomogeneous broadening. Protons in water and the Zundel species H_5_O_2_^+^ in acetonitrile display essentially the same behavior, and the PTM vibration shows strong damping due to its 20–40 fs population decay. The asymmetric low-barrier double-well picture, consistent with H_5_O_2_^+^ geometries persisting for > 300 fs, was further corroborated by broadband 2D-IR spectroscopy, which revealed cross-peaks and strong coupling between the proton-transfer mode and the bend and stretching regions of the spectrum.^159^ Using bifluoride [F···H···F]^−^, Dereka et al. subsequently demonstrated the continuous crossover from H-bonding to covalent bonding.^160^

**Figure 5:**
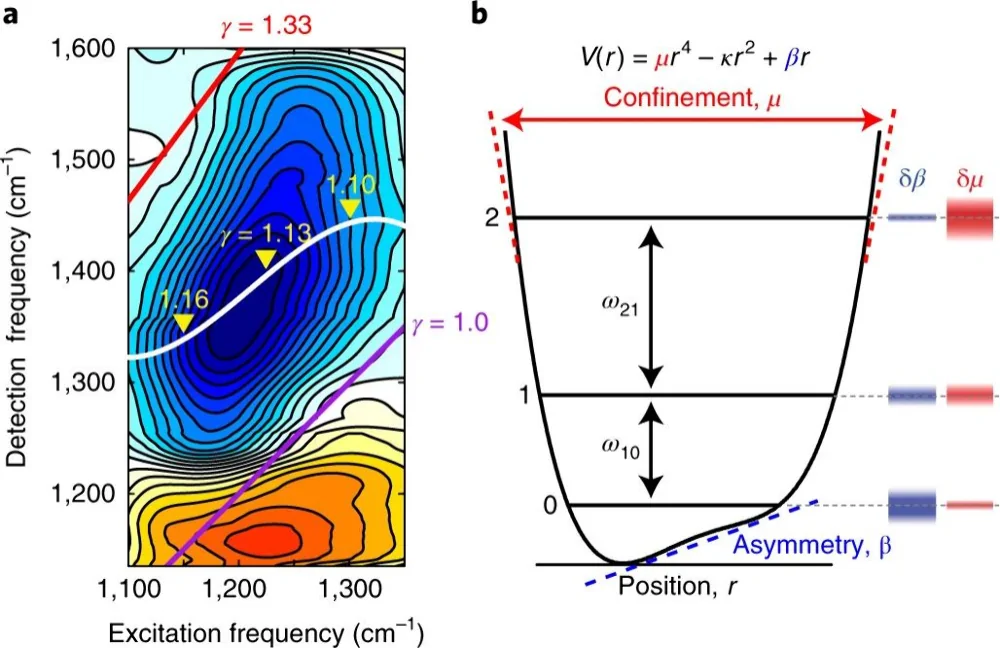
(a) 2D-IR spectrum of the diagonal region of the proton-transfer mode (PTM) measured at a waiting time τ_2_ of 150 fs, showing the peak transition frequency ratio γ = ω_21_/ω_10_ ≈1.1. The ω_21_ transition falls within a frequency range defined by the harmonic limit of γ = 1.0 and the quartic limit of γ = 1.33. The center line of the ω_21_ transition is shown in white, calculated as the signal maximum along the detection axis for each excitation frequency. Yellow arrowheads and numbers along the center line indicate the ratio γ at those points. (b) Schematic of an asymmetric quartic potential of the form *V*(*r*) = μ*r* ^4^ – κ*r* ^2^ + β*r* illustrating the qualitative effects of the proton confinement μ and asymmetry β parameters on the potential and energy levels of the 0, 1 and 2 vibrational states. The influence of changes in β and μ on the relative variance in the energy levels is illustrated by the breadth of the corresponding δβ and δμ bands. See ref 159 for a detailed analysis. Reproduced with permission from Fournier et al., *Nat. Chem.* 2018, 10, 932–937.^159^ Copyright 2018 Springer Nature Limited.

The rapid diffusion of OH^–^ through aqueous solution was addressed via 2D-IR spectroscopy,^65,161^ revealing a ∼ 110 fs time scale that is assigned to the relaxation of transiently formed configurations with a shared proton between HOD and the OD^–^ ion and a slower (∼3 ps) time scale indicative of the exchange of population between OH^–^ ions and HOD molecules due to deuteron transfer.

#### 2.2.4 Experimental Measurement and Theoretical Prediction

2D-IR spectroscopy, based on the infrared photon-echo technique, is a useful multidimensional technique capable of disentangling homogeneous from inhomogeneous broadening by tracking spectral diffusion in real time. The central quantity that dictates this loss of spectral memory is the FFCF, which describes the time-dependent fluctuations of the vibrational transition frequency induced by the dynamic solvent environment. Formally, the linear correlation function of the fluctuating transition frequency between the ground (|g⟩) and first excited (|e⟩) states is defined as:

$$C\left(t\right)=\left\langle \delta \omega_{eg}\left(t\right)\delta \omega_{eg}\left(0\right)\right\rangle .$$ 2

Within the framework of line shape theory, this correlation function fundamentally governs the nonlinear optical response through the line broadening function, *g*(*t*), which is obtained via double integration of the FFCF:

3 $$g\left(t\right)=\int_{0}^{t}d\tau_{1}\int_{0}^{\tau_{1}}d\tau_{2}C\left(\tau_{2}\right).$$

By observing how the 2D lineshapes evolve with the waiting time τ_2_, researchers can quantitatively map the decay of *C*(τ_2_) (cf. Figure 3), directly observing the timescales of solvation dynamics and hydrogen-bond network reorganizations.

To extract the FFCF quantitatively from experimental 2D spectra, analytical metrics such as the NLS and CLS are utilized.^146^ The nodal-line and related line-shape-inversion analyses were initially suggested by Kwac and Cho^52^ and subsequently used by Roberts et al.^162^ and Lazonder et al.^163^ The general response-function theory underlying the extraction of dynamics from multidimensional lineshapes has been reviewed by Abramavicius et al.^164^ In the short-time limit, 2D spectral contours are typically elongated along the diagonal, reflecting a strong memory of the initially excited inhomogeneous environments. As spectral diffusion proceeds, the contours become round and horizontally or vertically oriented when the x- and y-axes correspond to the excitation (emission) and emission (excitation) frequencies, respectively. In techniques like two-color transient dichroism (TD), transient birefringence (TB), or 2D-IR spectroscopy of a three-level vibrational system, the 2D contours exhibit distinct positive and negative peaks, and the contours between these peaks are initially slanted. The slope of the tangential nodal line separating these features, denoted as *σ*(*t*), serves as a direct, rigorous observable for the solvation dynamics. For a simple anharmonic oscillator, the nodal-line slope^52^ is essentially identical to the center-line slope. By applying short-time approximations to the generalized response functions, it can be analytically proven that this nodal-line slope is linearly proportional to the solvation correlation function, *S*(*t*).^52^ Specifically, the nodal line slope is derived as:

4 $$\sigma \left(t\right)≅\left(\frac{2}{\hbar \beta }\right)\frac{S\left(t\right)}{\Omega^{2}+w^{2}}$$

Here, β = 1/*k*_B_ *T*, Ω^2^ is the mean square fluctuation amplitude of the transition frequency, and *w* is related to the spectral bandwidth of the pump pulse. This relationship demonstrates that measuring the degree of slant *σ*(*t*) directly yields the FFCF. Complementary to the NLS, center line tracking also provides direct dynamical parameters.

To fully validate these dynamic observables, the entire 2D-IR spectrum can be reconstructed using rigorous line shape theory.^165^ For a weakly anharmonic oscillator model, incorporating the ground (|*g*⟩), first excited (|*e*⟩), and second excited (|*f*⟩) states, the total third-order polarization *P* ^(3)^(*t*) (eq.[Disp-formula eq1]) can be obtained by considering distinct density matrix transition pathways and can be expressed as the coherent sum of stimulated emission (SE), ground-state bleaching (GB), and excited-state absorption (ESA) components.^69,70^ By evaluating the distinct line broadening functions (e.g., *g*(*t*) for |*e*⟩⟨*g*| coherences and corresponding functions for |*f*⟩⟨*e*| coherences), one can simulate the destructive interference between the ESA and the respective SE and GB pathways. This bottom-up theoretical reconstruction not only accurately reproduces the waiting-time-dependent NLS, CLS, and positive-negative peak separations associated with vibrational anharmonicity but also provides an unambiguous benchmark connecting the macroscopic 2D-IR observables back to the microscopic fluctuations defined by the FFCF. Interpreting these metrics solely through a two-point FFCF commonly invokes a Gaussian frequency-fluctuation model, within which higher-order correlations are determined by the two-point function.^162^ For water, this assumption is only approximate: the OH-stretch frequency distribution is measurably skewed,^166^ coupled bending and stretching coordinates relax on dissimilar timescales,^167^ and hydrogen-bond switching is intrinsically a jump-like, non-Gaussian process.^58^ Skewed-distribution line shape theories and three-dimensional infrared spectroscopy,^168^ which access higher-order frequency correlations beyond the two-point function, highlight the need to test Gaussian assumptions in heterogeneous environments^169^ such as interfaces and concentrated electrolytes. Spectral-diffusion timescales extracted with a Gaussian model should therefore be read as model-dependent parameters rather than as complete descriptions of the underlying dynamics.

The theoretical reproduction of multidimensional vibrational spectra has advanced remarkably^63^ with the development of highly refined, flexible, and polarizable solvent models.^92,99,100,170^ By explicitly incorporating molecular polarizability and accurately mapping the fluctuating electrostatic environment to transition frequencies and transition dipole moments, these advanced frameworks successfully capture the inhomogeneous broadening, spectral diffusion, and underlying hydrogen bond dynamics. This level of quantitative agreement allows for a rigorous bottom-up reconstruction of 2D-IR line shapes, effectively bridging the gap between molecular dynamics trajectories and time-resolved vibrational observables.

Despite these impressive theoretical achievements, reproducing the entire broad water vibrational continuum remains challenging with the current potential energy surfaces. A notable discrepancy arises in highly distorted, exceptionally strong H-bonding configurations, as the parametrized electrostatics and anharmonicities fail to fully capture the extreme shifts and transition dipole enhancements characteristic of such tight local geometries. Addressing these problems requires overcoming several theoretical challenges. First, the nonreactive nature of standard classical models fundamentally prevents the description of transient bond breaking or charge delocalization, necessitating the integration of reactive force fields or machine-learning-driven reactive potentials to capture the true plasticity of the aqueous network. Second, the accurate assignment of the line shape is complicated by Fermi resonance coupling effects, such as the interplay between the stretching fundamentals and the bending overtone. Capturing these anharmonic couplings remains a critical bottleneck, requiring theoretical advances that integrate the many-body nature of interfacial water with rigorous quantum-mechanical line shape theories.

### 2.3 Vibrational Dynamics of Water: Population Dynamics

#### 2.3.1 Vibrational Lifetime and Energy Transfer Dynamics

Pump–probe experiments established an OH-stretching lifetime *T*_1_ of ∼ 700 fs (HOD in D_2_O, 298 K) with an anomalous temperature dependence.^171^ A refinement of the energy flow was provided by frequency-resolved pump–probe spectroscopy using narrow-band excitation pulses^172^ showing that the *T*_1_ lifetime of the OH band varies monotonically in a range of 250–550 fs, depending on the excitation wavelength (3100–3700 cm^–1^). In this view, strongly H-bonded OH oscillators show a faster relaxation than oscillators that are weakly bound due to the better Fermi-resonance overlap with the 2ν_2_ overtone state of the HOH bending vibration. The monotonic frequency dependence of lifetime *T*_1_ reveals sub-picosecond structural heterogeneities in bulk water and provides a frequency-resolved relaxation map for surfaces and in electrolyte solutions.

A mapping of the energy transfer cascade from the initially excited OH stretching vibration revealed the vibrational energy relaxation in a stage-wise process, involving the bend vibration with a 170–180 fs lifetime^60,64^ and a downstream pathway via librations (∼0.5 ps) into the low-frequency bath.^90^ Thermalization is complete within ∼ 3 ps, leading to the formation of a ‘hot ground state’ where vibrational degrees of freedom are equilibrated but thermally elevated with a characteristic, blue-shifted absorption signature as background in pump–probe and 2D-IR experiments.

Dissolved ions perturb not only the structure and phase dynamics of water but also its population relaxation pathways and energy flow. In dilute solutions of simple monovalent salts, pump–probe and 2D-IR measurements generally find only modest changes in the OH-stretch lifetime,^173,174^ consistent with energy dissipation that still proceeds predominantly into the surrounding hydrogen-bond network, with perturbations largely confined to the first hydration shell. In contrast, strongly hydrated multivalent cations and strongly coordinating anions can open additional relaxation channels and redistribute excess energy more efficiently into librational and intermolecular modes, leading to shortened stretch lifetimes and altered branching between intramolecular and solvent modes.^174–176^ Similar to OH^–^,^177,178^ the hydration complex of H^+^ can be considered as an extreme case: Due to the spectral overlap with the librational band and the high fluxionality of the H^+^ double well potential, the lifetime of the PTM around 1200 cm^–1^ is largely reduced, estimated on the order of 20–40 fs.^142^ At higher ionic strengths, and especially in water-in-salt electrolytes,^158,179,180^ vibrational energy flow increasingly proceeds through ion-dominated local environments and collective ion–water modes, so that the notion of a single “bulk-like” water bath breaks down, and population dynamics become strongly heterogeneous and composition-dependent.

#### 2.3.2 Charge Transfer and Ionization of the H-Bond

The charge-transfer contribution to the H-bond, predicted theoretically by Torii and co-workers,^181^ was recently highlighted by correlated vibrational spectroscopy.^130^ By measuring the low-frequency vibrational mode of the translational displacement of water (∼180 cm^–1^), the results suggest that OH^–^ donates more negative charge density to the H-bond network than is accepted by H_3_O^+^ (8% vs 4%).

At the extreme end, the charge-transfer character of the H-bond underlies the observed behavior of liquid water subject to strong THz fields (on the order of a few MV/cm). The observation of a threshold behavior in the field-induced ionization of liquid water^182^ and associated long-lived polaron oscillations in water^183^ and polar solvents^184^ is consistent with a picture where strong intermolecular electric fields arising from thermally fluctuating motions (on the order of up to 100 MV/cm) lead to the transient and reversible partial charge transfer within the H-bond network. Ionization becomes irreversible under the external THz field, leading to the generation of a transient photocurrent. Subsequent subpicosecond localization of the generated, delocalized excess electron excites a quasi-particle of electron–nuclear motion, i.e., a polaron wavepacket oscillating at low THz frequencies (0.2–1.5 THz). The weak damping of the oscillatory dynamics suggests that formed polarons are of predominantly longitudinal character and couple only weakly^185^ to the fluctuating forces from the solvated electron environment, leading to long-lived (ps) oscillatory dynamics.

The recent experiments thus support a picture where the charge transfer contribution to the H-bond beyond pure electrostatics, i.e., the partial donation of electron density from the lone pair of the H-bond acceptor to the σ* antibonding orbital of the OH bond of the donor, induces the large redshift of the OH stretch band upon H-bond formation. This effect also leads to a substantial increase in the IR transition strength (non-Condon effects). Schmidt et al. quantified the magnitude of these effects for liquid water and showed that they are pronounced: because the OH-stretch transition dipole grows with hydrogen-bond strength, red-shifted, strongly hydrogen-bonded molecules are systematically overweighted in the infrared spectrum.^186^ Any structural interpretation of spectral amplitudes, at interfaces no less than in bulk, must therefore correct for this frequency-dependent oscillator strength before populations are inferred.

### 2.4 The Air–Water Interface

The air–water interface serves as the canonical hydrophobic boundary and therefore provides a reference against which more complex hydrophobic interfaces, such as graphene–water, oil–water, and membrane–water systems (Section 3) and solid–liquid interfaces (Section 4) can be compared. Early homodyne SFG measurements established the characteristic spectral features of interfacial water, including the narrow band near 3700 cm^–1^ assigned to dangling OH oscillators and the broad hydrogen-bonded OH stretch extending from approximately 3000 to 3600 cm^–1^. These features arise from reduced hydrogen-bond coordination and surface orientational anisotropy. Phase-sensitive and heterodyne-detected SFG later established that the sign of the imaginary χ^(2)^ reports the absolute orientation of interfacial OH transition dipoles, providing direct access to the directionality of interfacial hydrogen bonds (Figure 6).^18,187^ At the neat air–water interface, where static interfacial fields are minimal and diffuse layer contributions are absent, this phase-sensitive response provides one of the cleanest measurements of the intrinsic second-order vibrational response of interfacial water.^188–190^ Thus, the air–water interface is a critical reference for interpreting more complex aqueous interfaces, where chemical functionality, charge, confinement, or condensed hydrophobic phases can alter both the structure of interfacial water and the measured nonlinear response.^82,83,191^

**Figure 6:**
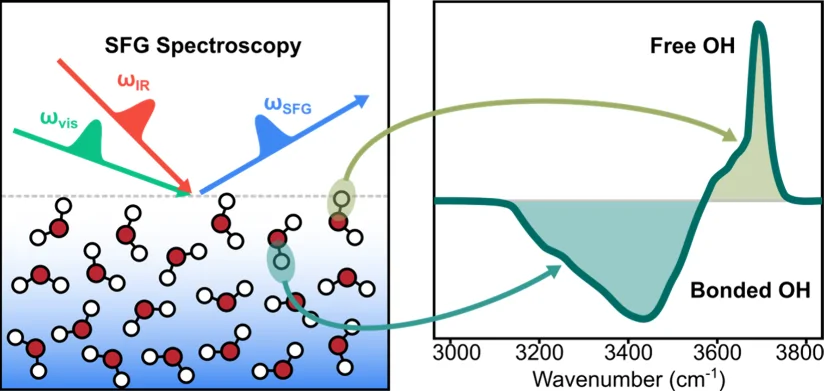
Schematic of an SFG experiment: visible (*ω*_vis_) and infrared (*ω*_IR_) beams overlap at the air–water interface and generate a signal at *ω*_SFG_ = *ω*_vis_ + *ω*_IR_. (right) Im χ^(2)^ spectrum of the air–water interface in the OH stretching region obtained by HD-SFG. The sign reports the net orientation of the OH groups: the positive narrow band near 3700 cm^–1^ arises from free (dangling) OH groups pointing toward the vapor phase, and the broad negative band arises from hydrogen-bonded OH groups with net orientation toward the bulk.

None of these spectral assignments could have been made without a parallel theoretical effort. The foundational analysis of Morita and Hynes^125,192^ established how the SFG spectrum of the water surface follows from the molecular hyperpolarizability and the orientational distribution of interfacial molecules, providing a foundation for subsequent simulations of interfacial vibrational spectra; the field as a whole has been reviewed by Ishiyama et al.^193^ Building on this framework, Skinner et al. showed that mixed quantum/classical treatments with electrostatic frequency maps reproduce the SFG line shape of water next to lipid and surfactant monolayers,^170,194^ and later extended the same machinery to the water bend mode,^92^ whose interfacial response is also sensitive to hydrogen-bond geometry. The predictive power of these approaches is not limited to spectroscopy: Ishiyama, Tahara, and Morita used the same surface-hydration picture to explain why the photochemistry of phenol accelerates dramatically at the air–water interface, illustrating that spectroscopically calibrated interfacial structure carries genuine chemical consequences.^195^ This dramatic acceleration has, however, recently been called into question by ultrafast photoelectron measurements, which find no evidence for correspondingly enhanced electron ejection from interfacial phenol.^196^

The air–water interface also served as the primary test system for extending nonlinear vibrational spectroscopy to the time domain. As a result, much of what is currently known about the dynamics of interfacial hydration has been established from ultrafast measurements at the air–water interface, which provide a reference for interpreting more complex hydrophobic and heterogeneous systems. Time-resolved heterodyne-detected SFG measurements^197,198^ showed that the dangling OH vibration relaxes on a time scale of approximately 0.8–0.9 ps with little isotope dependence, indicating that the observed anisotropy decay is governed primarily by molecular reorientation, that contributions from intermolecular vibrational energy transfer are negligible, and that vibrational relaxation occurs on a comparable time scale.^199^ Measurements of the hydrogen-bonded OH stretch region^200,201^ reported significantly shorter lifetimes (0.2–0.4 ps), comparable to the OH stretch vibrational lifetime of bulk water,^202^ demonstrating that interfacial water retains many aspects of the bulk-like intramolecular relaxation while being structurally anisotropic.

Multidimensional spectroscopies provide further insight into the heterogeneity of interfacial hydration. Two-dimensional heterodyne-detected SFG measurements of water at the air–water interface by Singh and co-workers^203^ revealed heterogeneous spectral diffusion within the interfacial hydrogen-bond network, with diagonal peak elongation decaying within a few hundred femtoseconds, indicating ultrafast structural fluctuations and vibrational coupling among distinct interfacial OH species. Complementary time-resolved infrared and HD-SFG measurements by van der Post and co-workers^172^ showed that the strong frequency dependence of the vibrational relaxation of the OH stretch is preserved for interfacial water, with even larger dispersion at the air–water interface. The results demonstrate that subpicosecond structural heterogeneity persists despite rapid spectral diffusion, providing an important bridge between 2D-IR benchmarks of bulk water and surface-specific multidimensional measurements. Although 2D-IR spectroscopy is not intrinsically surface-specific, bulk measurements^3,5^ (cf. Section 2.2) provide an important reference for the hydrogen-bond dynamics at interfaces. Observed spectral diffusion timescales in surface-specific measurements are generally very comparable to the spectral diffusion in bulk water. Together, these studies establish the air–water interface as a benchmark system for understanding the dynamical aspects of hydrophobic hydration and provide a basis for interpreting ultrafast measurements at graphene–water, oil–water, and membrane–water interfaces.

### 2.5 Bridging Bulk and Interfacial H-Bond Dynamics: A Mechanistic Perspective

The observation—illustrated in the following sections—that spectral diffusion timescales at some interfaces are comparable to those of bulk water raises a fundamental mechanistic question: Why does the hydrogen-bond network reorganize at similar rates despite the broken symmetry, reduced coordination, and altered electrostatic environment at interfaces? For the OH stretch of dilute HOD in D_2_O, sub-200-fs features of the frequency–frequency correlation function reflect local hydrogen-bond fluctuations and underdamped intermolecular motion, whereas the ∼ 1.4 ps relaxation reflects collective reorganization of the liquid.^145^ At the air–water interface, water molecules in the topmost layer exhibit dangling OH groups, asymmetric hydrogen bonding, and preferential orientation, yet ultrafast 2D-SFG measurements reveal spectral diffusion on comparable subpicosecond to picosecond timescales.^198,203,204^

This similarity suggests that the local topology of hydrogen-bond rearrangement—involving librational motions, angular jumps, and partner exchange^205^ remains fundamentally similar at hydrophobic interfaces, even when the average coordination and orientation differ from bulk. The fastest component (∼100–200 fs) likely reflects librational sampling of local minima within a given hydrogen-bond configuration, a process governed by intermolecular restoring torques and short-range electrostatic fluctuations that are only weakly perturbed by interfacial boundary conditions. The slower component (∼1–3 ps) corresponds to hydrogen-bond breaking and reformation, which requires sufficient thermal energy and molecular reorientation.^27^ At hydrophobic interfaces, the reduced coordination number might be expected to accelerate this process; however, the orientational constraints imposed by surface geometry and the need to maintain connectivity with the underlying bulk network may partly compensate. Establishing this balance requires comparison of the same vibrational reporter and analysis model across environments.

Deviations from bulk-like dynamics should therefore emerge when interfacial perturbations become sufficiently strong to alter either (i) the energetic barriers for hydrogen-bond rearrangement or (ii) the available configurational pathways. Strong electrostatic fields, such as those at highly charged interfaces or in the presence of multivalent ions or surface-active species, can significantly increase the orientational barriers and slow spectral diffusion. Similarly, nanoconfinement within rigid hydrophobic pores introduces geometric frustration that restricts angular jump pathways and can lead to pronounced dynamic heterogeneity. Understanding when and why interfacial dynamics deviate from the bulk therefore requires systematic characterization of how local hydrogen bond topology, electrostatic perturbations, and geometric constraints combine to shape the effective energy landscape for structural rearrangement. This mechanistic framework provides a foundation for interpreting more complex soft, solid, and confined interfaces, as discussed in subsequent sections.

## 3 Soft Aqueous Interfaces

### 3.1 Concept and Scope of Soft Aqueous Interfaces

The molecular-level insight provided by ultrafast nonlinear vibrational spectroscopy into the structure, dynamics, and reactivity of aqueous electrolyte solutions is similarly applicable to heterogeneous environments. Many important heterogeneous environments in chemistry involve interfaces,^206–210^ where symmetry, solvation structure, and electrostatic interactions differ fundamentally from those in the bulk.^211–213^ To establish molecular-level principles for these effects, it is useful to begin with soft aqueous interfaces, which we define here as aqueous boundaries whose interfacial structure remains molecularly mobile, deformable, and responsive to thermal fluctuations, solvation, adsorption, and changes in composition, rather than being fixed by a rigid crystalline substrate.^214,215^ This class includes vapor–liquid, liquid–liquid, surfactant- and lipid-covered, polymer-coated, and biomolecular interfaces, as well as weakly interacting or atomically thin materials such as graphene. Although many of these systems are compositionally and dynamically complex, they often avoid the fixed lattices, heterogeneous binding sites, surface reconstruction, electronic coupling, and substrate-dependent fields that complicate solid–liquid interfaces.^216–218^ They therefore serve as useful benchmark systems for isolating how water and dissolved ions reorganize in response to solvation, adsorption, local intermolecular interactions, and interfacial charge distributions.^15,16,219,220^

These interfaces are especially well-suited for nonlinear vibrational spectroscopy because their interfacial water, ions, and adsorbates generate surface-specific vibrational responses without many of the technical complications imposed by rigid substrates. SFG, HD-SFG, and multidimensional variants therefore provide direct access to molecular orientation, hydrogen-bond structure, ion-induced fields, and ultrafast interfacial dynamics in systems where water and solutes remain mobile and responsive to local interactions.

In the following subsections, we discuss how ultrafast nonlinear vibrational spectroscopy has been used to elucidate the properties of aqueous electrolyte solutions at soft interfaces. We first examine how hydrophobic and hydrophilic environments influence the structure and dynamics of interfacial water, followed by a discussion of ion adsorption, hydration, and the formation of interfacial charge distributions. We then consider how changes in temperature, salt concentration, and molecular composition can induce structural transitions in soft interfacial assemblies, and how these changes are reflected in vibrational spectra. Finally, we review recent studies of chemical reactivity at soft interfaces, including acid–base processes, organic and inorganic reactions, and microdroplet chemistry, with emphasis on the unique mechanistic insights provided by time-resolved and multidimensional nonlinear spectroscopic techniques. Together, the discussion herein illustrates how soft aqueous interfaces serve as model systems for understanding the fundamental behavior of electrolyte solutions at boundaries and provide a foundation for interpreting more complex solid and confined environments discussed in later sections.

### 3.2 Insights into Hydrophobicity at Interfaces

Hydrophobic and hydrophilic interactions strongly influence the structure, dynamics, and electrostatic properties of the interfacial water. By imposing different boundary conditions on the water hydrogen-bond network, they alter the orientational ordering, hydrogen-bond strength, and dielectric response within the first few molecular layers. As these perturbations are interfacial, surface-specific methods are required to resolve them. SFG and phase-resolved SFG provide direct information about interfacial water structure and orientation, while nonlinear vibrational scattering extends related measurements to curved or heterogeneous interfaces. Time-resolved SFG, two-dimensional SFG, and surface-sensitive 2D-IR spectroscopy further connect these structural motifs to the study of hydrogen bond dynamics and energy transfer, providing a molecular picture of hydrophobic hydration at soft and weakly interacting aqueous interfaces.

#### 3.2.1 The Graphene–Water Interface

Hydrophobic aqueous interfaces provide a stringent test of interfacial water structure by enabling direct comparisons between idealized vapor–liquid boundaries and chemically more complex soft condensed-phase environments. Graphene–water interfaces are particularly useful in this context because atomically thin carbon sheets present a weakly interacting, electronically polarizable surface while preserving a well-defined planar geometry. Unlike macroscopic hydrophobic solids, graphene provides a well-defined, atomically smooth interface that enables systematic investigation of water structure and electrostatic response at a weakly interacting boundary.

Experimental studies of water at graphene interfaces have largely begun with supported graphene, which provides experimental accessibility but does not fully isolate the intrinsic graphene–water interaction. Surface-sensitive vibrational spectroscopy of water and ice adjacent to single-layer graphene^221^ showed that the hydrogen-bonded OH spectrum near graphene is broadly similar to bulk or air–water, with only subtle modifications. Surface-specific SFG measurements at potentiostatically controlled supported graphene electrodes^222^ further demonstrated that under open-circuit conditions, the interfacial spectrum closely resembles that of the air–water interface, whereas applied potentials induce field-driven reorientation of interfacial water. A later study by Montenegro et al.^223^ reported an asymmetric response of interfacial heavy water to applied electric fields at a CaF_2_-supported graphene electrode and assigned a high-frequency feature observed at negative bias to interfacial non-hydrogen-bonded OD oscillators. However, this interpretation was subsequently challenged by Wang et al.,^224^ who used heterodyne-detected SFG to show that the sign of the corresponding spectral feature is negative and therefore consistent with CaOH species associated with the CaF_2_ substrate rather than interfacial water. These results demonstrate that supported graphene constitutes a composite interface in which substrate chemistry and electrostatic boundary conditions can contribute to the observed nonlinear response. Together, supported graphene studies establish that the hydrogen-bond network near graphene is broadly similar to that at the air–water interface under neutral conditions, but becomes highly sensitive to electrostatic perturbations and substrate contributions when the interface is charged or biased.

To isolate the intrinsic graphene–water interaction, several studies have focused on suspended graphene, in which the carbon sheet spans a liquid aperture and is not in direct contact with a solid support. Early theoretical work using ab initio MD combined with simulated SFG spectra predicted that the vibrational response of water at the graphene–water interface should closely resemble that at the air–water interface, with a dangling OH (OD) feature indicative of hydrophobic character and only modest shifts in the hydrogen-bonded region.^225^ These simulations also showed that the presence of water on both sides of a supported graphene sheet can partially cancel the SFG response, explaining earlier experimental reports of a reduced free OH signal and highlighting the importance of studying free-standing interfaces. Direct experimental realization of such an intrinsic boundary was achieved using heterodyne-detected SFG at suspended graphene–water interfaces (Figure 7).^84^ In this geometry, the graphene membrane freely sits on the interface, eliminating substrate contributions while preserving a well-defined planar interface. Phase-resolved measurements showed that the imaginary χ^(2)^ spectrum of interfacial water is nearly identical to that of the air–water interface, with only small shifts in the free OH frequency. The close correspondence of the spectra indicates that graphene perturbs only the outermost molecular layer and does not significantly restructure the underlying hydrogen-bond network, demonstrating that a single atomic layer of carbon behaves as a weakly interacting hydrophobic boundary. Electrochemically tunable suspended graphene interfaces provide an important test of the robustness of hydrophobic hydration to external perturbations. Measurements at gate-tunable suspended graphene–water interfaces^226^ showed that the vibrational spectrum of interfacial water changes only modestly within the electrochemical stability window, indicating that the hydrogen-bond network characteristic of hydrophobic interfaces is largely preserved under moderate electrostatic perturbations. Pronounced restructuring occurs only once Faradaic processes are initiated, demonstrating that significant deviations from hydrophobic hydration arise primarily from chemical reactions rather than from the intrinsic graphene–water interaction. Theoretical predictions, heterodyne-detected SFG measurements, and electrochemically controlled experiments establish that suspended graphene provides a well-defined model of the hydrophobic solid–water interface.

**Figure 7:**
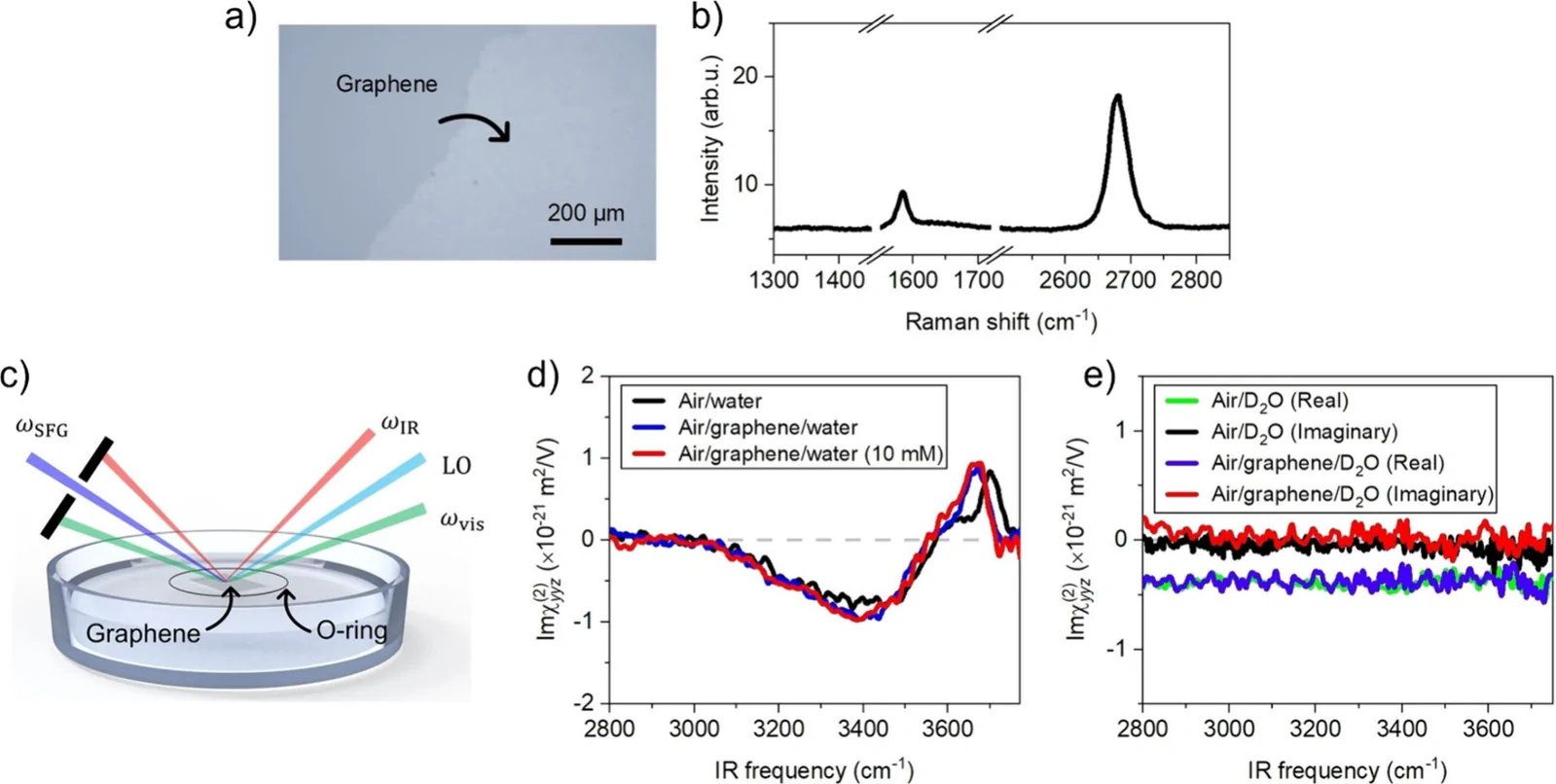
Heterodyne-detected vibrational sum frequency generation of interfacial water at suspended graphene. (a) Optical image of monolayer graphene suspended at the air/water interface. (b) Raman spectrum confirming high-quality suspended graphene. (c) Schematic of the HD-SFG geometry used to probe the buried graphene/water interface. (d) Imaginary HD-SFG spectra of interfacial H_2_O at the air/water interface, the suspended graphene/water interface, and the suspended graphene/water interface after addition of 10 mM NaCl. The close similarity between the air/water and graphene/water spectra indicates that suspended graphene only weakly perturbs the interfacial hydrogen bond network. (e) Real and imaginary HD SFG spectra of D_2_O at the air/D_2_O and suspended graphene/D_2_O interfaces, showing that the graphene contribution is negligible in the OH/OD stretching region. Reproduced with permission from Y. Wang et al. *Angew. Chem., Int. Ed.* 2024, 63, e202319503,^84^ under the terms of the Creative Commons CC BY 4.0 license. Copyright 2024 The Authors. Published by Wiley VCH GmbH.

At present, however, time-resolved SFG and multidimensional measurements at suspended graphene–water interfaces remain largely unexplored. Extending ultrafast heterodyne-detected and two-dimensional spectroscopies to atomically thin materials will be essential for determining whether the dynamical behavior of hydration at graphene mirrors that of the air–water interface or exhibits distinct signatures associated with electronic polarizability and interfacial confinement. Establishing such comparisons will be critical for understanding hydration at weakly interacting solid boundaries and for linking structural and dynamical descriptors of hydrophobicity across different classes of aqueous interfaces.

#### 3.2.2 The Oil–Water Interface and Hydrophobic Hydration

The oil–water interface is an important hydrophobic boundary, but unlike the air–water interface, it forms between two condensed phases. It is useful to distinguish neat oil–water interfaces from oil–water interfaces modified by surfactants, lipids, or polymers, because the latter introduce headgroup-specific interactions that can dominate the interfacial response. Early reflection-geometry vibrational SFG measurements demonstrated that the structure of interfacial water at neat oil–water boundaries differs from that at the vapor interface. Gragson and Richmond compared neat oil–water and air–water interfaces and showed that the OH stretch spectrum at the CCl_4_–water boundary is distinct from that at air–water, indicating that the hydrogen-bond environment at this liquid–liquid interface is not identical to that at the vapor interface.^227^ Brown et al. later extended these measurements to alkane–water interfaces and found that the spectral response depends on the organic phase and interfacial conditions, consistent with changes in orientational ordering and hydrogen-bonding structure at the buried liquid–liquid boundary.^228^ Subsequent broadband femtosecond SFG measurements by Strazdaite, Versluis, Backus, and Bakker showed that D_2_O/hexane, D_2_O/heptane, and D_2_O/PDMS interfaces exhibit substantially enhanced SFG intensities relative to D_2_O/air, which was interpreted as stronger orientational ordering of interfacial water and stronger hydrogen bonding at these hydrophobic boundaries.^229^ This result differs from earlier narrowband measurements on water/CCl_4_ and water/alkane interfaces and highlights that the inferred hydrogen-bond structure at oil–water interfaces can depend sensitively on the organic phase, sample preparation, temperature, and experimental implementation. Raman spectroscopy in tandem with multivariate curve resolution^230^ recently suggested strong interfacial electric fields on the order of 60 MV/cm at oil–water emulsion interfaces with reduced tetrahedral order and weakened hydrogen bonding. Additional studies of oil–water interfaces containing soluble surfactants showed that the interfacial spectrum is highly sensitive to interfacial charge density, demonstrating that once surfactants or lipids are present, electrostatic fields, headgroup chemistry, ion binding, and lateral packing can all strongly modify the nonlinear response, even at interfaces that are otherwise hydrophobic.^231^ SFG measurements of surfactant monolayers at oil–water interfaces required specialized optical geometries to isolate the buried interface and showed that charged or polar headgroups can strongly modify the OH response of interfacial water.^232^ Additional nonlinear scattering studies on lipid- and polymer-coated oil droplets likewise showed that different interfacial compositions lead to substantially different water responses, and that adsorption of polycations or ions can reorganize the interfacial region.^233–235^ These coated systems are highly relevant as model systems for emulsions, lipid droplets, and biological membranes. Nevertheless, their relevance as direct probes of pure hydrophobic hydration at neat oil–water boundaries is limited because the nonlinear response reflects a combination of hydrophobic interactions, electrostatic fields, and headgroup-specific chemistry.

As planar buried interfaces are experimentally demanding and often sensitive to wetting and contamination, vibrational SFS has become a widely used approach for studying hydrophobic hydration at oil–water interfaces. Vácha et al. combined SFS, zeta-potential measurements, and MD simulations to show that water molecules at neat hydrophobic oil droplets are preferentially oriented with their hydrogen atoms pointing toward the oil phase, similar to negatively charged interfaces, while finding no evidence for selective hydroxide adsorption.^236^ Follow-up SFS measurements on alkane nanodroplets showed that the vibrational response is largely pH-insensitive and does not exhibit clear hydroxyl-ion signatures, arguing against a simple OH^–^-adsorption picture for the apparent negative charging of hydrophobic droplets.^237^ Isotopic-dilution SFS experiments further showed that the hydrogen-bond network at hydrophobic droplet surfaces can be as strong as, or stronger than, that of bulk water and differs in important ways from the air–water interface.^238^ Pullanchery et al. proposed that these observations may reflect interfacial charge transfer mediated by weak C–H···O hydrogen bonds at the oil–water boundary, suggesting a possible microscopic origin for the stability and spectroscopic signatures of hydrophobic droplets in water.^238^ This interpretation is interesting, but it should be treated cautiously because the microscopic origin of the apparent interfacial charge remains under debate. Methodological work comparing reflection SFG and scattering SFS further showed that the two geometries can probe different effective interfacial responses because of phase-matching constraints and absorption effects, underscoring the need for care when comparing planar and droplet measurements.^239,240^

### 3.3 Ion Adsorption at Soft Interfaces

At hydrophobic interfaces in the absence of ions, the structure and dynamics of interfacial water are primarily governed by hydrogen bonding and symmetry-breaking. In most realistic environments, however, interfaces are in contact with electrolyte solutions, and dissolved ions introduce additional complexity through adsorption, hydration, and electrostatic interactions. These processes generate interfacial electric fields, modify the hydrogen-bond network, and influence both the structure and dynamics of interfacial water. At a molecular level, ion behavior reflects a balance between hydration and interfacial interactions, leading to ion-specific distributions that often deviate from mean-field descriptions. Nonlinear vibrational spectroscopies are uniquely suited to probe these effects, as they provide sensitivity to both molecular orientation and local electric fields. They can distinguish between intrinsic interfacial structure and field-induced responses. In this section, we first examine ion adsorption at the air–water interface as a model system. Recent results challenge the conventional picture of surface enrichment. We then consider specific ion effects on hydration structure, followed by a discussion of electric double-layer formation and interfacial fields. Finally, we extend these concepts to biomolecular and molecularly coated interfaces, where ion binding and electrostatics play central roles in determining interfacial structure and function.

#### 3.3.1 Ion Adsorption at the Air–Water Interface

The air–water interface provides the canonical model system for understanding ion adsorption at aqueous boundaries, where broken symmetry, reduced coordination, and interfacial electrostatics lead to ion distributions that differ from those in the bulk. Classical continuum electrostatic descriptions, beginning with the work of Onsager and Samaras, predicted that ions should be depleted from the air–water interface because image-charge repulsion makes it energetically unfavorable for charge to approach the low-dielectric vapor phase.^241^ This framework provided the canonical interpretation of electrolyte surface tension measurements, in which increasing salt concentration raises the surface tension of water. At dilute concentrations, however, the Jones–Ray surface tension minimum suggested an early deviation from this depletion picture. Anion surface enhancement was reported from resonance-enhanced second-harmonic generation (SHG) in the same millimolar concentration range, supporting the idea that dilute electrolyte interfaces can exhibit positive surface excess despite the classical expectation of ion depletion.^242^ This interpretation was later summarized in a review, which noted that the increase in surface tension was traditionally taken to imply negative surface excess and an outermost interfacial layer that is largely devoid of inorganic ions, although this picture was increasingly challenged by chemical kinetics, polarizable molecular dynamics simulations, and surface-sensitive nonlinear optical measurements.^243^ In the early 2000s, however, this view was revised by MD simulations and surface-specific spectroscopy. Early MD simulations suggested that large, polarizable anions such as I^–^ exhibit enhanced surface propensity and can increase ordering in subsurface water, leading to measurable changes in the SFG response of the hydrogen-bonded OH stretch region.^244^ These predictions helped to establish the view that ion-specific adsorption can be inferred from the vibrational response of interfacial water. Direct experimental evidence for ion-specific interfacial enrichment came from phase-sensitive SFG measurements of acidic and basic aqueous solutions that showed the accumulation of H_3_O^+^ and I^–^ at the air–water interface. Their presence modifies the imaginary component of χ^(2)^, with H_3_O^+^ generating a positive surface field that enhances the lower-frequency hydrogen-bonded OH response.^245^ In contrast, OH^–^ showed much weaker interfacial propensity. These results established that the SFG response of electrolyte solutions reflects not only ion distributions but also the field-induced alignment of interfacial water.

For simple salts, however, the spectroscopic signatures are often more subtle than those suggested by this picture. Feng et al. found that NaF produces only modest changes in the free-OH response, corresponding to a relatively small change in the average interfacial OH tilt angle.^246^ Such behavior is consistent with strong ion hydration and weak surface affinity. More broadly, the classic framework summarized by Jungwirth and Tobias distinguishes strongly hydrated, surface-depleted ions from larger, more polarizable ions with a greater interfacial propensity.^219^

Recent heterodyne-detected SFG measurements have refined the simple distinction between surface-enriched and surface-depleted ions. The combination of HD-VSFG with neural-network-assisted ab initio MD enabled examination of a broad set of simple electrolyte solutions,^247^ showing that NaCl, NaOH, and CsF strongly modify the hydrogen-bonded OH region while leaving the free-OH peak near 3700 cm^–1^ largely unchanged. These findings are in contrast to HCl, where proton adsorption directly suppresses the free-OH feature. Since the free-OH band reports on the outermost air-facing water molecules, this spectral pattern indicates that many ions do not substantially populate the topmost interfacial layer. Simulations that reproduce the measured spectra further show ion density maxima below the surface, supporting a stratified interfacial structure in which an ion-depleted outermost layer overlies an ion-enriched subsurface region.^247^ This behavior shows that the electrolyte-induced SFG response cannot be explained solely by a unidirectional electric-double-layer alignment, but instead reflects depth-dependent ion distributions and water reorientation.

This revised interpretation is especially important because it changes how SFG spectra recorded at electrolyte solution interfaces should be interpreted. In the stratification picture, the interfacial response reflects the combined contributions of water molecules influenced by nearby cations and anions in the subsurface region, rather than simply a net field generated by a sharply separated ionic double layer. Litman et al. further deconvoluted the spectra into cation- and anion-associated contributions and showed that the anionic contribution redshifts systematically along the halide series I^–^ → Br^–^ → Cl^–^ → F^–^, consistent with increasing hydrogen-bond strength. They also concluded that only particularly surface-active species, such as hydrated protons and perchlorate, exhibit behavior more consistent with an EDL-like description at the air–water boundary.^247^

The air–water interface has also served as the benchmark against which hydrophobic solid–water interfaces are compared. Heterodyne-detected SFG measurements of suspended graphene–water interfaces showed that the OH spectrum is nearly identical to that of the air–water interface under neutral conditions, indicating that graphene minimally perturbs the interfacial hydrogen-bond network.^84^ Yet, when ions are introduced, the analogy breaks down. Advincula et al. found that NaCl can accumulate strongly at a graphene–water interface while producing only minimal spectral changes, demonstrating that ion accumulation does not necessarily lead to the pronounced water restructuring typically inferred at the air–water interface.^248^ Finally, as discussed in more detail below, recent time-resolved SFG studies have shown that genuine electric double-layer dynamics can be observed at air–electrolyte interfaces when specific surface-active ions induce substantial interfacial charge separation.^249^

#### 3.3.2 Interface-Dependent Ion Specificity and Hydration Dynamics

Having established the air–water interface as the benchmark for ion adsorption, we now consider how ion specificity changes across soft aqueous interfaces and how ions modify interfacial hydration dynamics. Specific ion effects are commonly rationalized through the Hofmeister framework, in which ion size, polarizability, and hydration thermodynamics determine the balance between bulk solvation and interfacial stabilization.^219^ Large, highly polarizable anions such as I^–^, SCN^–^, and ClO_4_^–^ generally show greater interfacial propensity than small, strongly hydrated ions such as F^–^, Na^+^, and Mg^2+^. Theoretical treatments by Levin and co-workers showed that ion polarizability can reduce the penalty associated with electrostatic image-charge repulsion at dielectric boundaries, allowing large anions to approach or populate interfaces.^220,250–252^ However, the connection between ion adsorption and the vibrational response of water is not universal. Across oil–water, graphene–water, surfactant-covered, and charged interfaces, this relationship depends on interfacial polarity, geometry, polarizability, and the depth distribution of ions.

Surface-specific vibrational spectroscopies have provided direct experimental access to these effects. Early SFG and phase-sensitive SFG studies established that ions modify the interfacial hydrogen-bond network primarily by altering water orientation and local electric fields.^212,253,254^ For example, hydronium and large anions produce pronounced changes in the hydrogen-bonded OH region, consistent with enhanced interfacial electric fields and reorientation of interfacial water.^245^ In contrast, strongly hydrated ions such as Na^+^ and F^–^ induce comparatively small perturbations to both the free-OH and hydrogen-bonded spectral regions. These observations are broadly consistent with classical Hofmeister trends, in which chaotropic ions are more surface active than kosmotropic ions.

The extent to which specific ion effects depend on the nature of the interface is further illustrated by comparisons across different soft interfaces. At oil–water interfaces, resonantly enhanced nonlinear spectroscopy and MD simulations show that ion adsorption can proceed through a mechanism distinct from that at the air–water interface. Devlin et al. found that SCN^–^ adsorption at water–toluene and water–decane interfaces has Gibbs free energies similar to those at air–water, but is driven primarily by entropy through water-finger formation rather than by favorable adsorption enthalpy.^255^ More recently, Devlin et al. used planar liquid-sheet deep-UV SHG to compare SCN^–^ adsorption at air–water and water–heptane interfaces directly, showing that adsorption at air–water is enthalpy-driven and entropy-opposed, whereas adsorption at water–heptane is entropy-driven and enthalpy-opposed.^256^ Similarly, studies of graphene–water interfaces demonstrate that ion accumulation does not necessarily lead to large perturbations in interfacial water structure, indicating that the relationship between ion adsorption and spectroscopic response depends sensitively on interfacial geometry and polarizability.^257^ These results underscore that specific ion effects are not universal but instead reflect the interplay between ion properties and the physical characteristics of the interface.

Complementary ultrafast spectroscopies provide insight into how ions influence hydration dynamics. For example, Piatkowski et al. used time-resolved 2D-SFG of NaCl and NaI solutions in D_2_O to show that halide ions slow resonant vibrational energy transfer between interfacial OD groups, consistent with reduced effective water density at the surface caused by ion enrichment.^258^ From the 2D-SFG slope dynamics, they estimated surface chloride and iodide concentrations to be approximately 1.8 and 3.5 times higher than the bulk concentration, respectively.^258^ Bulk 2D-IR measurements show that small, strongly hydrated ions slow hydrogen-bond rearrangement by restricting angular jump pathways, whereas larger, weakly hydrated ions have weaker effects on spectral diffusion.^259^ At interfaces, time-resolved and multidimensional SFG measurements reveal that hydrogen-bond dynamics remain ultrafast but become spatially heterogeneous and sensitive to local electric fields. Two-dimensional HD-SFG studies of charged interfaces have shown spectral diffusion on subpicosecond time scales (∼200–300 fs), reflecting rapid fluctuations of the interfacial hydrogen-bond network under the influence of ionic fields.^260^ Likewise, time-resolved SFG measurements of the air–water interface show that the free-OH mode relaxes on a time scale of ∼ 0.8–0.9 ps, largely through molecular reorientation.^197^ Together, these results indicate that specific ions modify not only the static structure of interfacial water but also the pathways and time scales of hydrogen-bond rearrangement.

#### 3.3.3 Electric Double Layer Formation and Interfacial Fields

Interfaces with asymmetric ion distributions generate static electric fields that polarize interfacial water. The result is an EDL: a fixed layer of surface charge and a diffuse layer of counter-ions. An EDL can form even at weakly charged interfaces; its spectroscopic detectability depends on the surface charge, electrolyte concentration, and optical geometry. A nominally neutral interface such as neat air–water need not exhibit a measurable diffuse-layer contribution.

In the classical EDL model, a compact Stern layer of adsorbed ions resides at the surface, screened by a diffuse Gouy–Chapman layer extending into the solution. The resulting electrostatic potential breaks inversion symmetry at the interface. As Shen first noted, a noncentrosymmetric bulk (due to a DC field) contributes to SFG via a third-order polarization.^261^ In practice, the measured SFG response is

5 $$\chi_{eff}^{\left(2\right)}=\chi^{\left(2\right)}+\chi^{\left(3\right)}\int_{z_{a}}^{\infty }dzE_{DC}\left(z\right)e^{i\Delta k_{z}z}$$

where χ^(2)^ and χ^(3)^ reflect the second-order and third-order contributions, which arise from the Stern layer and diffuse layer, respectively;^262^ *E*_DC_(*z*) is the depth-dependent static electric field within the EDL; Δ*k_z_* is the wave-vector mismatch along the surface normal; and *z*_a_ defines the boundary between the Stern layer and the diffuse layer. Pezzotti et al. further formalized this distinction by separating charged aqueous interfaces into a binding interfacial layer and a diffuse layer, showing that the diffuse-layer SFG contribution arises from field-induced reorientation of bulk-like water and can be described through a χ^(3)^ response.^263^ The SFG response of an EDL can be field-dependent because the measured spectrum may contain both an intrinsic χ^(2)^ contribution and a field-induced χ^(3)^ contribution from water polarized by the interfacial electrostatic field. In heterodyne-detected SFG, changes in pH or applied bias (which alter the EDL) are observed as sign or amplitude changes in the imaginary part of the effective second-order susceptibility.^189^ For example, Wen et al. showed that titrating a silica/water interface changes the OH-stretch spectrum dramatically, flipping its sign as the surface field reverses.^83^ Ohno et al. demonstrated quantitatively that only by including the field-induced third-order component can one reproduce such “second-order” spectral lineshapes at charged interfaces.^264^ In the OH-stretch region, these measurements primarily report the water response rather than ions directly; vibrationally active molecular ions can also be probed through their own resonances.

The χ^(3)^-based EDL description applies well to strongly charged systems, such as metal electrodes, oxide surfaces, cationic lipid monolayers, and polyelectrolytes, where fixed charges create robust Stern and diffuse-layer structures. In these cases, the field-induced contribution can be substantial; extracting charge densities requires separating it from the intrinsic interfacial response and specifying an electrostatic model.^265,266^ By contrast, at neutral or weakly charged boundaries (e.g., the pristine air–water interface or suspended hydrophobic solids), most salts do not produce a long-range diffuse layer. Recent nonlinear spectroscopy shows that simple electrolytes at a clean water surface remain subsurface; their fields are largely screened within a molecular layer, so the contribution to the free-OH feature is negligible.^15^ The absence of a resolved field-induced contribution should therefore not be taken as proof of zero surface charge; short-range hydration and diffuse-layer contributions must be assessed separately.

Time-resolved SFG studies of EDL dynamics remain relatively scarce because small transient changes in the OH response are easily obscured, requiring exceptional phase stability over prolonged measurements. After a perturbation, such as a rapid change in pH or temperature, the interfacial potential relaxes primarily through ion conduction as the EDL re-establishes electrostatic screening. Experiments indicate that water dipoles reorient on subpicosecond time scales,^116,198,204^ while ion redistribution occurs over several picoseconds.^267–269^ Greco et al.^249^ heated an air/water interface and tracked how excess H_3_O^+^ and Cl^–^ re-establish the double layer: water orientation shifted immediately (≲1 ps), whereas full ionic equilibration took a few picoseconds. These results mirror bulk Debye-screening dynamics: the EDL relaxes on ion-conduction time scales, confirming that SFG-detected field changes track real-space ion motion.

#### 3.3.4 Water Interacting with Lipids and Surfactants

Lipid monolayers and membranes provide a uniquely well-defined class of soft aqueous interfaces in which electrostatics, molecular packing, and hydration are strongly coupled. Unlike neat air–water or simple hydrophobic interfaces, lipid assemblies possess chemically specific headgroups that can be zwitterionic, anionic, or cationic, leading to fixed interfacial charge densities and well-defined boundary conditions for ion adsorption and water organization.^271,272^ The sign and chemistry of the headgroup charge are especially important: water at positively charged lipid interfaces can exhibit dynamics that resemble bulk water more closely, whereas negatively charged and zwitterionic lipid interfaces produce more distinct interfacial water responses. This difference has been attributed to charge-induced alignment at cationic lipid interfaces, where interfacial O–D groups preferentially point toward the bulk aqueous phase.^273^ In addition, the lateral packing of lipid molecules creates a quasi-two-dimensional fluid with tunable density and phase behavior, allowing systematic control over intermolecular spacing and surface potential.^274^ More recent work on phospholipid monolayers further shows that even nominally neutral (zwitterionic) headgroups can induce substantial restructuring of interfacial water, highlighting the importance of local headgroup composition and packing constraints.^275,276^ As a result, lipid monolayers serve as model systems in which interfacial structure is governed not only by hydrogen bonding but also by headgroup chemistry, ion binding, and collective organization within the plane of the interface.

Surface-specific vibrational spectroscopy has shown that these features give rise to strong electrostatic responses in the interfacial water layer. In contrast to the neat air–water interface, where the response is dominated by intrinsic χ^(2)^, lipid interfaces typically exhibit substantial field-induced χ^(3)^ contributions arising from static surface charges.^264^ Phase-sensitive and heterodyne-detected SFG measurements demonstrate that the sign and magnitude of the OH-stretch response can be tuned by pH, ionic strength, and ion identity, reflecting changes in the interfacial electric field and water orientation.^277^ In particular, increasing surface charge density or adding electrolyte can induce a flip in the net orientation of interfacial water, indicating that the balance between intrinsic interfacial structure and field-driven bulk alignment is highly sensitive to surface charge and the subsequent screening.^82^ These observations establish that SFG at lipid interfaces primarily probes the response of water to interfacial fields rather than the lipid headgroups alone.

Beyond diffuse electrostatic screening, numerous studies show that ions interact directly with lipid headgroups, forming a chemically distinct Stern layer. Divalent cations such as Ca^2+^ strongly bind to negatively charged phospholipid monolayers, leading to measurable changes in both headgroup vibrational modes and interfacial water structure.^271^ These effects include partial neutralization of surface charge and reduction of the diffuse-layer contribution, behavior inconsistent with purely Gouy–Chapman screening. Even monovalent ions such as Na^+^ exhibit weaker but detectable association with headgroups, producing nonmonotonic changes in SFG spectra that reflect competition between diffuse screening and specific adsorption.^83,264^ At positively charged surfactant interfaces, polarizable anions such as SCN^–^ display strong surface affinity and adopt oriented configurations within the Stern layer.^278,279^ Recent work has shown that such ions can reorganize between Stern and diffuse layers as a function of chemical potential, forming multiple orientational populations and producing distinct water structures.^280^ These findings demonstrate that ion distributions at lipid interfaces cannot be described by diffuse electrostatics alone but instead require explicit treatment of ion-specific binding and interfacial complexation.

Ultrafast nonlinear spectroscopies reveal that these structural effects are accompanied by pronounced dynamical heterogeneity in interfacial hydration. Pump–probe SFG measurements show that water beneath lipid monolayers consists of multiple populations with distinct vibrational lifetimes, including a slow component associated with strongly bound or electrostatically constrained water and a faster component resembling bulk-like interfacial water.^281^ Time-resolved studies at surfactant interfaces similarly identify coexisting water species with different relaxation dynamics, indicating that hydration near charged headgroups is heterogeneous on molecular length scales (Figure 8).^270^ Two-dimensional heterodyne-detected SFG experiments further demonstrate that spectral diffusion at charged interfaces occurs on subpicosecond time scales but exhibits signatures of static inhomogeneity associated with Stern-layer and diffuse-layer environments.^260^ The development of multidimensional SFG techniques has further enabled direct correlation measurements at interfaces, providing a framework for linking spectral diffusion to interfacial structure.^282^ Additional ultrafast studies reveal strong frequency-dependent relaxation dynamics, consistent with a distribution of hydrogen-bond environments at aqueous interfaces.^172^ Complementary 2D-IR measurements of aqueous systems containing amphiphiles support this picture by revealing modest slowing of hydrogen-bond exchange dynamics in hydration shells near hydrophobic and charged groups.^283,284^ Although not surface-specific, these studies are consistent with SFG observations that interfacial water remains dynamically labile but experiences constraints imposed by electrostatics and confinement.

**Figure 8:**
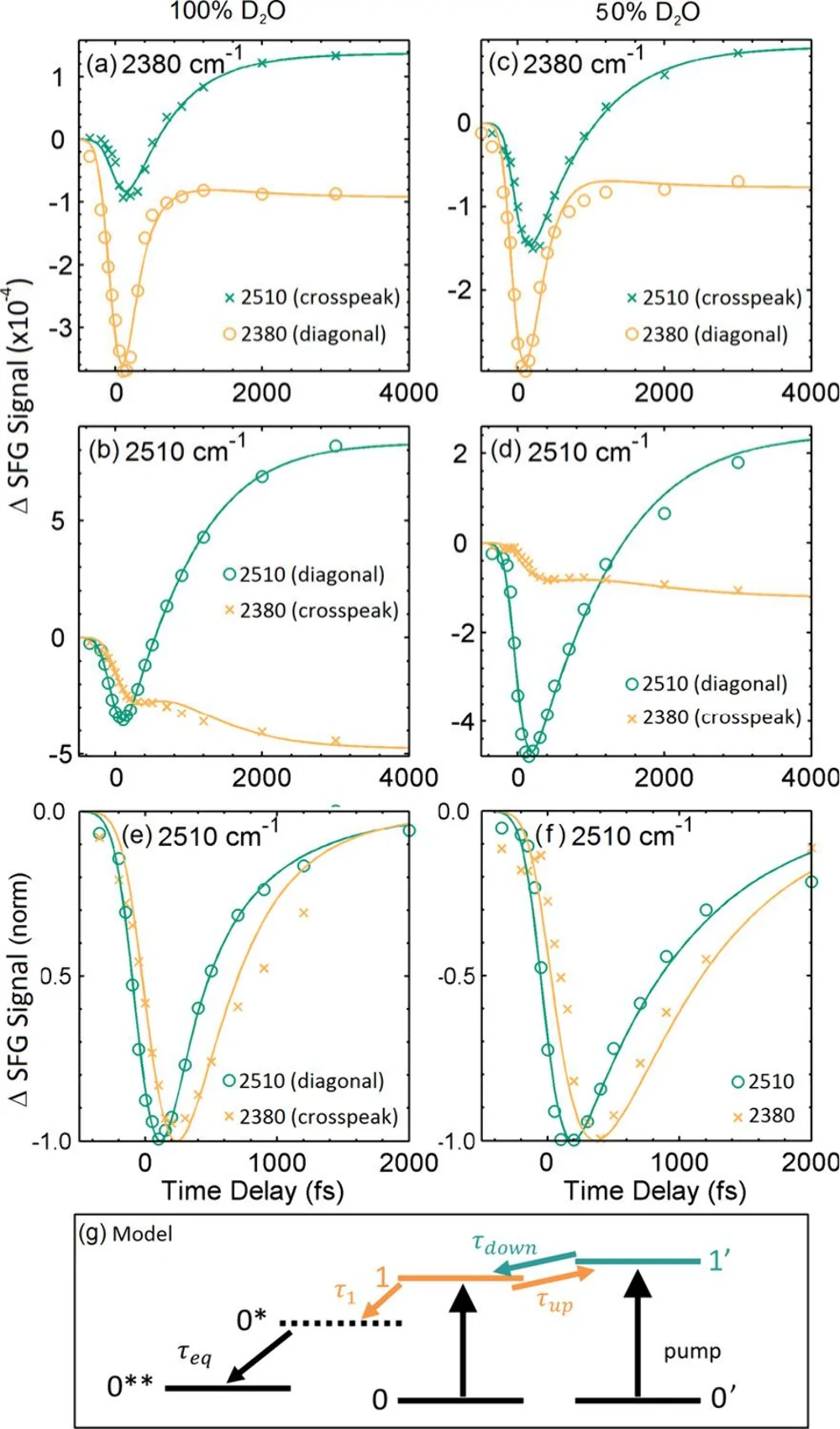
Time-resolved vibrational response of interfacial water at a charged surfactant/water interface. (a–d) Time-resolved SFG bleach signals measured for pure D_2_O and isotopically diluted D_2_O/H_2_O after excitation at 2380 or 2510 cm^–1^. Diagonal signals report relaxation at the same excitation and detection frequency, whereas cross-peak signals track spectral exchange between distinct OD stretch environments. The comparison between 100% D_2_O and 50% D_2_O separates collective intermolecular coupling from local hydrogen-bond dynamics. (e,f) Heat-corrected and normalized diagonal and cross-peak intensities following excitation at 2510 cm^–1^, showing the time scale for exchange between interfacial water populations. (g) Kinetic model used to describe vibrational relaxation, spectral exchange, and equilibration of the excited OD stretch populations. Reproduced with permission from Livingstone et al., *J. Am. Chem. Soc.* 2015, 137, 14912–14919.^270^ Copyright 2015 American Chemical Society.

Taken together, lipid monolayers and membranes constitute a regime in which specific ion effects, electric double-layer formation, and molecular organization converge. Static SFG measurements reveal strong field-driven orientation and ion-specific binding, while ultrafast spectroscopies show that hydration dynamics are heterogeneous yet remain rapid on subpicosecond time scales. These interfaces therefore provide a critical bridge between simple aqueous boundaries and complex biological systems, where fixed charges, ion correlations, and confined geometries collectively govern the interfacial structure and dynamics.

This connection becomes even more pronounced in biomolecular and polyelectrolyte systems, where higher charge densities and chemical heterogeneity further amplify ion-specific effects. For example, DNA-functionalized and polyelectrolyte-coated interfaces exhibit strong counterion condensation and restructuring of interfacial water, as revealed by heterodyne-detected SFG measurements.^285^ Two-dimensional SFG studies of DNA monolayers further demonstrate that molecular conformation and interfacial hydration are strongly coupled, providing direct structural insight into biomolecular interfaces.^286^ At liquid–liquid interfaces, charged macromolecules can assemble into highly ordered interfacial structures driven by ion-mediated interactions and electrostatic correlations.^287^ These results highlight that the interplay between ion adsorption, electrostatics, and molecular organization observed at lipid interfaces extends naturally to more complex biological and polyelectrolyte systems, where it governs both structure and dynamics at aqueous interfaces.

#### 3.3.5 Water Dynamics at Nucleic Acid Interfaces

At the surface of polyanionic DNA or RNA, the ion atmosphere should be considered the biomolecular analog to the EDL formed at soft and solid interfaces. It is instrumental in neutralizing the system charge on a length scale of 1–2 nm from the biomolecular surface.^288–290^ Spatial extent and ion concentration profiles are largely derived from Poisson–Boltzmann (PB) theory, which models counterions as point charges embedded in a structureless dielectric continuum. 2D-IR spectroscopy in the ∼ 1200 cm^–1^ fingerprint region provided surface-sensitive, noninvasive insight into hydration structures and water fluctuation timescales at the phosphate–water interface of DNA and RNA, thereby considerably refining this picture. The symmetric and asymmetric stretching modes of the phosphodiester backbone (ν_s_ ∼ 1085 cm^–1^ and ν_as_ ∼ 1220 cm^–1^, respectively) are highly sensitive to the local backbone conformation and the electrostatic environment around the phosphate (PO_2_)^−^ groups.^291^ Their frequency positions and lineshapes thus encode details of H-bond strength, their fluctuation dynamics, and interactions of the (PO_2_)^−^ group with ions of the electrolyte environment.

Ultrafast vibrational spectroscopy of DNA (AT)_23_ and RNA (AU)_23_ double-stranded model systems established ν_as_ (lifetime *T*_1_ ∼ 300 fs) as the primary reporter of phosphate–water interactions. The FFCF of the (PO_2_)^−^ stretching vibrations was characterized by 2D-IR spectroscopy,^292–294^ revealing a biexponential decay with a fast (300 fs) and a slower, quasi-static component (>10 ps), reflecting librational motions of the water shell and inhomogeneous disorder, respectively. In analogy to the observation at other interfaces, water motions at the surface of DNA experience only a moderate slowdown (50–100 fs → 300 fs) of the librational component. Compared with B-form DNA, the hydration shell of A-form RNA shows a higher degree of heterogeneity but is otherwise characterized by the same biexponential FFCF.^295^ The interaction of Mg^2+^ ions with tRNA and dsRNA was quantified via the sensitivity of ν_as_ to the local electrostatic environment. Contact ion pair (CIP) formation of Mg^2+^ and (PO_2_)^−^ groups was quantified via a distinct, blue-shifted vibrational band at ∼ 1250–1280 cm^–1^.^296–298^ Analysis of the 2D-line shape suggests that the amplitudes of the hydration water fluctuations are suppressed in the strong Coulomb field of the CIP geometries, directly impacting the vibrational lifetime of the (PO_2_)^−^ group (300 fs → 700 fs).

Interfacial electric fields at the DNA surface were quantified utilizing the vibrational Stark effect.^299^ The asymmetric (PO_2_)^−^ stretching vibration exhibits a Stark tuning rate on the order of −0.5 cm^–1^/(MV/cm), suggesting mean electric fields on the order of 60–80 MV/cm from the comparison of the frequency positions at varying hydration levels.^291,292^ Interestingly, such fields arise locally from the first 2–3 hydration layers of structured H_2_O molecules around the (PO_2_)^−^ groups, while field contributions from more distant H_2_O molecules or solvated ions are efficiently screened. Analysis of the 2D-IR line shape further provides access to field fluctuation amplitudes and timescales, with large-amplitude fluctuations (about 25 MV/cm) arising predominantly from direct coupling to librational H_2_O motions.

The molecular-level insight from 2D-IR spectroscopy into ion binding geometries and water fluctuation timescales at the biomolecular surface of DNA and RNA reveals inherent limitations of PB theory for such systems: (1) PB theory does not resolve the speciation of ion binding geometries at the biomolecular surface. While 2D-IR spectroscopy shows substantial CIP formation in tRNA and dsRNA, PB theory predicts ionic interactions only via diffuse outer-sphere binding. (2) PB theory neglects dispersion interactions and short-range Pauli repulsion between the ions and (PO_2_)^−^ functional groups that determine the details of ion binding geometries and the spectroscopic observables. (3) PB theory omits the molecular structure and ordering within the first few solvation shells, neglecting H-bond directionality, polarization, and charge-transfer contributions. As a consequence, the electrostatic forces at the biomolecular interface are qualitatively different. (4) PB theory inherently assumes a uniform dielectric constant *ε*, but the interfacial properties were found to be strongly dependent on water orientation, ion identity, and the (partial) dehydration of ions.

### 3.4 Physical Reactivity and Structural Transitions at Soft Interfaces

In addition to shaping the equilibrium interfacial structure, dissolved ions and temperature can drive structural transitions at soft aqueous interfaces by perturbing hydration, electrostatics, and molecular packing. These perturbations may appear as phase changes, conformational rearrangements, or shifts in interfacial organization, particularly in lipid and biomolecular assemblies, where water structure and molecular order are tightly coupled. Surface-specific nonlinear vibrational spectroscopies are especially powerful in this context because they directly report on changes in hydrogen bonding, orientational order, and field-induced responses within the interfacial region. In this section, we consider how salt, ion identity, and temperature trigger structural transitions at soft interfaces, with an emphasis on the molecular-level insight provided by static, time-resolved, and multidimensional vibrational measurements.

#### 3.4.1 Ion-Specific Control of Interfacial Water Structure and Phase Behavior

Salt and ions modify interfacial structure through three coupled mechanisms: screening of surface charge, ion-specific perturbation of the hydrogen-bond network, and direct adsorption or speciation at the interface. The relative importance of these contributions depends on whether the interface is neutral, zwitterionic, or charged, and on ion identity rather than concentration alone; this Hofmeister-type ion dependence underlies the diverse structural responses observed across lipid and aqueous systems.^247,300,301^ Mixed, biologically relevant lipid compositions reveal that charge- and dipole-driven water alignment are additive but distinct. Studies on mixtures of zwitterionic and anionic lipids show that net interfacial water orientation can be decomposed into a charge-driven component strongly modulated by ionic strength (and thus Debye length) and a dipolar component from zwitterionic headgroups that is essentially insensitive to salt and governed instead by local headgroup organization.^302^

Ion-specific effects on zwitterionic lipid phase behavior follow Hofmeister ordering. Surface-pressure isotherms and structural data for DPPC monolayers over different Hofmeister anions show that chaotropic anions such as I^–^, ClO_4_^–^, and SCN^–^ preferentially expand the liquid-expanded phase while leaving the liquid-condensed packing and chain tilt largely unchanged.^300^ This selectivity points to anion partitioning driven mainly by hydrophobic interactions with disordered acyl chains and local hydration, rather than by direct headgroup binding, and makes the liquid-expanded/liquid-condensed phase transition a sensitive reporter of ion-specific adsorption.^300^

Neutral glycolipid interfaces provide a striking case in which salt induces effective charging through ion capture. HD-SFG measurements of Ohmline, a formally neutral lactose-bearing glycolipid, show that increasing KCl strongly attenuates and eventually reverses the OH stretch response, while the CH region and monolayer packing remain largely unchanged.^303^ χ^(2)^/χ^(3)^ decomposition combined with Gouy–Chapman–Stern modeling gives a surface charge density of ≈0.026 C m^–2^ at 150 mM KCl, equivalent to roughly one trapped cation per ∼ 16 glycolipids. The differential line shape is consistent with a diffuse-layer contribution, indicating that the spectral changes arise from an emergent EDL caused by cation capture rather than headgroup deprotonation or monolayer disruption.^303^ The same measurements reveal that the EDL of Ohmline is mainly anion-specific. With the cation fixed, larger, more hydrophobic anions such as SCN^–^ and ClO_4_^–^ produce more positive OH amplitudes, whereas smaller, strongly hydrated anions such as Cl^–^ and Br^–^ enhance the negative EDL contribution. This trend is consistent with larger anions approaching the interface and reducing cation–anion separation, while smaller anions remain farther away and strengthen the diffuse layer field. By contrast, varying the cation at a fixed anion produces little spectral change at 150 mM, indicating robust monovalent cation capture and anion-dominated ion specificity.

Time-resolved SFG provides dynamical insight into how charge and ion identity affect interfacial water at lipid interfaces. Comparing negatively charged and positively charged monolayers with zwitterionic systems, time-resolved OH-stretch measurements show that the vibrational lifetime and spectral diffusion are interface-specific and report on hydrogen-bond heterogeneity that is not accessible in static spectra.^81^ Water at negatively charged monolayers exhibits longer-lived, more strongly hydrogen-bonded populations than at positively charged interfaces, such as cationic lipid monolayers, where water dynamics approach bulk-like behavior.^273^ This charge-sign asymmetry in dynamics mirrors the asymmetry in static water orientation and highlights that ion adsorption modifies both the equilibrium structure and the picosecond reorganization kinetics. Previous time-resolved SFG (TR-SFG) measurements on phosphocholine and inverse-phosphocholine monolayers demonstrate that subpicosecond OH vibrational dynamics are insensitive to the sign of the water dipole orientation, and instead reflect the local hydrogen-bond strength and topology.^304^ This result is important for interpreting salt-dependent SFG spectra: changes in time-domain observables upon salt addition should be attributed primarily to modifications of local bonding environments, rather than to simple reorientation of the interfacial dipole layer.

#### 3.4.2 Temperature-Driven Interfacial Structure, Dynamics, and Phase Transitions

Temperature modifies the interfacial water structure and lipid organization through two physically distinct mechanisms: weakening of the bulk hydrogen-bond network and entropy-driven disorder in the acyl chain region. These mechanisms are not independent; lipid chain disorder directly modulates headgroup geometry and hydration, while hydrogen bond restructuring in the interfacial water layer feeds back into lipid packing and phase stability. Disentangling these coupled effects requires measurements that are simultaneously interface-specific and sensitive to either the structure or dynamics.

At the bare air–water interface, SFG spectroscopy combined with many-body MD simulations reveals that the hydrogen-bonded OH band near 3300 cm^–1^ decreases in amplitude with increasing temperature, while the free OH feature at 3700 cm^–1^ is essentially unchanged.^305^ Crucially, this variation is not caused by a change in molecular orientation of interfacial water, but is fully accounted for by the temperature dependence of the bulk optical response of water. The molecular organization of the air–water interface is thus surprisingly insensitive to temperature; what varies is the effective interfacial thickness, i.e., surface roughness. Polarization-dependent SFG calculations using the MB-pol potential confirm these observations and further show that the low-frequency shoulder of the free OH feature near 3600 cm^–1^ arises from intermolecular couplings between singly and doubly hydrogen-bonded molecules at the surface, both of which are thermally populated.^306^

The vibrational energy-relaxation dynamics at the air–water interface exhibit a clear, mode-specific temperature dependence. Time-resolved SFG on isotopically diluted D_2_O reveals that the relaxation time scale of the hydrogen-bonded OD stretch is temperature-independent, while the lifetime of the free OD stretch decreases with increasing temperature (Figure 9).^307^ Simulations indicate that both intramolecular vibrational redistribution and rotational reorientation contribute to free OD relaxation, and that temperature modulates these two mechanisms differently.^307^ This mode-selective temperature sensitivity provides a useful observable for tracking changes in the free-to-hydrogen-bonded OD population ratio as a function of interfacial perturbation, including those induced by lipid deposition.

**Figure 9:**
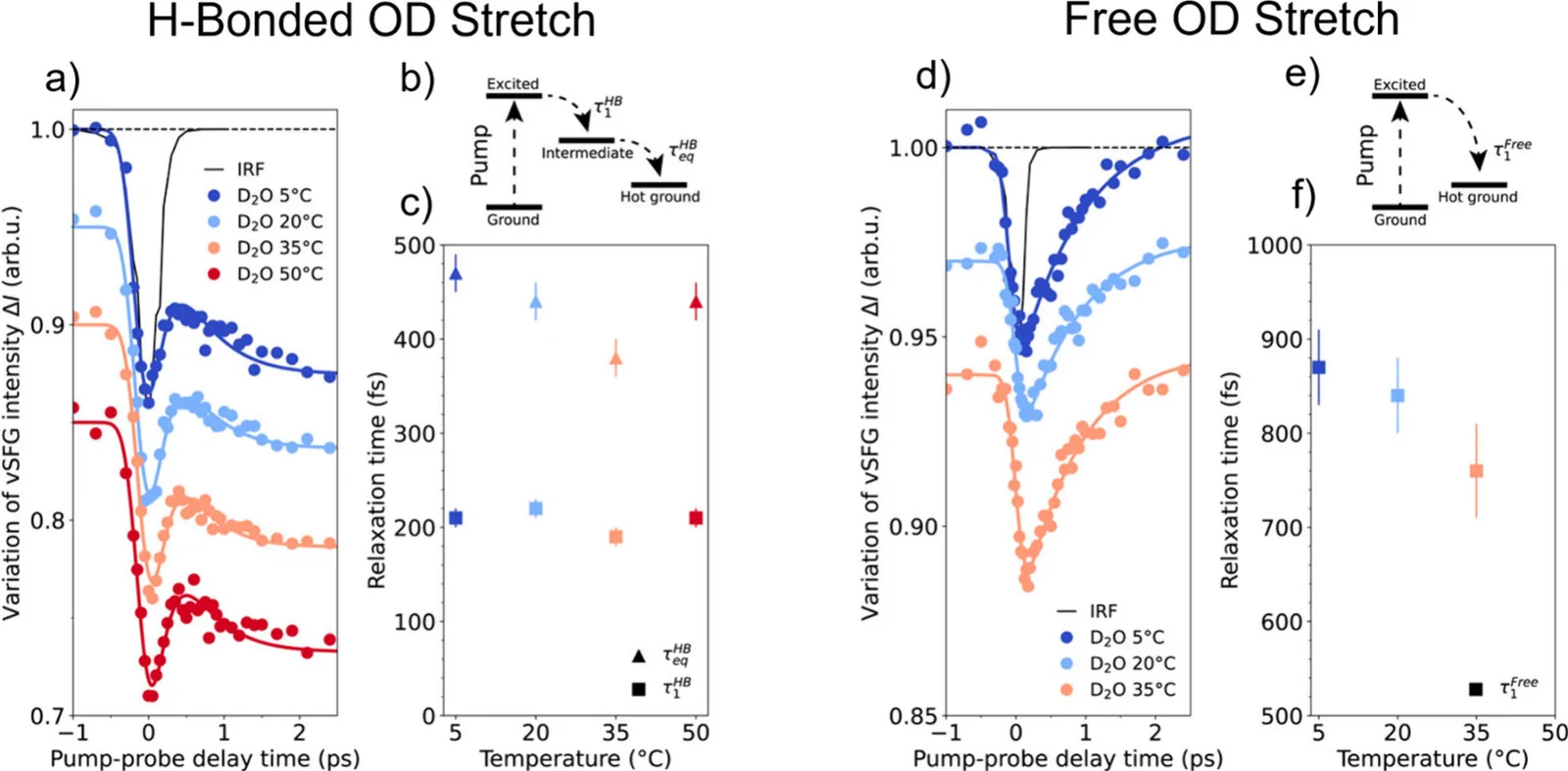
Temperature-dependent vibrational energy relaxation of hydrogen-bonded and free OD groups at the air/D_2_O interface. (a) Time-resolved vSFG intensity changes in the hydrogen-bonded OD stretch region at different temperatures. The rapid bleach recovery reflects the population relaxation of hydrogen-bonded OD groups, while the slower offset at later delay times arises from the heated ground-state response. (b) A four-level kinetic model is used to describe relaxation of hydrogen-bonded OD groups through an intermediate nonthermal state before equilibration with the hot ground state. (c) Extracted lifetimes for the first excited hydrogen-bonded OD state and the intermediate state as a function of temperature, showing weak temperature dependence over the measured range. (d) Time-resolved vSFG intensity changes in the free OD stretch region at different temperatures. Compared with hydrogen-bonded OD groups, the free OD bleach recovers more slowly and exhibits a clearer temperature dependence. (e) A three-level kinetic model is used to describe the relaxation of the free OD stretch, in which vibrational energy must first couple into hydrogen-bonded OD modes before dissipating into lower-frequency modes. (f) Extracted free OD excited state lifetimes as a function of temperature, showing faster relaxation at higher temperatures. Adapted from Greco et al., *J. Chem. Phys.* 2024, 161, 174706,^307^ under the terms of the Creative Commons CC BY 4.0 license. Copyright 2024 The Authors.

The ice–air interface provides the most unambiguous test system for temperature-driven hydrogen-bond structural transitions, since the ice-to-water transition is thermodynamically sharp and well-defined, and SFG directly probes dangling OH groups, quasi-liquid layer formation, hydrogen-bond restructuring, and proton ordering at crystalline ice surfaces.^308^ SFG and TR-SFG studies on single-crystal hexagonal ice (ice Ih) have revealed that the bonded-OH stretch intensity decreases by roughly a factor of 5 upon warming from −90 °C to near the bulk melting point, accompanied by a shift of the hydrogen-bonded OH band to higher frequencies near −16 °C.^309^ This spectral evolution directly reflects progressive weakening of interfacial hydrogen bonds as the quasi-liquid (QL) layer develops at the ice surface, a disordered premelt layer whose thickness increases continuously with temperature.^309^ Three distinct QL states are distinguishable by combining SFG with laser confocal microscopy: a disordered surface layer present at all temperatures, QL droplets that nucleate kinetically at intermediate temperatures, and a QL film appearing close to 0 °C.^309^

Recent first-principles machine-learning simulations provide a microscopic reference for interpreting these ice SFG spectra below the premelting regime. Berrens and co-workers showed that SFG can distinguish proton-ordered and proton-disordered ice surfaces as well as different ice facets, with the response dominated mainly by the top surface bilayer and smaller contributions from the second bilayer. These results support a proton-ordered ice Ih surface below premelting and clarify how temperature-dependent spectral changes should be interpreted relative to the underlying facet structure, proton ordering, and local hydrogen bond topology.^310^

Time-resolved SFG on ice Ih reveals that the two principal OH populations at the ice surface respond to temperature in opposite ways.^311^ The hydrogen-bonded OH undergoes accelerated vibrational energy relaxation relative to liquid water, attributed to enhanced intermolecular coupling in the crystalline lattice, while the free OH relaxes more slowly than at the liquid–air interface, consistent with reduced rotational mobility of interfacial molecules in the crystal.^311^ Upon warming toward the QLL onset, the amplitude of the free-OH feature decreases as premelting screens the dangling bonds, and the vibrational lifetime of the bonded OH shortens continuously, tracking increased disordering of the interfacial hydrogen-bond network.^311^

The mode-specificity of temperature effects at the ice surface extends to the water bending mode near 1650 cm^–1^. SFG on the basal plane of single-crystal ice Ih shows that the bending-mode response is weakly temperature-dependent and exhibits a large line width, inconsistent with inhomogeneous broadening, pointing to ultrafast pure dephasing as the dominant line shape mechanism.^312^ This implies the bending region is less sensitive to progressive hydrogen-bond weakening upon warming, but makes it an excellent complementary probe of adsorbate–ice interactions and chemical differences between ice and liquid water surfaces.^312^ However, recent depth-resolved SFG/difference-frequency generation (DFG) measurements at the air–water interface show that bulk-like quadrupolar contributions can dominate the bending response, so structural assignments require isolating the interfacial dipolar response.^313^

At lipid interfaces, the SFG CH stretch region provides a direct thermometer for acyl chain order. Liu and Conboy used the symmetric methyl stretch at 2875 cm^–1^ to directly measure the gel-to-fluid phase transition temperature of single supported planar lipid bilayers (DPPC, DHPC, DSPC), demonstrating that the SFG intensity passes through a maximum at the transition temperature due to broken bilayer symmetry.^314^ Above the transition, the bilayer symmetry is restored as both leaflets disorder, reducing the CH_3_ SFG signal; below the transition, the gel phase constrains chain tilt and orientation, giving a strong, well-defined CH_3_ response.^314^ This established SFG as a direct structural probe of lipid phase behavior in single monolayers and bilayers with sensitivity not accessible to bulk calorimetric methods on the same material.

The broader landscape of temperature-induced interfacial structural transitions, including gel-to-liquid-crystalline transitions, phase coexistence, and domain formation, is well captured by simultaneous temperature-dependent SFG in the CH and OH regions.^315–317^ As temperature increases through the main transition, the CH_3_ symmetric stretch amplitude decreases and broadens as chain order is lost, while the OH stretch region shows a concomitant reduction in amplitude consistent with loss of headgroup-induced water alignment.^315^ Below the transition, tightly packed acyl chains maintain a well-defined orientation distribution, so both CH and OH SFG responses are larger and spectrally narrower than those in the disordered fluid phase.^315^

2D-IR spectroscopy has provided complementary molecular evidence that temperature-driven transitions at lipid interfaces involve heterogeneous domain structures rather than homogeneous order–disorder conversion. Volkov and Righini used 2D-IR on binary sphingomyelin/phospholipid liposomes to show that off-diagonal cross-peaks between amide I and carbonyl modes indicate intermolecular coupling and lipid domain segregation at temperatures near the coexistence regime.^318^ These cross-peaks are temperature-dependent, decreasing as the system enters the fully fluid phase where domains dissolve and lipid mixing becomes homogeneous. 2D-IR and temperature-dependent infrared absorption measurements on lipid nanodiscs and vesicles using ester carbonyl probes further show that spectral diffusion accelerates and carbonyl inhomogeneity decreases as chain disorder increases above the transition, consistent with a reduction in static heterogeneity of carbonyl environments in the fluid phase.

### 3.5 Chemical Reactivity at Soft Interfaces

Beyond structural and dynamical effects, soft aqueous interfaces can alter chemical reactivity by reshaping protonation equilibria, solvation structure, and molecular organization within the interfacial region. These influences are highly system-specific and reflect local hydration, ion distributions, and interfacial geometry rather than uniform interfacial fields. Nonlinear vibrational spectroscopies are particularly well suited to probe such behavior, as they directly report on interfacial species, hydrogen-bond structure, and electric field contributions with molecular specificity and ultrafast time resolution. In this section, we consider how soft interfaces modify chemical reactivity, beginning with acid–base chemistry and proton transfer, and then moving to organic and inorganic reactions at aqueous interfaces.

#### 3.5.1 Acid–Base Chemistry

Aqueous acid–base processes are among the most fundamental reactions in chemistry, yet the question of how protonation states, proton-transfer mechanisms, and EDL dynamics are modified at soft aqueous interfaces has only recently become accessible to experiment. Ultrafast nonlinear vibrational spectroscopies, in particular, HD-SFG, time-resolved SFG, and 2D-IR, have provided direct, molecularly resolved insight into proton surface activity, the structure of interfacial hydronium, and the coupling between protonation and interfacial water orientation.

Early SFG measurements on acidic and basic aqueous solutions established that hydronium ions preferentially partition to the air–water interface. Phase-resolved studies of HCl, HI, and NaOH solutions showed that interfacial H_3_O^+^ generates a net positive electric field that enhances the orientation of interfacial water molecules, leading to an increase in the amplitude of the hydrogen-bonded OH stretch region and a modification of the free-OH response.^245,319^ In systems containing more surface-active anions such as iodide, coadsorption leads to the formation of a compact interfacial charge distribution, often described as a double-layer-like structure, which further alters the interfacial water response.^245,258^ In contrast, hydroxide showed only weak surface propensity, only modest changes in the liquid-like band, and the disappearance of the ice-like feature as the primary spectroscopic signature.^245^ These foundational experiments established that the SFG response of acidic electrolyte solutions reflects not only ion distributions but also the field-induced reorientation of interfacial water molecules.

Subsequent SFG work significantly sharpened the quantitative picture of proton surface activity. Das et al. used surface-sensitive SFG to determine that detectable perturbation of the D_2_O–air interface begins at a bulk D^+^ concentration as low as 2.7 ± 0.2 mM, whereas several hundred mM of OD^–^ is required to produce a comparable effect, making the hydrated proton orders of magnitude more surface-active than hydroxide.^320^ Complementary SFG and ab initio molecular dynamics (AIMD) studies associated interfacial hydronium with disruption of dangling OH populations and broad proton-continuum absorption in the 1600–3000 cm^–1^ region.^321^ However, phase-sensitive SFG measurements by Chiang and co-workers showed that, at low to moderate acid concentrations, hydronium adsorption can be quantified by separating the bonded-interface-layer and electrical-double-layer responses and using the χ^(3)^ contribution to extract the hydronium surface density.^322^ A subsequent neural-network-assisted AIMD study showed that H_3_O^+^ at the interface causes two distinct changes in phase-resolved SFG spectra: a low-frequency tail arising from H_3_O^+^ and its first hydration shell, and an enhanced hydrogen-bonded band reflecting ion-induced polarization of deeper water layers.^319^ Ultimately, these studies show that H^+^/D^+^ becomes enriched at the interface relative to the bulk and strongly perturbs the topmost hydrogen-bond network, whereas OH^–^/OD^–^ remains much less surface-active and produces only weak interfacial restructuring under comparable conditions.

The coupling between the acid–base protonation state and interfacial EDL formation has been directly observed using HD-SFG. Singh et al. performed the first 2D heterodyne-detected vibrational SFG (2D HD-VSFG) experiments at a positively charged surfactant/water boundary and revealed inhomogeneous broadening and spectral diffusion in the OH stretch region, with diagonal elongation decaying within approximately 300 fs, indicating that hydrogen-bond rearrangement at charged interfaces differs fundamentally from bulk water.^260^ Further steady-state and time-resolved HD-VSFG measurements showed that the dynamics of OH spectral diffusion are sensitive to counterion identity, with fluoride producing markedly different relaxation behavior from chloride and bromide.^323^

#### 3.5.2 Microdroplet Reactivity and the Role of Interfacial Electric Fields

Reactions at the aqueous aerosol-cloud droplet interface are central to atmospheric processes, including ozone degradation, sulfate formation, and organic aerosol aging.^324^ At the same time, laboratory observations of accelerated reaction rates and unconventional product formation in aqueous microdroplets, emulsions, and sprays have shown that interfaces catalyze reactions even without added reagents or external catalysts.^325,326^ A recurring hypothesis in this literature is that large or long-lived electric fields intrinsic to the air–water interface provide the electrostatic driving force for stabilizing transition states, polarizing bonds, and enabling otherwise thermodynamically unfavorable redox chemistry.^327,328^

Many interpretations of interfacial reactivity invoke unusually strong or long-lived electric fields at the neat air–water interface. Shirley et al. tested this hypothesis with vibrational SFG by applying established OH stretch frequency-to-field mappings to interfacial water and comparing the resulting field distributions with bulk water.^211^ The measured imaginary χ^(2)^ spectra showed none of the expected signatures of strong static fields, such as red-shifted continua, large frequency shifts, or enhanced anharmonicity; instead, the bonded OH region gave identical effective fields of 2.1 × 10^2^ MV/cm at the interface and in bulk water. Time-resolved and multidimensional spectroscopies likewise show comparable surface and bulk spectral diffusion timescales, while longer interfacial hydrogen-bond residence times argue against stabilization of long-lived charge-separated configurations (Figure 10).^116,198,203,268^ These constraints are directly relevant to microdroplet chemistry, where initial claims of spontaneous H_2_O_2_ or hydroxyl radical generation from pure water have been challenged. Subsequent studies show that H_2_O_2_ formation depends on dissolved oxygen and water–solid contact rather than pristine air–water interfaces, and is absent under rigorously deoxygenated conditions.^329–331^ Mass spectrometric signals previously assigned to hydroxyl radicals have also been reinterpreted as ammonia-derived artifacts, with deliberate hydroxyl radical generation producing extensive oxidative damage not observed in unactivated droplets.^332,333^ Thermodynamic and mechanistic reassessments further show that electron transfer requires significant droplet charge near the Rayleigh limit and that many accelerated reactions can be explained by surface adsorption, preorganization, evaporative concentration, and altered solvation without invoking anomalous fields.^327,334^ Ultimately, these results indicate that microdroplet reactivity is better explained by droplet charge, dissolved oxygen, water–solid contact, and evaporative concentration than by intrinsic electric fields at the neat air–water interface.^211,327^

**Figure 10:**
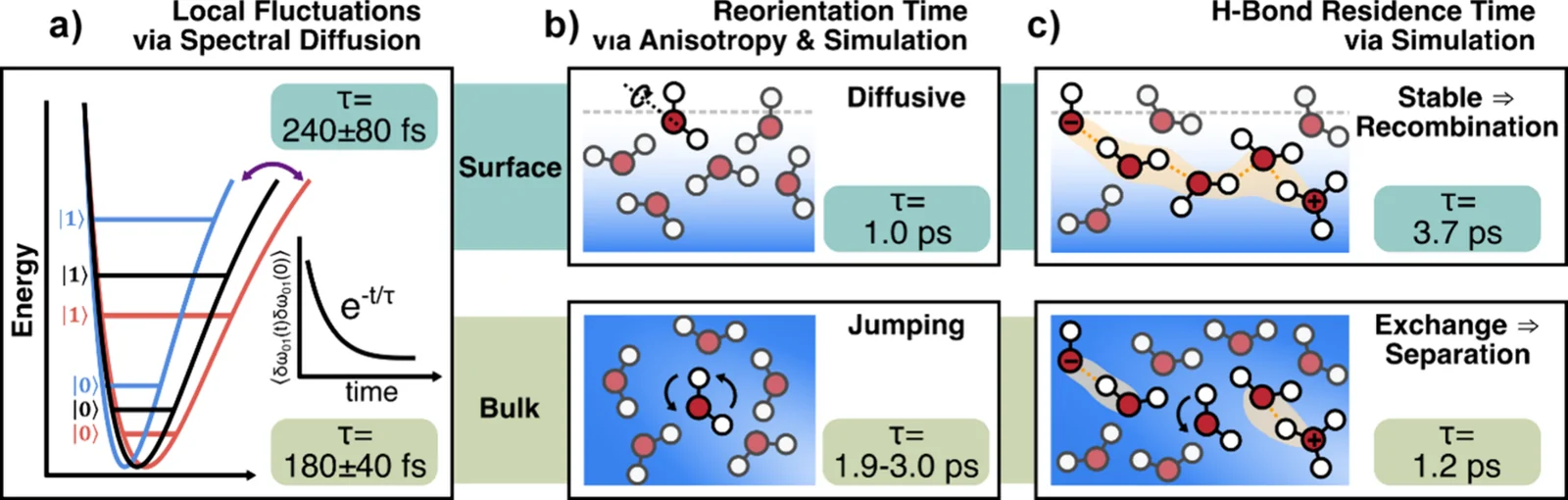
Comparison of bulk and interfacial OH stretch dynamics relevant to proposed anomalous electric fields at the air/water interface. (a) Schematic comparison of spectral diffusion in bulk and interfacial water. Similar spectral diffusion time scales indicate that the OH stretch at the surface does not sample an unusually slow or strongly perturbed potential energy landscape. (b) Comparison of water reorientation in bulk and at the interface. Bulk water reorients primarily through hydrogen bond exchange and angular jumps, whereas interfacial water exhibits distinct orientational dynamics associated with restricted hydrogen bonding and the free OH population. (c) Comparison of hydrogen bond residence times. Interfacial hydrogen bonds are longer-lived than those in bulk water, which is inconsistent with a picture of unusually frequent surface charge separation events. Reproduced with permission from Shirley et al., *J. Am. Chem. Soc.* 2025, 147, 46163–46173,^211^ under the terms of the Creative Commons CC BY 4.0 license. Copyright 2025 The Authors.

A central challenge moving forward is to connect interface-specific vibrational observables directly to reactive intermediates, free energy barriers, and nonequilibrium chemical pathways. A particularly important distinction is whether an interface changes the intrinsic reaction mechanism, for example, by stabilizing a new transition state, opening a different intermediate pathway, or enabling charge-transfer chemistry, or whether it instead changes the local reaction environment by concentrating reactants, imposing preferred orientations, modifying solvation, or altering transport. These scenarios can produce similar apparent rate enhancements but require fundamentally different molecular interpretations. Extending phase-resolved, time-resolved, and multidimensional spectroscopies to more complex reactive interfaces, while integrating simulation and selective vibrational reporters, will therefore be essential for separating true mechanistic changes from interfacial preorganization and mass-transport effects. Resolving this distinction will be critical to building a predictive framework for interfacial chemistry in aerosols, microdroplets, membranes, and catalytic soft matter systems.

### 3.6 Conclusions and Outlook

Soft aqueous interfaces show that interfacial water structure, ion adsorption, and reactivity are governed by coupled molecular effects rather than by a single universal principle. Across air/water, graphene/water, oil/water, lipid, glycolipid, biomolecular, and reactive interfaces, nonlinear vibrational spectroscopies show that hydrogen bonding, local chemistry, ion specificity, electrostatics, and molecular organization jointly determine the observed response. Weakly interacting hydrophobic interfaces can preserve an air/water-like hydrogen-bond structure, whereas charged, surfactant-covered, or molecularly coated interfaces produce stronger field-induced signals, specific ion binding, and heterogeneous hydration environments. Classical mean-field descriptions are therefore useful only under specific limits. EDL models apply best to charged or strongly binding interfaces where fixed charges and ion adsorption generate robust interfacial fields. Neutral or weakly interacting boundaries are more often characterized by short-range hydration effects, subsurface ion stratification, or ion accumulation without major water restructuring. Likewise, salt- and temperature-driven transitions are controlled not only by changes in the mean electrostatic field but also by ion capture, hydration-shell restructuring, headgroup organization, chain disorder, and mesoscale phase behavior. Chemical reactivity follows the same logic. Rate enhancements and altered pathways at aerosols, microdroplets, lipid interfaces, and organized soft matter generally reflect changes in protonation, solvation, reactant concentration, molecular orientation, transport, and interfacial organization. Current spectroscopic evidence does not support the notion of anomalously large intrinsic electric fields at the neat air/water interface as a universal driver of interfacial chemistry; instead, soft interfaces reshape reaction pathways through local hydration and molecular preorganization.

Moving forward, the central challenge is to connect nonlinear vibrational observables directly to microscopic mechanisms. This will require separating intrinsic χ^(2)^ responses from field-induced χ^(3)^ contributions, distinguishing true mechanistic changes from concentration or transport effects, and extending phase-resolved, time-resolved, and multidimensional measurements to more complex soft interfaces. Combining heterodyne-detected SFG, multidimensional spectroscopy, isotope-selective probes, controlled salt and temperature perturbations, and molecular simulations should enable a more predictive framework that links ion identity, hydration dynamics, interfacial organization, and chemical reactivity in soft aqueous environments.

## 4 Solid–Liquid Interfaces and Heterogeneous Systems

### 4.1 Introduction and Overview

In this subsection, we focus on ultrafast vibrational spectroscopy studies interrogating the interfaces between solids and aqueous electrolyte solutions. Throughout this section, it is worth keeping in mind that SFG is not the only vibrational window on these processes. Where a vibrational reporter can be tethered to the surface at submonolayer coverage, surface-enhanced 2D-IR spectroscopy provides information that static SFG cannot: it permits extraction of the reporter frequency–frequency correlation function, which is sensitive to local electric-field fluctuations and hydrogen-bond dynamics. Mapping these observables onto a distance-resolved water response requires knowledge of the probe geometry and its coupling to the environment. Nitrile-based probes such as 4-mercaptobenzonitrile self-assembled on gold, discussed further in Sections 4.2.3 and 4.5.2, have been used in exactly this way to follow interfacial field dynamics under potential control, and analogous approaches have been applied to oxide and metal surfaces. The two techniques are therefore complementary rather than competing: SFG can report directly on interfacial water, including its dynamics in time-resolved and two-dimensional implementations, whereas probe-based surface 2D-IR reports local dynamics through an attached vibrational label. Reconciling the picture obtained from these two classes of experiments is one of the more concrete open problems in the field, and we return to it in Section 6.

From an experimental viewpoint, a prerequisite for such experiments is optical accessibility of these interfaces with short laser pulses, which naturally led to a focus on optically transparent materials: to obtain interfacial vibrational information, infrared pulses resonant with vibrational transitions need to reach the interface, which is challenging when propagating these pulses through highly absorbing water. Hence, infrared-transparent solids are often used. The solid interfaces studied have traditionally been grouped into three major classes: oxides, other minerals, and metals; the last require different optical access strategies.^335–338^ With increasing interest in two-dimensional materials, such as graphene and hexagonal boron nitride,^225,265^ vibrational spectroscopies have been used to elucidate their vibrational structures at their interfaces. Yet, these materials elude traditional classification as either solid materials or soft matter, making it difficult to draw a clear distinction. For experimental feasibility, these two-dimensional materials are often supported by solid substrates,^339^ and conducting two-dimensional materials provide a unique platform for studying electrochemical interfaces.^223,224^

Among the first class of materials, solid oxides,^340^ interfaces with SiO_2_ are arguably the most intensely studied solid, as SiO_2_ is available in the form of crystalline quartz,^341^ more amorphous silica,^342,343^ or in nanoporous form,^344^ allowing for a wide range of spectroscopic characterizations with, e.g., SFG and 2D-IR spectroscopy. Almost equally well studied are alumina/sapphire interfaces with water,^345^ which can be readily interrogated by SFG. Due to their potential application for water splitting, TiO_2_–water interfaces—irrespective of the exact crystalline form of TiO_2_, be it rutile,^346^ anatase,^347^ or in the form of nanocrystalline films^348,349^ have shifted into the focus in recent years. Conversely, information on interfaces with other oxides, such as MgO,^350^ Li_2_O,^351^ Cr_2_O_3_,^352^ SrTiO_3_,^353^ and mica^354^ is to date scarce. For the oxides, the majority of the work has employed SFG spectroscopy, which has been partly summarized in earlier reviews.^335–337,340,355,356^ Investigations using 2D-IR have remained restricted to materials that exhibit a porous structure, silica^357–359^ and zeolites.^359^ Common to all these oxides is that they can acquire a surface charge upon contacting aqueous solutions, with distinct charging mechanisms. At the interface, oxides are partially terminated by OH groups (Figure 11), which can be more or less acidic, ranging from weak Brønsted acids, e.g., silica^360,361^ to very strong acids in zeolite.^359^ This charging mechanism, the dissociation of surface OH groups, sets oxides apart from other minerals and is the reason why oxides are often considered as a separate class of minerals.

**Figure 11:**
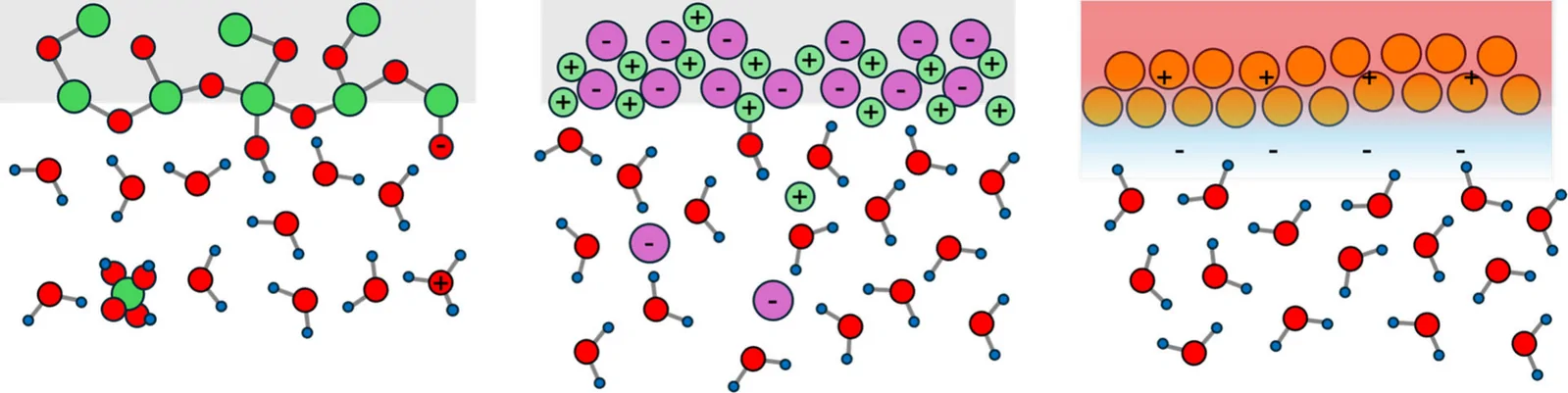
Schematic illustration of the three main categories of solid–liquid interfaces. For solid oxides in contact with water (left), surface hydroxyl groups are critical to their hydrophilicity; the acidity of hydroxyls determines surface charge, and molecular fragments can dissolve. For ionic minerals (center), constituent ions can dissolve separately, which is pivotal to charging, and ions such as hydroxyls can strongly adsorb to the interface. For metals in contact with water (right), the high polarizability critically determines interaction with aqueous electrolytes and results in marked optical responses.

With the other mineral–electrolyte interfaces, fluorite (CaF_2_) is certainly the most intensively studied solid^337,362,363^ using nonlinear vibrational spectroscopies, as it is transparent for both beams of an SFG experiment. Remarkably, interfaces with the equally transparent and somewhat more soluble BaF_2_ are hardly studied. Less commonly studied with SFG are calcium salts with low solubility, such as birefringent calcite (CaCO_3_)^364^ or calcium oxalate.^365^ CaSO_4_-based minerals have been studied with both SFG^366,367^ and 2D-IR^368^ experiments. In contact with water, all these minerals acquire a surface charge. The charging of these minerals can be only partially explained by a mechanism similar to the oxides discussed above: water molecules adsorbed to the interface dissociate into adsorbed OH^–^ ^369^ and release H^+^ (Figure 11). The major mechanism, however, is ascribed to differences in the solvation free energies of the cations and the anions, resulting in a nonstoichiometric dissolution (Figure 11) of the ions and a concomitant charging in the near-surface region.^369^ As this charging mechanism is characteristic of ionic minerals—as opposed to the more covalent networks of oxides—these minerals are often discussed separately from oxides.

While the naturally acquired charge on oxides and other minerals in contact with water provides an ideal platform for studying the effect of surface charging on aqueous electrolytes, the discussed solids are almost exclusively insulating materials (i.e., their optical band gaps are large enough to make them transparent to the optical beams). Yet, to study electrochemical phenomena rather than the naturally occurring charging of solid–liquid interfaces, optical access to metal–liquid interfaces is required, allowing for systematic variation of surface charge via the application of external potentials. This optical access was realized using both 2D-IR and SFG. For SFG experiments, optical access is challenging because metals are not transparent to visible and infrared light and the liquid inherently absorbs infrared light at the frequencies of interest. Yet, this can be circumvented by using either thin liquid layers^338^ or thin electrodes,^370^ where the absorbing media are thin enough to provide sufficient optical transparency. Alternatively, different geometries can be employed in which the beams are propagated through the most transparent medium.^371^ Similarly, using thin electrodes and/or thin liquid layers in spectroelectrochemical cells has enabled 2D-IR experiments at metal–liquid electrolyte interfaces.^372–374^ However, the high polarizability of thin metal layers, which originates from highly mobile electrons, often leads to the generation of intense, so-called nonresonant signals within the metals (Figure 11), which can obscure the interfacial signals of interest.^375,376^ Nevertheless, structural and dynamical information from nonlinear spectroscopies has become available at the electrochemically relevant platinum–electrolyte^372,377–379^ and gold–electrolyte^373,377,380–382^ interfaces. Although less commonly studied, the interface of less noble electrodes such as silver,^383,384^ copper,^382,385^ and silicon^371,386^ has become spectroscopically accessible. As mentioned above, despite its categorization as a soft material in this Review, graphene’s atomic thickness, optical transparency, and electrical conductivity make it an ideal platform for studying electrochemical phenomena,^223,224^ and although some aspects of graphene electrodes have already been covered in the previous section (Section 3.2.1), we include a discussion of the electrochemical aspects of graphene interfaces below.

The above overview is not exhaustive and serves only as a representative snapshot of solid–liquid interfaces, illustrating both the breadth of systems studied and the diversity of research questions addressed. Several aspects have been previously discussed in earlier reviews, including the structure of water at solid interfaces,^254,262,335–337,387^ the distribution of ions at interfaces,^262,387^ or the dynamics at solid–liquid interfaces.^17,355,388^ In this section, we focus on the studies that have addressed the following phenomena relevant to electrolyte–solid interfaces: hydrophilicity and hydration of solid interfaces in contact with electrolytes, ion adsorption to and dissociation of ions from the solid, chemical reactions of the interfaces, and electric-field-induced phenomena, as these topics have witnessed considerable progress over the past decades.

### 4.2 Hydrophobicity, Hydrophilicity, and Hydration of Solid Interfaces

Nonlinear vibrational spectroscopies have been extensively used to study the interaction of solids with water, often via interrogation of the vibrational response of water. Here, we summarize findings regarding the interaction of water with solid oxides, other minerals, and metals.

#### 4.2.1 Oxides

The strong vibrational response of interfacial and hydrogen-bonded OH groups makes the OH stretching region a particularly sensitive probe of water–oxide interactions, which have therefore been examined in considerable detail for well-characterized systems such as silica and related oxides. Discussion of hydrophobicity and hydrophilicity is generally associated with the observed frequencies of the interfacial OH groups: red-shifted OH groups indicate hydrophilicity (strong hydrogen bonds), whereas blue-shifted OH stretching bands are often related to weakly interacting water molecules (hydrophobicity). Due to the charged nature of oxides, SFG spectral signatures originate from different depths. Often, electric field-induced signals, which are generated at the interface but can also originate hundreds of nanometers away from the surface in the liquid phase, dominate.^389,390^ In this subsection, we focus on what has been learned about the very first layer of water molecules that directly interact with the solid oxide, sometimes referred to as the binding interfacial layer (BIL, topmost ≈ one molecular layer).^389^ For fully hydroxylated quartz, water in the BIL has been reported to reside in a high-density layer where water molecules are highly ordered by an alternating lattice of in-plane and out-of-plane silanols.^389^ This organization gives rise to a strong SFG band near 3400 cm^–1^ from vertically oriented, strongly hydrogen-bonded “donor-to-solid” waters, and a weaker negative band below ≈ 3300 cm^–1^ from waters (and out-of-plane silanols) that accept hydrogen bonds from the surface (Figure 12).^389,391,392^ For amorphous silica, the same layering concept seems to apply, but water is less strongly bound, reflecting weaker, more heterogeneous interactions with a rougher, chemically mixed silanol/siloxane surface.^389^ Silanol groups apparently act as the primary hydrophilic sites, providing both hydrogen-bond donors and acceptors to the binding layer of the water network.^389,393^ The relative contribution of water molecules forming hydrogen bonds to silanol and silanolate groups obviously depends on the number density of H-bond acceptor sites that vary with pH.^393^ Generally, the oriented water molecules with rather strong hydrogen bonds underlie the intense 3200–3400 cm^–1^ interfacial water bands, and as such, these interfaces have usually been classified as hydrophilic interfaces.^394^ Nevertheless, vibrational SFG of silica in humid air shows a narrow band at ≈ 3750 cm^–1^ from isolated, non-hydrogen-bonded silanols that persists from 5 to 95% relative humidity, which may at first glance be inconsistent with the hydrophilic nature of silica, yet has been explained by water initially adsorbed in clusters and multilayers without fully hydrogen-bonding to all surface OH groups.^395^ With increasing humidity, the broad band at ≈3400 cm^–1^ grows due to strongly hydrogen-bonded adsorbed water, while the silanol band remains largely unperturbed, consistent with island-like water films coexisting with “free” silanols on an overall highly wettable (low contact angle) surface.^395^ These observations support a picture in which macroscopic hydrophilicity (rapid spreading of liquid water, low contact angle) arises from partial deprotonation of silanols and the formation of extended hydrogen-bonded water films, even though many individual silanols remain poorly engaged in hydrogen bonding.^391,395^ However, phase analysis has shown that the 3650 cm^–1^ OHs are oriented opposite to the silanols and are localized over siloxane-rich regions.^395^ From this, it has been concluded that hydroxylated amorphous fused silica is molecularly hydrophobic: incoming water preferentially hydrogen-bonds to other water molecules while many surface silanols and siloxane bridges remain only weakly engaged, despite the surface being macroscopically hydrophilic by wettability criteria.^395^ Consistent with this notion, at the fused silica/water interface, combined phase-resolved and time-resolved SFG with DFT-MD has shown that a high-frequency band near 3660 cm^–1^ arises not from silanols but from very weakly hydrogen-bonded water OH groups whose hydrogens point toward the silica surface. MD simulations demonstrate that these “hydrophobic water” molecules are located next to siloxane bridges in patches where the silanol density is low, and that the population of such quasi-free OH groups increases as the silanol density is reduced (e.g., by thermal treatment), in one-to-one correspondence with siloxane-rich domains.^391^ Also, the vibrational lifetimes and reorientation dynamics of these quasi-free OH groups (≈1 ps) indicate that, dynamically, water above siloxane patches behaves like water at a hydrophobic surface.^391^

**Figure 12:**
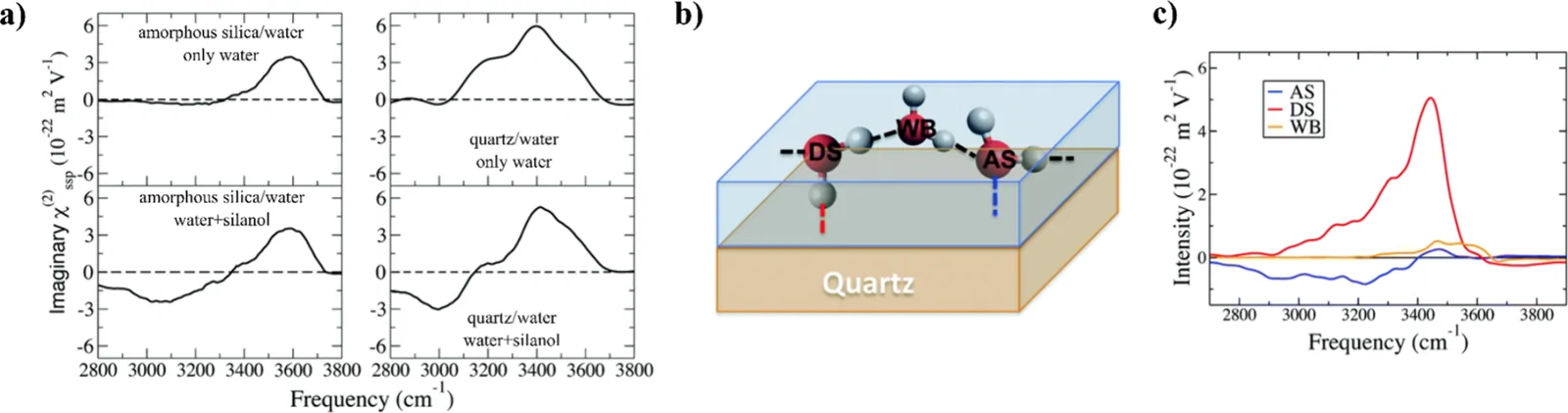
Density functional theory MD simulations reveal spectral contributions of water to the Im χ^(2)^(ω) spectra of the electroneutral SiO_2_–water interface (at the point of zero charge). (a) Spectra of the amorphous SiO_2_ interface in contact with water (left panels) and of a quartz–water interface (right panels). The top panels illustrate the spectral contributions from water alone, while the bottom panels also include signals from surface silanol groups. The simulations provide evidence of distinct spectral signatures of silanols and water molecules. Despite different spectral features of silica and quartz, both interfaces exhibit the same sign and frequency for the silanol and water responses. The most common binding motifs of interfacial water molecules in the BIL (binding interfacial layer) are depicted in (b), and their contribution to the interfacial water response is dissected in (c): water donating H-bond to SiO_2_ (DS), water accepting H-bond from SiO_2_ (AS), and water molecules non-H-bonded to SiO_2_ but H-bonded to AS or DS (WB). Adapted with permission from Pezzotti et al., *Phys. Chem. Chem. Phys.* 2019, 21, 22188–22202.^389^ Copyright 2019 Royal Society of Chemistry.

Even relatively simple electrolytes such as KCl reorganize the BIL without fundamentally changing deeper hydration layers.^389,396^ At the quartz/water interface, inner-sphere adsorption of K^+^ at silanol sites decreases the number of water–water and water–silanol hydrogen bonds in the BIL. Yet, this hydrogen-bonded network of water at silica and alumina shares some similarities with water next to extended hydrophobic interfaces.^397^ Heavy divalent metals amplify this effect and can drive true hydrophobization:^398,399^ In Pb­(NO_3_)_2_ solutions, SFG spectra of the silica–water interface develop pronounced minima in overall intensity at intermediate and high pH, accompanied by the emergence of weakly hydrogen-bonded/dangling OH features around 3600 cm^–1^ that closely resemble hydrophobic free-OH modes.^399^ By decomposing the total response into contributions from surface-bound (Stern-layer) and more distant water, Raji et al. show that these 3600 cm^–1^ modes arise from water in the immediate vicinity of inner-sphere Pb hydroxo complexes and from OH groups on Pb–OH species themselves. These species interact only weakly with the surrounding water, which is also accompanied by large advancing contact angles, and demonstrate hydrophobization of an originally hydrophilic silica surface through Pb adsorption and associated restructuring of the hydration layer.^399^ Similar effects have been observed for Cu^2+^ and Zn^2+^. For all three ions,subtle ion-specific effects in the adsorption of metal hydroxo complexes or hydroxides have been explained by their hydration energy and distinct coordination.^398^ Hence, hydrolyzable multivalent ions can tune the balance between water–surface hydrogen bonds (promoting hydrophilicity) and water–water bonds in the BIL (promoting hydrophobicity),^389,398,399^ with corresponding spectral signatures in the relative weight and position of the 3200–3400 cm^–1^ bands versus emergent ≈ 3600–3660 cm^–1^ features.^389,398,399^

In general, for silica there appears to be consensus that hydrophilicity is associated with silanol-rich regions where water in the BIL forms strong hydrogen bonds to the surface and to underlying water, with hydrophobicity appearing locally wherever siloxane bridges or specifically adsorbed complexes interrupt the ideal silanol–water network, forcing water to form predominantly lateral hydrogen bonds and leaving one OH group weakly hydrogen-bonded or quasi-free, spectroscopically visible near 3600–3660 cm^–1^.^389,391,395^ Similar spectroscopic signatures of water interactions have been observed at alumina interfaces,^400–402^ for which the aluminol groups have been suggested to bind more strongly to water than silanols.^403^ To better understand these differences between silica and alumina, mica interfaces may be a promising route, as both aluminum and silicon atoms are present, and differently bound OH groups give rise to distinct SFG spectral features.^354^

The interaction of water with TiO_2_ has many features in common with silica–water interfaces, despite subtle differences in the surface acidity of OH groups.^404^ For TiO_2_–water interfaces, SFG spectral signatures have been ascribed to similar interaction motifs as found for quartz and silica.^404–406^ Yet, hydrophilicity may be enhanced by the subtle balance between chemisorbed and physisorbed water with distinct spectral contributions,^407^ and the hydrophilicity has been related to the macroscopic friction.^266,408^ An intriguing property of TiO_2_—in contrast to silica—is its lower band gap and potential for photochemical reactions: Irradiation with UV light has been reported to increase hydrophilicity, as reflected in the interfacial vibrational spectra.^409^ Computational studies have related this photoinduced hydrophilicity to dissociative water adsorption,^347^ but adsorption of small molecules may also play a key role in the macroscopic hydrophilicity.^346^

Peculiarly, the SFG spectra of MgO–heavy water interfaces are dominated by a high-frequency OD peak, which resembles the free OD peak at the air–D_2_O interface.^410^ This may be even more surprising in light of water having a high tendency to dissociate at wet MgO interfaces.^411^ Adding another unresolved aspect, the absence of spectroscopic signatures of water hydrogen bonding to MgO is maintained across a rather wide range of solution pHs, yet these signatures emerge when the liquid is flowing over MgO.^350^ The latter observation was related to the dissolution of MgO. However, the hydrophobicity or hydrophilicity, as judged from the interfacial vibrational spectra of MgO–water interfaces, apparently is controlled by factors that have not yet been fully elucidated.

In contrast to the wealth of information on the structure and hydrogen-bonding interactions of water at oxide interfaces, much less detailed insights have been obtained regarding its dynamics.^388^ Only 20 years ago, the first spectral hole-burning experiments at silica–water interfaces were reported, which found that spectral diffusion of hydrogen-bonded water next to silica is similar to that in bulk water.^412^ Also, vibrational energy relaxation was found to be similar to bulk water, except for the relaxation of the nonbonded OH groups at a hydrophobized silica interface.^412^ Since then, the population relaxation behavior of water has been refined, showing that the decays depend on the OH/OD frequency,^413^ the charge state,^413^ and the screening of the electrical potential in the aqueous phase.^414^ Conversely, screening appears to have little effect on population relaxation at the Al_2_O_3_ interface, where relaxation is faster than at silica–water interfaces, which has been ascribed to the more red-shifted OH stretching frequencies that can either open additional relaxation channels or facilitate proton transfer.^415^ Very likely, the sensitivity of these dynamics to the multitude of factors reflects the different subensembles probed in an SFG experiment as the properties of the aqueous phase are varied, such as pH or ionic strength. Apart from these insights into water dynamics, 2D-IR experiments have also elucidated the ion dynamics next to silica interfaces using porous silica by probing spectral diffusion and orientational dynamics of SeCN^–^ ions as a function of pore size.^344,357^ From these studies, it has been suggested that the dynamics, which also reflect hydrogen-bond dynamics in the hydration shell of the ion, differ at the pore wall from the core of the silica pores, yet the transition between these different dynamic regimes is not fully understood.^344,357^

#### 4.2.2 Other Minerals

The hydration of more ionic solids is most intensively studied for polycrystalline fluorite (CaF_2_). The interfacial vibrational spectra of water in contact with fluorite are dominated by a hydrogen-bonded OH stretching band.^369,416^ The ordering of these hydrogen-bonded water molecules, however, mainly results from the electric field-induced breaking of the symmetry due to the preferential dissolution of F^–^ ions.^369^ Only at high pH values, adsorption of OH^–^ ions to the interface gives rise to a high-frequency OH stretching contribution in the SFG spectra.^369,417^ The presence of this high-frequency OH stretching band has been occasionally related to the hydrophobicity of fluorite surfaces at high pH.^417^ Interestingly, contact angle experiments together with spectroscopic investigations have suggested that only the (100) and (110) surfaces are completely hydrophilic, while the (111) surface seems less hydrophilic.^418^ As such, for the bulk of the experimental studies using polycrystalline fluorite, the hydrophilicity of these interfaces may also be rather heterogeneous on a microscopic level, similar to the conclusions that have been drawn for SiO_2_. For other calcium-based minerals, such as calcite, the characteristic spectral signatures of water interaction with calcite seem to be absent, which has been attributed to the negligible surface charge.^364^ The similar mineral magnesite, which has a different point of zero charge, instead exhibits signatures of aligned hydrogen-bonded water in the electrical double layer.^364^ Similar to calcite, gypsum (a CaSO_4_ hydrate) shows few spectroscopic signatures of hydration in the SFG spectra.^366^ Nevertheless, water molecules at a wet gypsum interface have been shown to exhibit a remarkable azimuthal anisotropy, demonstrating that not only the out-of-plane but also the in-plane hydrogen-bonding can be dictated by the contact with the solid.^367^ This high degree of ordering has also been observed with 2D-IR spectroscopy, which showed that water within the crystal exhibits dynamics on remarkably long timescales (∼30 ps at room temperature).^368^ A remarkable example of other minerals that have been studied with vibrational techniques is hexagonal boron nitride (hBN): Despite hBN being isoelectronic to graphene—the prototypical hydrophobic interface of Section 3.2—hBN supported on silica substrates in contact with water exhibits many spectroscopic signatures of a charged, hydrophilic surface, which has been explained by chemisorption and physisorption of negatively charged hydroxide ions to the interface.^265^

#### 4.2.3 Metals

Despite the relatively large number of nonlinear vibrational studies at metal interfaces, which focus on the chemisorption or physisorption of electrochemical intermediates,^377,388,419^ very little spectroscopic insight into the interaction of these metal electrodes with water has been obtained. This lack of spectroscopic insights, which makes categorization of hydrophobic or hydrophilic interfaces challenging, can be largely explained by the experimental challenges posed by the very strong nonresonant background of conducting electrons in metals, which obscures both SFG^375,380,420^ and 2D-IR signals.^374,377^ As such, different strategies have been applied to reduce these nonresonant contributions, including delicate control of the metal thickness^372,380^ or by adjusting the pulse sequence such that the nonresonant contributions have largely decayed.^421^

Early experiments have observed two distinct hydrogen-bonded OH stretching peaks for water next to Pt or Au electrodes,^422^ the origin of which has remained debated, as the apparent peaks can be caused by refractive index variations and therefore merely reflect the corresponding bulk properties of the electrolyte rather than molecular interactions with the electrode.^375,380^ Nevertheless, upon decreasing the metal thickness below 2 nm, the measured SFG response differs from what would be expected based on the refractive index,^380^ which suggests that for these thicknesses indeed the very interface of the electrode can be probed and that there is a significant portion of hydrogen-bonded molecules at the interface.^380^ Interestingly, early work by Baldelli et al.^423^ determined the solvation of Pt electrodes using electrolytes in mixed water/acetonitrile solvents. These results suggest a preferential presence of water at Pt electrodes at negative potentials, whereas at positive potentials acetonitrile is detected. It seems that bulk refractive index changes alone cannot fully explain these preferences, and the proposed preferential interaction mediated by solvated ions can be readily detected.^423^ In all of these studies, the vibrational OH-stretching response of water next to the metal has been reported to be dominated by a broad H-bonded water signature, similar to the very hydrophilic oxide or mineral interfaces discussed above, with no pronounced indications of dangling OH groups. While the presence of hydrogen-bonded water next to a metal surface is also predicted by simulated SFG spectra, these simulations also predicted marked contributions from weakly hydrogen-bonded OH groups characteristic of the interaction of water with a more hydrophobic interface.^424^ The dangling OH vibrations characteristic of hydrophobic interfaces were isolated by introducing a temporal delay between the visible and infrared pulses, thereby suppressing the rapidly dephasing nonresonant response. Unless the electrode was oxidized, these free OH groups have been observed at positive and negative potentials applied to the electrode.^421^

Similar to SFG, direct 2D-IR measurements of water dynamics at metal electrodes remain technically challenging. Nevertheless, nitrile reporter groups in conjunction with surface-enhanced 2D-IR have been used to resolve overlapping H-bonded and non-H-bonded nitrile subensembles at gold interfaces. Time-dependent 2D lineshapes suggested that exchange between H-bonded and non-H-bonded nitriles occurs somewhat faster at negative applied potentials than at positive potentials, while spectral diffusion dynamics changed only moderately with potential.^373,425^

### 4.3 Ion Adsorption/Desorption

In this subsection, we summarize nonlinear vibrational studies of ion desorption and adsorption at solid–liquid interfaces. We focus on three distinct aspects: desorption from the solid, i.e., the dissolution process, nonspecific adsorption of ions in the electrical double layer, and specific ion effects at the solid interface. Complementary information is provided by surface-enhanced 2D-IR measurements of tethered nitrile probes, including work by Zanni and co-workers and by Bodine, Tokmakoff, Lewis, and co-workers, discussed in Sections 4.1, 4.2.3, and 4.5.2. These measurements probe local reporter dynamics rather than the same depth-weighted water response as that of SFG.

#### 4.3.1 Dissolution

The most fundamental ion desorption/adsorption process at a solid–electrolyte interface is arguably the dissolution of the solid into the liquid or, less straightforward to study in a controlled manner, the growth of a solid from a supersaturated solution. Despite this obvious fact, the dissolution process has often been overlooked in studies using nonlinear vibrational spectroscopy, because the solids are frequently only sparingly soluble. Only in recent decades has it become apparent that interface-specific spectroscopies are critically affected by such dissolution processes and are therefore well suited to study them spectroscopically. Yet, more than 20 years ago, it was already recognized that SiO_2_ surfaces change over time during an SFG experiment^426^ and that the vibrational spectrum of CaF_2_–water can be altered by surface dissolution products.^427^

The sensitivity of interfacial spectroscopies to dissolution processes arises from the accumulation of dissolution products at the interface, which can alter interfacial solubility, pH, or charge screening, all of which affect the SFG-detected orientation of interfacial water molecules. For SiO_2_–electrolyte interfaces, it was shown in 2014 that flowing the liquid in contact with the solid is an efficient way to reinitiate the dissolution process, with profound effects on the surface charge and the interfacial vibrational spectra.^428^ Although the dissolution of silicic acid species into solution is rather slow, the generation of a steady-state concentration of these species within the length scales probed by SFG can be accomplished on a time scale of ∼ 1 h, a time scale that can be readily sampled with SFG.^428^ Later, it was shown that this dissolution process strongly depends on solution pH.^429^ In fact, detailed analysis of the dissolution process indicates that silica dissolution and the accumulation of silicic acid slightly reduce the interfacial pH, which further catalyzes the dissolution process.^430^ For industrially relevant SiO_2_-based glasses, the dissolution or corrosion process has been shown, using SFG, to become more complex, as both the local concentration and molecular interactions with the alkali cations determine the surface properties.^431,432^ Not only can the dissolution process be followed in great molecular detail using SFG, but also the condensation of silica precursors to form SiO_2_ at the air–water interface^433^ or at the silica–water interface.^434^ Knowing the surface properties of such grown interfaces helps elucidate routes to disperse such silica species in solution.^434^ In contrast to SiO_2_, the dissolution process is much less understood for other oxide surfaces. However, it has been suggested to be pivotal for understanding their interfacial properties, for instance, in the case of MgO.^350^

For ionic solids, such as CaF_2_, the dissolution or the adsorption of the constituent ions from solution is well established. As the surface potential arises from the preferential dissolution of F^–^, addition of an excess of Ca^2+^ or F^–^ can result in the incorporation of these ions into the crystal lattice at the interface and a change of the charge state.^427,435^ In fact, providing fresh liquid by flow can reinitiate the dissolution process and even change the sign of the surface charge,^428^ and the dissolution process can be macroscopically modeled.^436^ For calcite and magnesite, their very different interactions with water, although they differ only by the metal cation, have been proposed to originate from their different dissolution.^364^ For ionic solids, spectroscopic studies on the inverse process, nucleation and growth of crystals at the solid–liquid interface, are scarce but hold great promise for gaining detailed insights into important physiological processes, such as biomineralization.^365,437,438^

#### 4.3.2 Double Layer Formation

Adsorption of ions to interfaces within EDLs is often dissected into a near-surface (Stern) layer and a diffuse layer. For details of this EDL model, we refer the reader to Section 3.3.3, as the same concepts apply to soft and solid interfaces. To disentangle the inherently entangled, interfering terms contributing to the vibrational response within the EDL (i.e., the χ^(2)^(*ω*) and χ^(3)^(*ω*) terms in Section 3.3.3),^439^ concepts that have been used at soft interfaces have also been applied to solid–liquid interfaces, such as using different polarization combinations,^440^ different experimental beam geometries,^441,442^ or combinations with other spectroscopies.^439^ In contrast to charged, soft interfaces, solid–liquid interfaces offer unique opportunities to obtain independent experimental insights into interfacial potentials: The independently determined zeta potential^443^ allows for reducing the parameter space by constraining the potential at the boundary of the diffuse layer to the zeta potential.

With phase-resolved SFG experiments, the interference between the Stern/bound layer contributions and the diffuse layer contributions could be fully disentangled for both silica^444^ and alumina–electrolyte interfaces.^445^ These experiments have revealed that, irrespective of the pH of the solution, the spectral shape of the interfacial vibrational spectra ascribed to the diffuse layer is the same, in contrast to the Stern/bound-layer spectra (Figure 13). As such, the phase-resolved experiments provided evidence that, in the diffuse layer, the breaking of the centrosymmetry can be fully explained by the interfacial potential at the boundary of the interfacial layer. Notably, although the spectral shape of the diffuse-layer spectra is rather insensitive to the exact details, such as ion identity or surface charge, the magnitude is sensitive to these parameters because it approximately scales with the electrical potential at the diffuse-layer boundary. This potential is highly sensitive to surface charge and screening within the Stern/bound layer,^446^ and therefore, any specific-ion effect in the Stern/bound layer also impacts the diffuse-layer spectra. Notably, as the ionic strength of the electrolyte determines the decay length of the electrical potential and, as such, the probing depth of the SFG experiments, the probed properties of water also markedly depend on the ionic strength. For instance, the vibrational energy relaxation time determined from time-resolved SFG experiments at silica–water interfaces has been shown to vary markedly with ion concentration,^414^ with a transition from bulk-like behavior at long probing depths to slower relaxation dynamics at short probing depths. Yet, the concentration dependence of the vibrational relaxation time at the silica interface appears more complex, as the high ion concentration in the double layer also affects the hydrogen-bond connectivity near the surface, which depends on the interfacial surface charge.^447^ For the charged CaF_2_–water interface, 2D-SFG experiments have pointed in a similar direction: Despite the reduced extent of the hydrogen-bonded network at the interface, preferred orientation results in rapid exchange of vibrational energy.^448^ These examples highlight nonlinear vibrational spectroscopic insights into the structure and dynamics within the double layer adjacent to charged solids. We note that these spectroscopies have similar sensitivity to double layers next to metal electrodes,^449^ yet we will discuss these studies below in Section 4.5.

**Figure 13:**
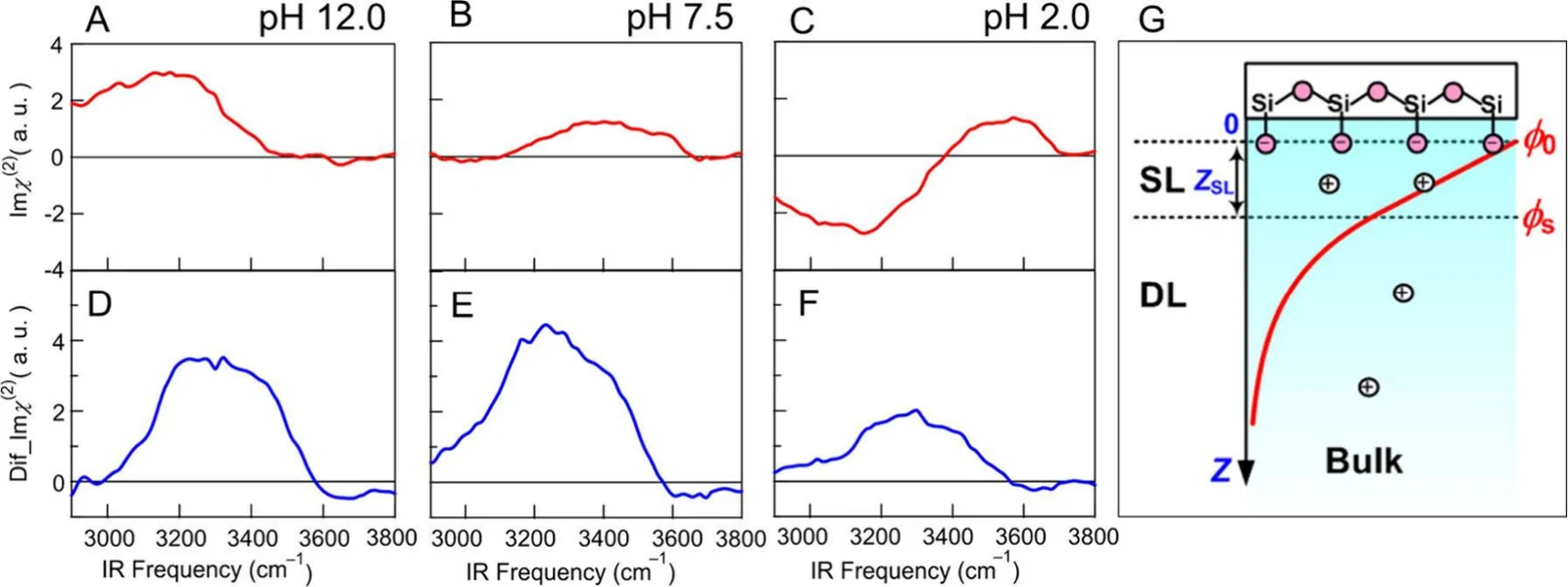
Phase-resolved SFG experiments can disentangle the Stern layer (bound interfacial layer) and the diffuse layer spectra at silica–water interfaces. Stern layer spectra at pH 12.0 (a), 7.5 (b), and 2.0 (c) exhibit marked pH-dependent variation, indicating distinct surface-binding motifs for interfacial water molecules on silica interfaces of different charge. Although the magnitudes differ due to different potentials at the boundary of the Stern layer, the diffuse layer spectra at pH 12.0 (d), 7.5 (e), and 2.0 (f) share a common spectral shape. The different contributions of the electric double layer are illustrated in (g) SL: Stern layer; DL: diffuse Gouy–Chapman layer; *Z*_SL_: thickness of the Stern layer; ϕ_0_: potential at the silica surface (surface potential); ϕ_S_: potential at the edge of the Stern layer (Stern potential). Reproduced with permission from Wei et al., J. Am. Chem. Soc. 2023, 145, 8833–8846.^444^ Copyright 2023 American Chemical Society.

#### 4.3.3 Specific Ion Effects

Given the insensitivity of the diffuse layer to ion-specific effects (discussed in Section 4.3.2), the arguably common ion specificities at interfaces of aqueous electrolytes with solid interfaces are almost exclusively confined to the very interface (Stern layer). The sensitivity of the total response of water in the diffuse and bound layers to the size of the ions of the electrolyte has already been reported more than a decade ago, based on SFG intensity spectra for silica^396^ and CaF_2_.^427^ At neutral pH, on a positively charged CaF_2_ surface, specific ion effects are predominantly observed as a function of anion identity, whereas at a negatively charged silica interface, cation identity is the dominant factor in interfacial structure. Interestingly, for silica, the trend in SFG intensity as a function of cation size has been reported to flip at low and high pH relative to neutral pH.^450^ The dependence of specific ion effects on pH—and, as such, surface charge density—could only be explained by the delicate interplay between electrostatic, hydration, and dispersion interactions within the Stern layer. While earlier studies predominantly discussed the total water orientation in both the Stern and diffuse layers, separating these contributions (using concentration series, phase-resolved SFG spectra, or zeta potentials; see Section 4.3.2) provided insights into the molecular-level origins of ion-specific effects. At least at neutral pH, the marked ion-specificity at the silica interface could be explained by different water orientations in the Stern layer for differently hydrated ions—in contrast to other effects, such as ion–surface interaction potentials or dielectric saturation, which have sometimes been invoked to explain specific ion effects.^451^ The qualitatively different trends of specific ion effects at low and high pH values seem to be predominantly related to different ion-specific water orientations in the Stern layer.^446^ On silica interfaces, the ion-specific spectroscopic differences in all of these studies are rather pronounced, although the structural differences in the EDL are somewhat subtle for monovalent electrolytes. For divalent electrolytes, SFG spectra have reported much more pronounced changes in the EDL with charge reversal of the interface.^398^ Similar to silica, the enhanced effect of divalent cations as compared to monovalent salts has been studied more systematically on Al_2_O_3_ interfaces,^452^ and also here, divalent cations were found to have a higher probability of adsorbing to the Stern layer. Remarkably, while the structure of the EDL is very sensitive to the valency of the ions, the vibrational relaxation dynamics of water probed with time-resolved SFG experiments are hardly affected by the identity of the counterions.^452^ The general trend that divalent ions tend to be more attracted to the surface also holds for anions interacting with the positively charged fluorite interface.^427,453^ However, for ionic fluorite, ion–surface interactions can also be indirect, such as ions in the electrolyte limiting the solubility of the fluoride ions due to the finite solubility product. This interplay may be the reason for the observation of both cationic and anionic specific ion effects at the fluorite interface upon flowing the liquid, the details of which are still poorly understood.^453^

### 4.4 Chemical Reactivity at Solid Interfaces

In this subsection, we summarize nonlinear vibrational studies of chemical reactivity at solid–liquid interfaces. The focus is on representative bond-breaking, bond-forming, and chemisorption processes at the interfaces of low-complexity aqueous electrolytes.

#### 4.4.1 Surface Acidity

The most straightforward process that involves breaking and formation of covalent bonds at solid interfaces is the reactivity of surface OH groups, e.g., silanol groups of SiO_2_. Such OH groups determine the surface charge and their pH-dependent degree of protonation/deprotonation, the point of zero charge (pzc). To extract the surface reactivity of the interfaces, it is indispensable to separate the vibrational response of these interfacial OH groups from the remaining water molecules,^358,389^ as already partially discussed in the preceding section. For SiO_2_ interfaces in contact with aqueous electrolytes, such studies have directly revealed that the interfacial p*K*_a_ of these silanol groups is 4.8.^444^ While this p*K*_a_ largely explains the pH-dependent surface charge, even for nanoporous silica interfaces,^454^ there has been computational evidence for a second subset of silanol groups with a higher p*K*_a_ value,^360,455^ which is also reflected in the interfacial vibrational spectra.^456,457^

Analogously, the interfacial p*K*_a_ values of alumina–water interfaces have been inferred.^400,403,415,445,458^ Although, as for SiO_2_–water interfaces, the spectral variation with pH points toward equilibria with distinctively different acidities, the transition at low pH values has been predominantly ascribed to the protonation of surface −OH groups to form −OH_2_^+^ (with an approximate p*K*_a_ of 3.7), yet the protonation/deprotonation of differently coordinated Al-OH groups (AlOH, Al_2_OH, and Al_3_OH groups) seems to still contribute at remarkably high pH values.^445^ Depending on the crystal facet, the different population of these distinct surface terminations gives rise to different interfacial vibrational responses, as demonstrated by computational SFG spectra.^400^ Such site specificity of the protonation response may explain the discrepancy between the pzc of alumina powder and monocrystalline interfaces.^458,459^

In line with the observations for alumina, the interfacial chemistry of thin TiO_2_ films has been spectroscopically observed to be much richer than that of silica, with both protonation and deprotonation of Ti-OH groups occurring, resulting in a higher pzc (∼5.5) of TiO_2_ as compared to SiO_2_.^404,460^ Alternatively, the different protonation equilibria may also be explained by chemisorption of water molecules. Phase-resolved SFG experiments have additionally indicated that, depending on the surface treatment, chemisorbed and physisorbed water can be experimentally distinguished,^407^ a finding supported by computational spectroscopy.^405,407^ The mechanism and magnitude of this chemisorption are strongly related to the surface defect density, as revealed by systematically reducing the water concentration in methanol/water mixtures in contact with TiO_2_.^461^

Similar, somewhat controversial discussions of chemisorption/physisorption of water have been undertaken for MgO–water interfaces.^411^ The spectroscopically relatively high pzc of MgO has been conceptually rationalized as arising from chemisorption of OH^–^, which serves as the source of surface hydroxylation at MgO interfaces.^462^ This assignment is also supported by SFG spectra of MgO interfaces in humid environments,^410^ yet the exact location of the dissociated H^+^ and OH^–^ group is presumably not intuitive.^411^ Yet, the significance of the latter observations for MgO in contact with water remains unresolved, due to the instability of the interfaces.^462^

Remarkably, the reactivity of interfacial OH groups is very specific to the oxide mineral (e.g., the reactivity largely varies for Si, Al, or Mg oxides). Mixed oxides, therefore, provide an additional degree of freedom to tune the surface reactivities. The interaction of Al-doped silica with water in a humid environment has been investigated with SFG.^463^ This study found that only within a very narrow Al-doping range, a pronounced high-frequency OH stretching band together with a broad, low-frequency OH stretching band due to OH groups pointing toward the surface is present, which suggests that strong interaction with the surface occurs at the boundaries between Al- and Si-rich domains.^463^

Mixed alumina/silicate oxides, in fact, can form bulk porous crystalline phases: zeolites. These pores are readily accessible to aqueous solutions, and their interfaces can exhibit high catalytic activity. Given the small pore sizes, aqueous solutions are confined on nanometer length scales, and confinement effects are discussed in more detail in Section 5.2. Nevertheless, the high catalytic activity of zeolites is also intimately connected to the high reactivity of interfacial OH groups and has been studied using femtosecond vibrational spectroscopy, which we briefly summarize here (for confinement effects, see Section 5.2). Using zeolites with only one water molecule per acidic site of the zeolite, the very anharmonic potential along the proton transfer coordinate has been characterized with the help of the 2D-IR spectra.^464^ This study has thus explored the very fundamental reaction step of Brønsted acid-based catalysis using zeolites, but could not address the stabilizing effect of surrounding water molecules due to the absence of extensive solvation of the dissociated proton. The effect of hydration in zeolites with higher water content was subsequently investigated with 2D-IR and ab initio MD simulations:^465^ Upon increasing the number of water molecules per acidic site from 1 to 8, the excess proton is relocated into the core of the zeolite pores, yet only two water molecules are sufficient to effectively ‘hydrate’ the excess proton.^465^ Besides characterizing the acidic sites, which are pivotal to interaction with water, 2D-IR experiments have also successfully evidenced the presence of bridging silanol groups, as well as hydrogen-bonded clusters of such silanol groups in zeolites and similar materials.^359^ In general, advances in the reduction of scattering signals in 2D-IR experiments using phase and polarization control^466^ and index-matching oils^467^ hold promise for much more detailed insights into heterogeneous catalysis in such systems.

#### 4.4.2 Chemical Surface Modification

In the presence of reactive molecules at the interface, irreversible reactions can lead to surface modifications and grafting. In this review, we partially address these studies already in the earlier section (Section 3.3.4) when the purpose of the grafting is to make the soft (molecular) interface with a liquid electrolyte optically accessible. Yet, spectroscopic insights can also help us understand the surface reactivity of the solid–liquid interface, which we discuss in this subsection.

For instance, silica surfaces can be modified by alkoxylation to become more hydrophobic. Yet, the stability of such coatings relies on chemisorption, a process that can be well-monitored by SFG spectroscopy, as chemisorbed and physisorbed molecules exhibit distinct vibrational signatures.^468,469^ Similarly, chemisorption of small organic molecules onto metals has been used to inhibit corrosion, a technologically relevant process that has been spectroscopically followed for metals such as copper.^385,470,471^ However, these reactions are intended to be irreversible, and spectroscopy is predominantly used to monitor the coverage. Conversely, chemisorption of small molecules on metal surfaces is often key to their electrochemical behavior and is often considered less irreversible. A noteworthy example here is the adsorption of CO on metals. The dynamics of CO adsorbed to Pt islands have been studied using 2D ATR-IR, and the spectral diffusion process of the intense CO stretching mode was isolated.^372^ Similarly, in addition to CO adsorption, the dissociation of H_2_ on Pt has been interrogated spectroscopically with 2D-IR, and the atomic hydrogens are coupled much more strongly to the metal than the CO vibrations.^472^ Also using plasmon-enhanced SFG experiments, adsorbed CO molecules have been used as reporters for the environment of the metal–electrolyte interface.^473^ Yet, CO adsorbed to metal interfaces is an obvious intermediate in electrochemical CO_2_ reduction, which is discussed, among other field-induced phenomena, in the next section.^473^ Similarly, cyanide can be used to follow electrochemical processes.^474^

The most intensively studied chemisorption reaction on metal surfaces is arguably the formation of self-assembled thiol-based monolayers (SAMs) on gold surfaces. In contact with electrolytes, such SAMs have been studied with both SFG spectroscopy^475^ and 2D-IR spectroscopy,^373,374,377,476^ often to introduce spectroscopically active reporters of electrochemical processes. The excellent abilities of such monolayers as local probes at metal surfaces rely on plasmonic enhancement, which is governed by several factors, including orientation, the electronic properties of the molecules, and the location of the SAMs on heterogeneous metal surfaces.^476^ Importantly, the vibrational spectra of these SAM molecules may change upon assembly of the monolayer. These changes can arise not only from variations in the molecular environment, but also from changes in vibrational–electronic coupling and plasmonic field enhancement at the metal surface. Often these monolayers on metal electrodes are considered as ‘innocent’ molecular reporters (i.e., molecules that have a high surface propensity but otherwise do not perturb the metal–liquid interface via electronic coupling), and the presence of gold merely distorts the phase of the 2D-IR signals,^376,388^ but otherwise does not affect the properties of the adsorbed molecules, such as their transfer of vibrational energy.^477^ While this assumption may hold for sufficiently large separations between the vibrational probe and the metal interface, for very small thiols adsorbed on gold, the lineshapes of the 2D-IR spectra change drastically with applied potential.^374^ To understand these varying lineshapes, a phenomenological model based on the mixing of vibrational and plasmonic states must be considered. Such a model explains many of the potential-dependent spectral features shown in Figure 14, yet the extent of the spectral distortions due to mixing depends on the coupling strength between these states. Exactly this coupling strength may determine the signal enhancement in these spectroscopic settings.^374^ As such, the current understanding of the spectroscopic signatures of SAMs at metal surfaces is still incomplete; future methodological developments, such as different polarization combinations,^478^ may help add the missing pieces to this puzzle.

**Figure 14:**
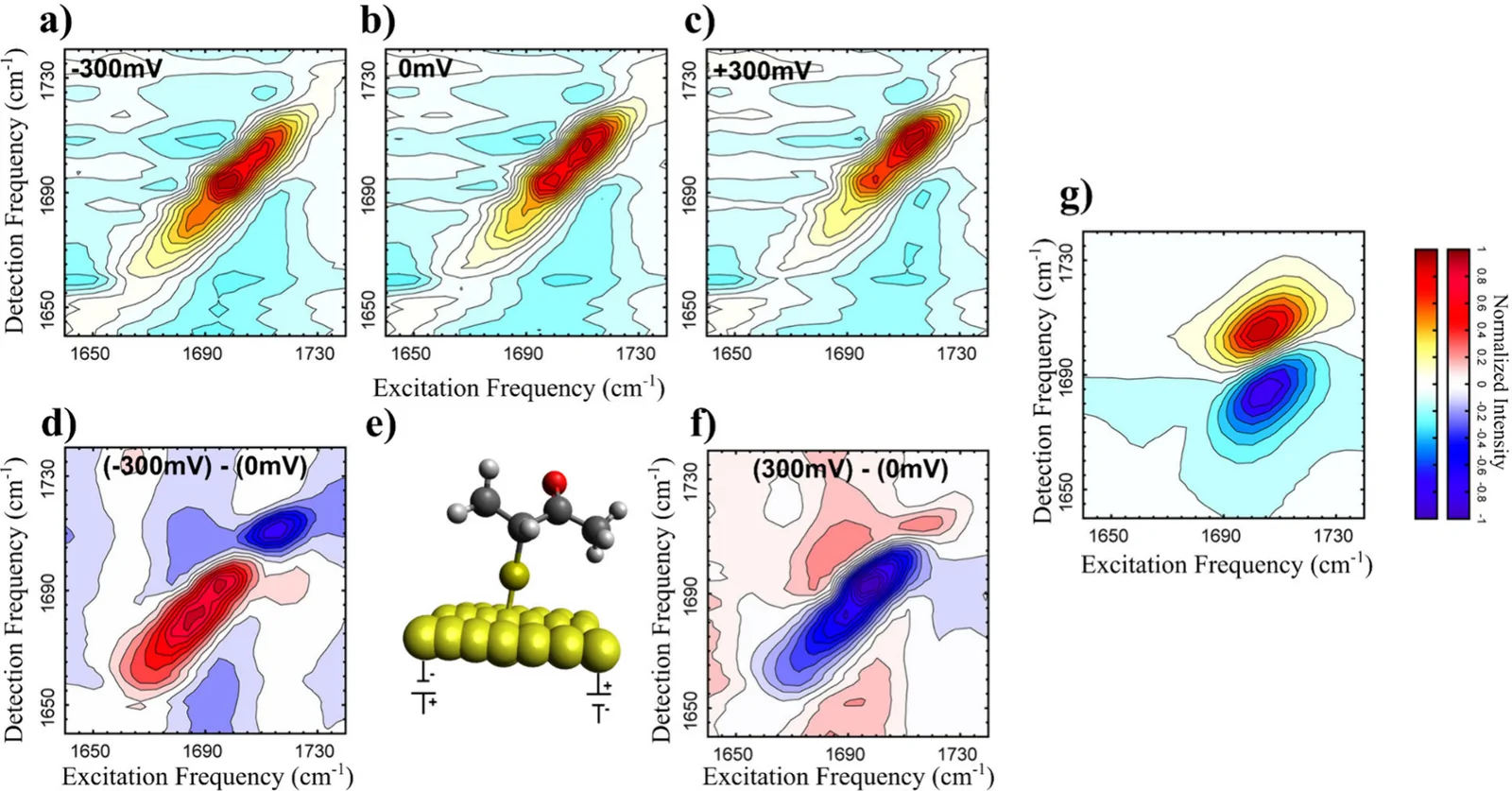
2D-IR evidence of mixing between the vibrational states of 3-mercapto-2-butanone chemisorbed to a gold surface and the plasmonic states of the gold. Panels (a)–(c) show 2D-IR spectra at C=O stretching frequencies as a function of applied potential at *E* = −300, 0, and 300 mV vs Ag/AgCl. To emphasize the mixing-induced shifts, differential spectra at *E* = −300 and 300 mV, referenced to 0 mV vs Ag/AgCl, are shown in (d) and (f). The structure of 3-mercapto-2-butanone chemisorbed to gold is schematically shown in (e). For comparison, the solution spectrum of 3-mercapto-2-butanone, which exhibits narrower, shifted signals as compared to (a)–(c), is shown in (g). Adapted with permission from Bodine et al., J. Phys. Chem. Lett. 2023, 14, 11092–11099.^374^ Copyright 2023 American Chemical Society.

#### 4.4.3 Interfacial Photochemistry

Nonlinear vibrational spectroscopy is inherently suitable to provide molecular-specific insights into photochemical processes. A large body of studies using solid photosensitizers has focused on TiO_2_ for such photochemical studies due to its optical band gap in the soft UV wavelength range and its photocatalytic relevance. Most of the experimental insights have been obtained from the vibrational response at OH stretching frequencies, and photochemical reactivity has been inferred by comparing the vibrational response before and after UV irradiation. As the OH stretch response can originate from surface Ti-OH groups, chemisorbed water, physisorbed water, or products of photocatalytic reactions, assignment is not always straightforward. Early work has demonstrated that the OH stretching SFG spectra of TiO_2_ in contact with humid gas change over time after UV irradiation, with relevance to photocatalytic water splitting and the UV-induced hydrophilization of TiO_2_.^409^ At the superhydrophilic (UV-irradiated) TiO_2_–water interface, OH groups pointing toward and away from the solid surface have been identified, which were ascribed to chemisorbed and physisorbed water.^407^ However, ab initio MD simulations have suggested that it is challenging to spectroscopically discriminate between chemisorbed and physisorbed water, as their vibrational signatures are very similar.^405^ At the same time, computational studies on water at TiO_2_ have further indicated that the presence of photoexcited holes at the interface markedly enhances chemisorption of water and thereby explains the increased magnitude of the experimentally observed SFG response after irradiation.^347^ Related to that, laser irradiation has been shown to generate strongly hydrogen-bonded OH groups, such as Ti-OH^+^-Ti or TiOH_2_^+^ surface groups. Such treatment affects catalytic activity, and the surface sites can be quenched by fluoride at low pHs, as evidenced by the vibrational response of the OH groups.^479^ Despite these detailed insights into irradiation-induced changes to the OH response, indirectly probing OH groups cannot unambiguously evidence the UV-induced chemisorption process. Conversely, Ti-O vibrations are directly sensitive to the chemisorption process, as the Ti-O bonds are formed only upon chemisorption. Indeed, a recent study has demonstrated that the SFG signal characteristic of the Ti-O vibration is drastically enhanced after UV irradiation,^461^ providing direct evidence for UV-induced chemisorption of water. Together, these studies have provided a nuanced view of UV-induced changes to the interfacial structure of TiO_2_–water. The dynamics of these structural changes have been elucidated using UV pump–SFG probe experiments.^349,480^ These experiments have provided evidence that, after the instantaneous generation of electron–hole pairs, the subsequent reactions are largely independent of solution pH. These reactions involved hole-assisted deprotonation of existing Ti-OH groups and photoinduced adsorption (chemisorption) of OH, all of which occurred on a time scale of a few to tens of picoseconds.^349^

In addition to TiO_2_-catalyzed water splitting, photochemical conversion of organic substances has also been studied. Interestingly, the structure of photo-oxidized surfactant layers at TiO_2_ depends on the adsorption state: physisorbed surfactants are depleted after photooxidation, while chemisorbed monolayers remain in contact with the solid, with the gradual oxidation disrupting the monolayer structure.^481^ In the photo-oxidation process of methanol, SFG spectra have successfully identified different reaction products via their vibrational signatures.^482^ The rich vibrational signatures at the interface have also allowed discrimination of different binding sites of chemisorbed methanol and have related their UV-induced variations to their different catalytic activities.^483^ To photochemically convert CO_2_, the TiO_2_ surfaces must be decorated with catalytically active sites. Here, catalytically active Re-carbonyl metal centers are ideally suited for vibrational spectroscopic characterization because of the high cross section of carbonyl vibrations. Using these CO groups as reporters, 2D-IR spectra have evidenced coupling between the electronic states of TiO_2_ and the metal center: both the lifted degeneracy and the change in the CO transition dipole moment are consistent with DFT calculations of surface-bound/coupled Re centers.^484^ 2D-SFG experiments have revealed that the detected CO band exhibits surprisingly large inhomogeneous broadening, evidencing the structural heterogeneity of the adsorbed metal centers. Such experiments have further shown that the vibrational relaxation of these CO groups can strongly depend on the TiO_2_ crystal surface to which they are bound, indicating different couplings to the solid.^485^ The catalytic activities of differently bound metal centers remain to be isolated.^486^

In addition to the extensive studies of TiO_2_, other catalytic solids can also be assessed.^487^ In general, photocatalytic materials are not very compatible with nonlinear spectroscopic investigations due to pronounced light scattering and fluorescence. However, recent technological advances may help mitigate these challenges^478,488^ via the utilization of the full toolbox available to ultrafast spectroscopists. Using such approaches, even the real-time investigation of the photoinduced electron transfer from gold to water has been accomplished,^489^ which is otherwise challenging, as discussed in the following subsection.

### 4.5 Electrochemistry

Among aqueous electrolyte–solid interfaces, metal–electrolyte interfaces have high scientific and technological relevance. Due to the high impact of these systems, electrochemical applications of SFG—often aimed at mechanistic insights—have been extensively reviewed over the past decade.^338,419,473,490,491^ Over the past decade, tremendous technological progress in 2D-IR spectro-electrochemistry^388^ has made this method broadly available and widely used to study electrochemical phenomena using reflective or attenuated-total-reflection geometries. In this subsection, we aim to summarize the insights obtained in recent years and define electrochemical studies broadly as interfacial studies in which an external potential is applied between the solid and the liquid electrolyte. We first provide an overview of experimental approaches to electrochemical studies, then focus on potential-induced changes in the electrolyte (e.g., the double layer) without chemical conversion, and on potential-induced chemisorption at the electrodes. The last subsection discusses electrochemical interconversions (oxidation and reduction) at the interface. However, we note that this categorization is somewhat arbitrary since it is often difficult to clearly distinguish these processes, which often occur simultaneously and/or are intertwined.

#### 4.5.1 Experimental Spectro-Electrochemical Geometries

In general, spectro-electrochemical SFG experiments are performed at flat electrodes, with the interface optically accessible from the electrolyte or the solid-electrolyte, depending on the transparency and thickness of the electrode and electrolyte for the visible and infrared beams. 2D-IR experiments have been realized in different ways, including reflection from the surface and transmission through thin films.^388^ However, in recent years, attenuated total internal reflection is most frequently used. A major challenge in all these spectro-electrochemical cells is the high reflectivity of the metal electrodes and the strong response of the mobile charge carriers within them to laser pulses. The general strategy to minimize this response is to keep the metal as thin as possible.^372,375,476^ Although the thin metal films can also be contacted directly,^492^ these metal layers are often deposited on transparent, conductive substrate layers, such as indium tin oxide (ITO)^372,375,377,476^ to establish electrical contact. The strong electronic response of the metal to the applied laser pulses can be utilized, as the thin films give rise to plasmonic enhancement of the sample’s response.^377,476,492^ The local electric field enhancement at the interface due to the plasmonic resonance can tremendously increase the surface sensitivity of the nonlinear vibrational spectroscopies even for ‘as-deposited’ metal films.^376^ Using well-defined, structured metal films, such as metal gratings, the plasmonic enhancement can be further increased and engineered for a specific task.^493^ Further enhancing local electric fields with tip-enhanced nanocavities has enabled SFG experiments with continuous-wave lasers rather than pulsed light sources.^494^ However, the response of the metal also distorts the signals due to molecular vibrations at the interface^375,376^ and these distortions—or couplings, depending on the point of view^476^ strongly depend on the applied potentials^375^ and the morphology of the metal layers.^476^ For SFG experiments, we caution the reader in particular that potential-dependent vibrational studies may reflect the potential-dependent refractive index of the metal layer,^375^ rather than the potential-dependent structure of the electrolyte—such as the alignment of dipolar solvent molecules—which has been interpreted in earlier SFG studies. Conversely, the refractive index of the bulk electrolyte hardly depends on the applied potential, and as such, the appearance of sharp vibrational resonances at very specific potentials (e.g., adsorption of acetate at large positive potentials^495^) renders such bulk effects unlikely. For 2D-IR experiments, in which the metal–electrolyte interface is often tested in attenuated total internal reflection geometry, background signals have been shown to be reduced by electrically insulating the metal films from the ITO substrate.^377^ Similarly, gating of a thin SiO_2_ film on top of a silicon substrate reduces the metallic response and allows for altering the surface charge.^371^ The use of silicon as a working electrode has further allowed for studying surface passivation processes of battery electrolytes.^386^ An intriguing way to push the boundaries toward thin electrodes for electrochemical purposes is the use of graphene as an electrode,^223^ which even provides access to technologically relevant fuel cell interfaces.^496^ Despite some notable exceptions,^226^ the graphene electrode has, however, been widely supported on substrates, and it can be challenging to separate the electrolyte response to the substrate from the response to graphene.^497^ Nevertheless, in all these experimental settings, nonlinear vibrational spectroscopies have provided unprecedented insights into the structure, dynamics, and reactivity at these interfaces.

#### 4.5.2 Molecular Properties at Electrodes

We start by reviewing spectroscopic insights into potential-dependent structural and dynamic changes of ions or molecules near electrodes without a focus on electrochemical reactivity. There have been a few potential-dependent studies of electrodes in contact with electrolytes using SFG. These studies include platinum,^422,423^ silver,^383^ gold,^421^ and graphene^223,224^ electrodes. All these studies have in common that some vibrational signatures of the electrolyte, such as the broad hydrogen-bonded OH stretching bands of water, exhibit continuous changes in intensity with applied potential. These rather continuously changing spectral contributions may, as discussed before (Section 4.2.3), arise from a potential-dependent orientation of these molecules or from variations in the refractive index of the metal^380^ with potential. Yet, some studies have provided evidence for less continuous variations of the spectral signatures as a function of potential: For interfaces of platinum electrodes with water, the continuous change of water’s SFG intensity with potential breaks down at highly positive potentials, which has been ascribed to signatures of altered electrode–water interaction due to the oxidation of the Pt electrodes.^422^ The potential-dependent solvation in mixed acetonitrile/water solvents, which has already been mentioned in Section 4.2.3, can be related to the chemical composition of the inner Helmholtz layer in the electrochemical context.^423^

For gold electrodes in contact with water, the spectral signatures of hardly hydrogen-bonded OH groups—similar to the free OH groups at the air–water interface—have been observed over a very wide range of applied potentials, which has been proposed to evidence the gold electrode remaining hydrophobic, irrespective of the applied potential.^421^ At the graphene–water interface, similar spectral signatures of free OH groups have been observed experimentally to be present only at negative potentials, while they disappeared at neutral or positive potentials.^223^ These spectral changes have suggested evidence of the asymmetric response of water to electric fields due to the molecular symmetry.^223^ Indeed, distance-dependent MD simulations confirm that the high-frequency spectral OH feature is a direct consequence of water molecules interacting with the graphene.^498,499^ However, phase-resolved experiments have shown that these detected OH groups do not originate from water molecules in the aqueous electrolyte, pointing toward graphene, but have opposite orientations. Their potential-dependent generation, together with their orientation, suggests that these OH signals originate from hydroxide ions electrochemically generated at sufficiently negative potentials, which are adsorbed onto the CaF_2_ substrate.^224,500^ This example highlights that high-frequency OH features, often interpreted as signatures of hydrophobic water, may originate from entirely different species, emphasizing the need for phase-sensitive measurements and careful assignment.

Another double-layer adsorption process that has attracted a lot of interest in recent years is the interaction of sulfate with metal electrodes, because the nature of the adsorbed species, whether it is SO_4_^2–^ or HSO_4_^–^ or other species, has remained debated.^501^ The SFG spectra of Pt in contact with dilute sulfuric acids show clear S-O vibrations present only at positive potentials.^502^ From the variation of these spectral signatures with applied potential, there have been two potential regimes with different Stark shifts identified, which have been ascribed to 3-fold coordinative interaction of sulfate with the Pt surface at moderately positive potentials and disruption of this coordination due to the adsorption of hydroxide at enhanced potential.^502^ More recent experiments did not confirm the presence of such a distinct second adsorption regime. Rather, the nonlinear frequency shift has been argued to result from the field-induced polarization of the sulfate at high potentials.^503^ The identity of the adsorbed ions has been assigned to SO_4_^2–^ and not HSO_4_^–^ using isotope exchange experiments,^501^ yet at gold interfaces contributions from bisulfate have been reported.^504^

These SFG experiments have largely revealed the electrolyte structure near the electrodes, whereas much of the molecular dynamics relevant to electrochemical kinetics remained unresolved. The dynamics of the water molecules in contact with the electrode have, in fact, been interrogated with 2D-IR: gold electrodes functionalized with nitrile groups exhibit distinct nitrile stretching peaks, ascribed to hydrogen-bonded and non-hydrogen-bonded CN groups.^425^ These two peaks are not only better separated in the 2D-IR spectra, but at long waiting times, there is evidence for the emergence of cross-peaks between both modes, which can be explained by chemical exchange (Figure 15).^425^ Hence, the temporal behavior of these cross-peaks—or the line shape—witnesses the formation and breaking of hydrogen bonds of water with the functionalized surface. Remarkably, these hydrogen-bond breaking and formation events occur on rather long timescales (tens of picoseconds), and the timescale is approximately 3-fold slowed down at positive potentials, as compared to negative potentials, directly evidencing potential-dependent dynamics of the near-electrode hydrogen-bonded network.^425^ These dynamics have further been shown to depend on the nature of the salt in the aqueous electrolyte.^373^ While such chemical exchange has been observed for water interacting with the electrode, it has not been observed for solvent interacting with electrolyte ions: the CN vibration of acetonitrile shifts markedly upon interaction with cations with high surface charge density. Again, 2D-IR experiments have revealed cross-peaks between acetonitrile in the solvation shell of cations and free acetonitrile; however, these cross-peaks could be fully explained by vibrational energy transfer, and the exchange dynamics have been concluded to occur on timescales longer than 100 ps.^505^

**Figure 15:**
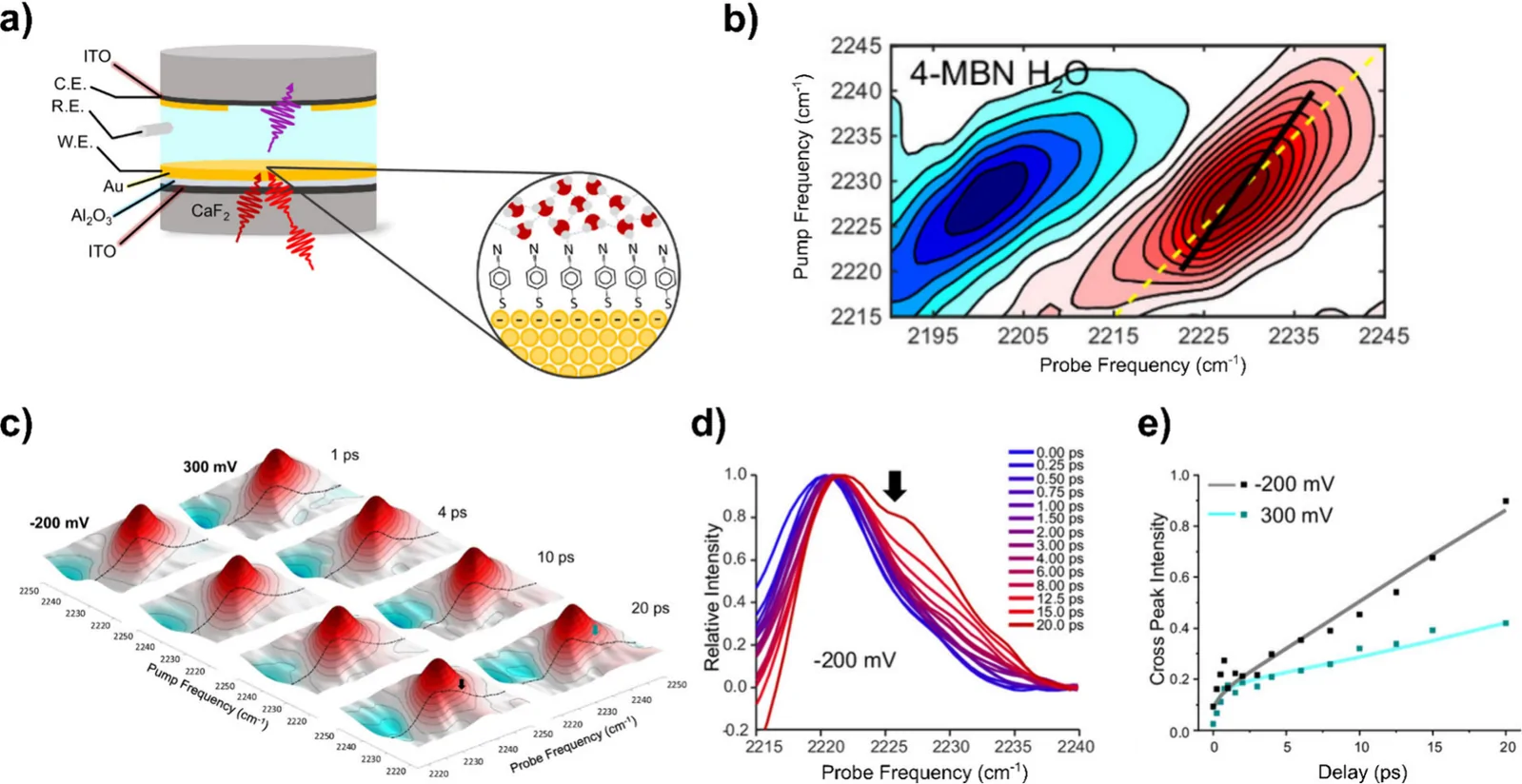
2D-IR spectroscopy of nitrile probes at the gold interface provides evidence for potential-dependent hydration dynamics at the gold electrode via the temporal evolution of the cross-peak dynamics between differently hydrogen-bonded nitriles. (a) Schematic for the 2D-IR spectro-electrochemical cell: the bottom CaF_2_ substrate is covered with thin films of ITO, Al_2_O_3_, and rough gold. C.E., R.E., and W.E. stand for counter, reference, and working electrodes, respectively. The zoom shows a schematic of the surface structure of the 4-mercaptobenzonitrile-functionalized gold surface. (b) 2D-IR spectrum of a 4-mercaptobenzonitrile monolayer submerged in water, with the black line showing the center line of the ground-state bleach peak. (c) 2D-IR spectra of a 4-mercaptobenzonitrile monolayer in water under different applied potentials and with various pump–probe delay times. The dotted line marks a slice at 2220 cm^–1^ on the pump axis. (d) displays the pump slices extracted from the −200 mV spectra from (c), where they are depicted as black dashed traces parallel to the probe frequency axis, and the downward arrows highlight where the cross-peak between the differently hydrogen-bonded nitriles emerges. (e) Cross-peak intensity as a function of delay time extracted from (d) at 2227 cm^–1^. Adapted with permission from the authors from Ryan et al., *Proc. Natl. Acad. Sci. U.S.A*. 2023, 120, e2314998120, published under the terms of the Creative Commons CC BY-NC-ND 4.0 license.^425^ Copyright 2023 The Authors.

The examples discussed so far mostly cover weak molecular interactions induced by the applied potentials; however, covalent attachments can also be initiated and are often the first step in electrochemical conversions. For instance, the strong interaction of thiols with gold, as shown in Figure 15 for electrode functionalization with vibrational probes, requires oxidation of the thiol and can be controlled by an external potential. This process has been monitored using SFG in a basic environment, and it was found that despite reductive desorption of octadecanethiol from gold, the thiols remained as an ordered layer at the gold–water interface,^492^ which may be consistent with the physisorption of conventional, charged surfactant at an electrode irrespective of the applied potential.^506^ Conversely, oxidative desorption of the self-assembled monolayer resulted in the dissolution of the adlayer.^492^ Yet, the remainder of a physisorbed layer after reduction of the self-assembled monolayer is not universal, but has been shown to strongly depend on the thiol: octadecanethiol remained as a physisorbed layer, while a decanethiol monolayer disintegrated after reduction.^475^ The occurrence of such electro-desorption processes is directly linked to the potential-dependent interaction between the thiols and the gold, which also manifests as the coupling of vibrational states with the electrode. The latter has been studied in great detail with 2D-IR (Figure 14).^374^ For many vibrational modes of self-assembled monolayers, their potential-dependent shift is consistent with a vibrational Stark shift,^377^ except when the external potential shifts chemical equilibria, such as the acid–base equilibria of a Brønsted base chemisorbed to the electrode.^507^ Similar to the thiol-based self-assembled monolayers, the potential-dependent vibrational shift of carbon monoxide adsorbed to Pt is consistent with a Stark shift,^372^ and at high potentials, the oxidative electro-desorption is observed via the disappearance of the vibrational signatures.^508^ Interestingly, the Stark shift of CO can also be used as a reporter of specific ion effects at gold electrodes.^509^ Based on these results, the unique catalytic activity of certain ions during the reduction of carbon dioxide has been linked to their specific hydration structure at the interface.^509^ There are, in fact, numerous vibrational bands that can be used to follow electro-sorption processes, such as competing adsorption of CN^–^ and methanol at Pt electrodes.^510^ While these potential-induced chemisorption processes obviously involve oxidation/reduction processes, we categorized them as electro-sorption, with a strong focus on the electrode-adsorbate interaction. The vibrational studies with a focus on the electrochemistry are reviewed in the next subsection.

#### 4.5.3 Electrochemical Processes

Vibrational spectroscopy has provided detailed insights into electrochemical conversions by elucidating electrochemical reaction intermediates directly on the surface of the electrodes. For instance, the distinct vibrational coupling patterns of flavin mononucleotide have been resolved at the electrode,^511^ or the dynamics of a radical anion of a chromium complex attached to the electrode have been directly observed using 2D-IR.^512^ In addition to these examples of larger molecules at electrodes, many studies have focused on the oxidation or reduction of small molecules. A straightforward example is the oxidation of CO, which is readily chemisorbed on metal interfaces. Already a quarter of a century ago, SFG spectra hinted at intermediates at the electrode on the path from CO to CO_2_ in water/acetonitrile mixtures.^513^ In recent years, spectroscopic studies have focused on understanding how electrolytes, e.g., room-temperature ionic liquids, can steer the activity and selectivity of the catalytic CO oxidation. From the potentials at which oxidation of CO and other electrolyte components are vibrationally detected, it has been proposed that the nature of the ionic liquid electrolyte largely influences the availability of water molecules at platinum electrodes, which is strongly correlated with the oxidation overpotential.^514^

From a technological perspective, the inverse process, CO_2_ reduction, is highly relevant and has therefore received substantial attention from vibrational spectroscopists. However, in aqueous media, the electrochemical reduction of CO_2_ always competes with the reduction of water when protons are present. SFG spectroscopy has visualized proton sources (water or HCO_3_^–^) directly at the electrode interface via their distinct vibrational signatures, thereby contributing to elucidating the relevance of these competing reaction pathways.^515^ Also, for CO_2_ reduction, water-containing ionic liquids can provide a platform for highly efficient CO_2_ reduction, the molecular origin of which has been elucidated using SFG.^516^ In particular, SFG experiments have found that the high efficiency of this electroreduction is accompanied by a potential-induced structural transition of the ionic liquid. This structural transition in the ionic liquid has been sensed by the nonresonant SFG signals. From the electric-field-induced shift of adsorbed CO, the structural transition in the ionic liquid has been linked to a marked enhancement of the local EDL fields. Interestingly, these transitions occur exactly at the onset potential for CO_2_ reduction, which seems to be the key to understanding the high efficiency of this process.^516^ Furthermore, the Stark shift of in situ generated CO at gold electrodes in the presence of differently sized electrolyte cations has suggested that the electric field strength sensed by these CO molecules (i.e., the EDL field due to the Stern layer) does not correlate with the kinetics of the CO_2_ reduction. Conversely, Onsager reaction fields—electric fields imposed on the reacting molecule by the molecules in its immediate vicinity—have been suggested to be decisive for the reaction rate reduction.^517^ The authors of these studies have also noted that probing the vibrational signatures of in situ-generated CO warrants the selection of catalytically active sites at the metal electrode.^517^ Such catalytically active sites can also be introduced at the electrodes: Re-complexes have been shown to efficiently catalyze CO_2_ reduction. Using 2D-IR spectroscopy, the distinct dynamics of the catalyst and the reduced catalyst have been observed.^518^ These experiments have highlighted that the distinct dynamics of these catalytic intermediates may offer opportunities to identify electrochemical intermediates that are otherwise difficult to detect, but are important for mechanistic understanding.

We close this section by noting that while the hydrogen evolution reaction is much more straightforward to realize electrochemically, it is much more difficult to probe spectroscopically. Spectroscopic detection of chemisorbed hydrogen is experimentally challenging,^519^ and other intermediates or products lack distinct vibrational signatures, thereby preventing detailed insights from molecular spectroscopy. Nevertheless, the hydrogen evolution reaction consumes protons, which can locally alter the pH. This change in pH has been detected by SFG spectroscopy using a graphene electrode on a CaF_2_ substrate via the pH-induced alteration in the vibrational response of water in contact with the substrate.^500^ The consumption of protons has, in fact, been found at surprisingly moderate potentials, typically assigned to the capacitive regime, highlighting the sensitivity of this experimental setup.^500^ Similar to the CO_2_ reduction, catalytically active sites for the hydrogen evolution reaction based on Ir complexes may also provide detailed mechanistic insights in the future. The electrocatalytic intermediates of such catalysts contain Ir-H bonds, which can be readily detected spectroscopically, and their dynamics can be assessed with 2D-IR.^520^

### 4.6 Conclusions and Outlook

In summary, ultrafast nonlinear vibrational spectroscopies at solid–liquid interfaces have progressed from qualitative, continuum-based descriptions to providing molecular-level insights into hydration, ion adsorption and desorption, double-layer formation, and chemical reactivity across oxides, ionic minerals, metals, and low-dimensional materials. With phase-resolved methods, the description of interfaces has significantly advanced, yet studies are almost exclusively limited to a single molecular species at the interface. With the development of truly broadband spectroscopic techniques,^521,522^ however, multiple molecular species can be vibrationally detected, which has the potential to provide a fully comprehensive picture of hydration of interfaces, EDL formation, and also chemical reactivity, as, e.g., ions, solvents, adsorbates, and reaction products can be detected simultaneously. Additionally, methods that add depth information^523,524^ or independent observables^439^ provide the opportunity to really deepen our understanding of the discussed interfaces at the molecular level. Furthermore, methodological advances will make time-resolved and multidimensional measurements, electrochemical studies, and in operando experiments more broadly accessible, enabling molecular-level insight into solid–liquid interfaces to guide the design of catalysts, energy-conversion devices, and functional surfaces.

## 5 Nanoconfinement and Complex Architectures

### 5.1 Introduction and Overview

Electrolyte solutions under nanoscopic confinement occur in a wide range of natural and technological settings, including biological channels,^525,526^ ion-exchange membranes,^527,528^ porous materials,^529–531^ and Ångström-scale slit pores.^532–535^ Here, the emphasis is on how confinement modifies water structure, ion solvation, dynamics, and reactivity beyond the interfacial concepts introduced above.

The study of the ultrafast dynamics of electrolyte solutions under nanoconfinement is still in its infancy. As in many other areas, progress in the field has been driven to a large extent by the availability of suitable materials and models. As a consequence, the literature remains concentrated on a small number of popular systems studied by only a few groups, and unlike in other parts of this Review, systematic investigations across a broad range of confined electrolytes and host materials are still lacking. Many studies focused primarily on electrolyte solutions confined in zeolites^357,536,537^ and metal–organic frameworks^538^ followed more recently by carbon nanotubes and Ångström-scale two-dimensional slit pores,^539^ reflecting the growing ability to control confinement dimensions and geometry with high precision. At the same time, and largely independently, related questions about water structure, ion solvation, and dynamics under confinement have been explored in soft and biological materials, including membranes,^527^ reverse micelles,^540^ and ion channels.^525^

Our understanding of the structure and dynamics of confined pure water is just beginning to consolidate. While establishing this baseline is a necessary first step before addressing electrolyte effects, our knowledge of confined electrolyte solutions remains limited. In this section, we focus on the insights provided by ultrafast vibrational spectroscopies into the structure, dynamics, and reactivity of electrolyte solutions under nanoscopic confinement. Transport properties, such as ionic transport, hydrodynamic flow, or friction, generally involve different time and length scales and are thus not comprehensively covered, they have been reviewed elsewhere.^532,541^

### 5.2 Solid Confinement

#### 5.2.1 Distinguishing Nanoconfinement from Interfacial and Core Regions

A central question in the study of electrolyte solutions under nanoconfinement is to what extent the behavior of the confined system can be described as a combination of interfacial and core regions and whether the latter can still be regarded as bulk-like. If this picture holds, then many apparent confinement effects do not reflect genuinely new emergent properties but rather the increasing relative weight of the interfacial region as the size of the confining environment decreases. Ultrafast vibrational spectroscopies are particularly well-suited to address this question because they directly probe the hydrogen-bond network of water, its heterogeneity, and how electrolytes perturb these local environments.

This issue has been examined extensively in silica pores and reverse micelles.^542^ Water dynamics in reverse micelles has been addressed by 2D-IR spectroscopy of the water OH stretch vibrations^543,544^ and interfacial phosphate groups.^545^ Since the latter is reviewed in another contribution to this edition, we focus here primarily on silica. Nanoporous and mesoporous silica can serve as simple models of porous silica environments that are relevant to both natural and technological settings. In contrast to softer confining systems, such as reverse micelles, nanoscale silica pores provide a rigid framework, often with an interconnected pore geometry. Two widely studied examples are MCM-41 and related materials such as SBA-15, whose structures consist of parallel arrays of uniform cylindrical channels with tunable diameters, typically ranging from about 1.5 to 10 nm or even larger depending on the synthesis conditions.^546,547^ These materials are commonly synthesized in aqueous solution via sol–gel methods or by templating with cylindrical micelles, which generally yield pore surfaces terminated with silanol groups and thus hydrophilic interfaces. As described in the previous section, extended silica interfaces also contain siloxane bridges, and silanols can exist in a variety of local environments, interacting with nearby siloxane oxygens, other silanols, or water molecules confined within the pores. Because the silanol density, distribution, and surface roughness depend sensitively on the synthesis procedure, the resulting interfaces are often chemically heterogeneous. At the same time, these surface groups can be chemically modified, for example, by replacing silanols with Si-OR moieties, to produce more hydrophobic interfaces. This combination of structural regularity, tunable pore size, and adjustable surface chemistry has made mesoporous silica a widely used platform for investigating how confinement alters the structure and ultrafast dynamics of water and aqueous solutions.

The behavior of confined liquids is often described using a core–shell model.^542^ In this framework, a given observable is assumed to take one characteristic value in the near-surface shell, and another in the pore interior, with the core often approximated as bulk-like. A representative example is provided by ultrafast infrared studies of selenocyanate (SeCN^–^) dissolved in hydrated silica mesopores with a diameter of approximately 2.4 nm, in which orientational relaxation and spectral diffusion dynamics were compared between bulk and confined environments.^357^ In these experiments, a slow-decay component absent in the bulk liquid was observed in both measurements, suggesting that confinement significantly perturbs the underlying hydrogen-bond dynamics. MD simulations further indicated that orientational dynamics become progressively faster with increasing distance from the pore wall, approximately following an exponential decay, whereas spectral diffusion appears to exhibit a more abrupt crossover from slower to faster dynamics at approximately 6 Å from the interface, consistent with MD predictions.^548,549^ A subsequent study extended this analysis to larger silica pores of up to approximately 5.4 nm and found that the distance dependence of the dynamics remains similar over this size range, supporting the interpretation that the average dynamics in different pore sizes may be described, at least as a first approximation, as a population-weighted sum of the same interfacial and core-like contributions.^344^ This view is broadly in agreement with temperature-dependent Raman studies of larger silica nanopores in the 4–7 nm range,^550^ as well as with reports of two water populations, near-surface and core water, in other confined systems such as zeolites^536^ and reverse micelles.^551^ At the same time, more detailed analyses of water dynamics using atomistic simulations suggest that the core–shell picture should be regarded as an effective approximation since the shell region itself is highly heterogeneous and cannot always be represented as a single well-defined dynamical state.^552^

Nanoconfined environments formed between extended planar interfaces have also been proposed to be accessible by VSFG, provided that the two confining materials are distinct such that the vibrational responses from the opposing interfaces do not fully cancel each other.^553^ More recently, HD-VSFG has been used to probe water confined between CaF_2_ and graphene, showing that for confinement widths of approximately 1 nm, corresponding to about three water layers, the response of the confined water can be quantitatively described as the sum of the individual interfacial water signals from the CaF_2_/water and water/graphene interfaces^539^ (Figure 16). For weaker confinement, no significant changes in the signal were observed, consistent with the picture that the water molecules in the interior remain, on average, isotropically oriented, as in the bulk. Upon further reduction of the confinement, however, deviations from this additive description begin to emerge, indicating the onset of genuine confinement effects that cannot be captured as a simple superposition of the two isolated interfaces. In the same study, similar conclusions were reported for the SiO_2_/water/graphene system.

**Figure 16:**
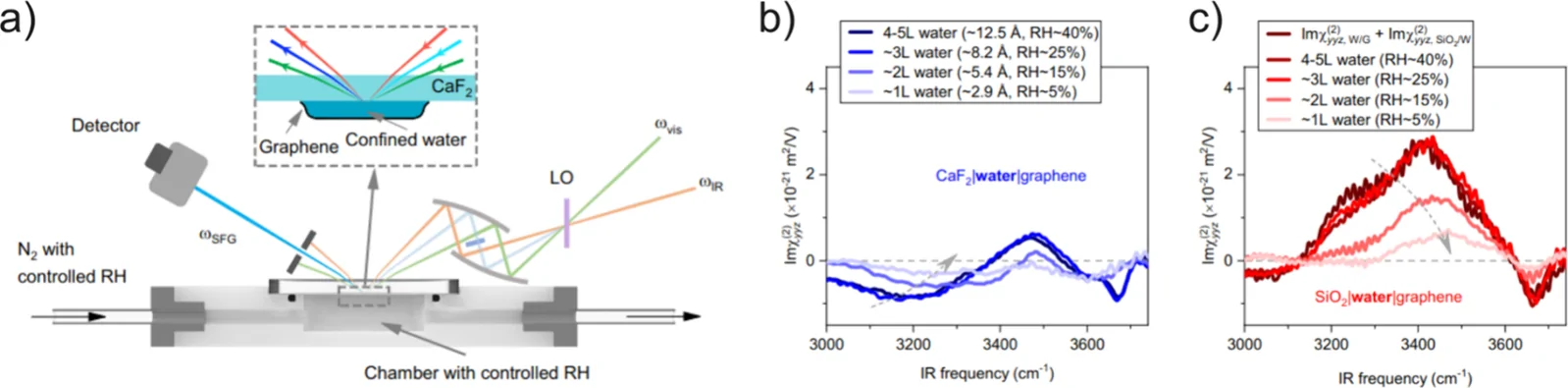
(a) Experimental setup employed to measure the VSFG spectra of nanoconfined water. (b) Evolution of VSFG spectra of water confined between graphene and CaF_2,_ showing how the vibrational response evolves with decreasing water-layer thickness and thereby reveals the growing effect of confinement on water. (c) Same as (b) but for water confined between graphene and SiO_2_. Adapted from Wang et al., *Nat. Commun.* 2025, 16, 7288 under the terms of the Creative Commons CC BY 4.0 license.^539^ Copyright 2025 The Authors.

The effect of MgCl_2_ was also investigated for aqueous magnesium chloride solutions of 2.5–4.2 m confined in mesoporous silica with a pore diameter of 2.8 nm, again using a core–shell two-state model and selenocyanate as a vibrational probe. In these studies, increasing salt concentration led to a slowing down of both the rotational dynamics and spectral diffusion of the probe under confinement,^537^ and this effect was more pronounced than in the corresponding bulk solution.^554^ For aqueous MgCl_2_ solutions, the crossover from shell-like to core-like dynamics is sharper, shifted to slightly larger distances, and independent of the salt concentration (Figure 17). The authors attributed these confinement-induced effects to the strong electrostatic interactions between the cations and the charged silica walls, underscoring the importance of the strength of solute–surface interactions in determining confined dynamics. More generally, the different responses of water-associated and cation-associated dynamics further illustrate that confinement effects depend sensitively on the nature of the confined species. MD simulations for sodium halide salts confirmed the slowing down of the rotational and spectral diffusion. While the former is anion-independent, the latter shows an increase from chloride to iodide.^555^

**Figure 17:**
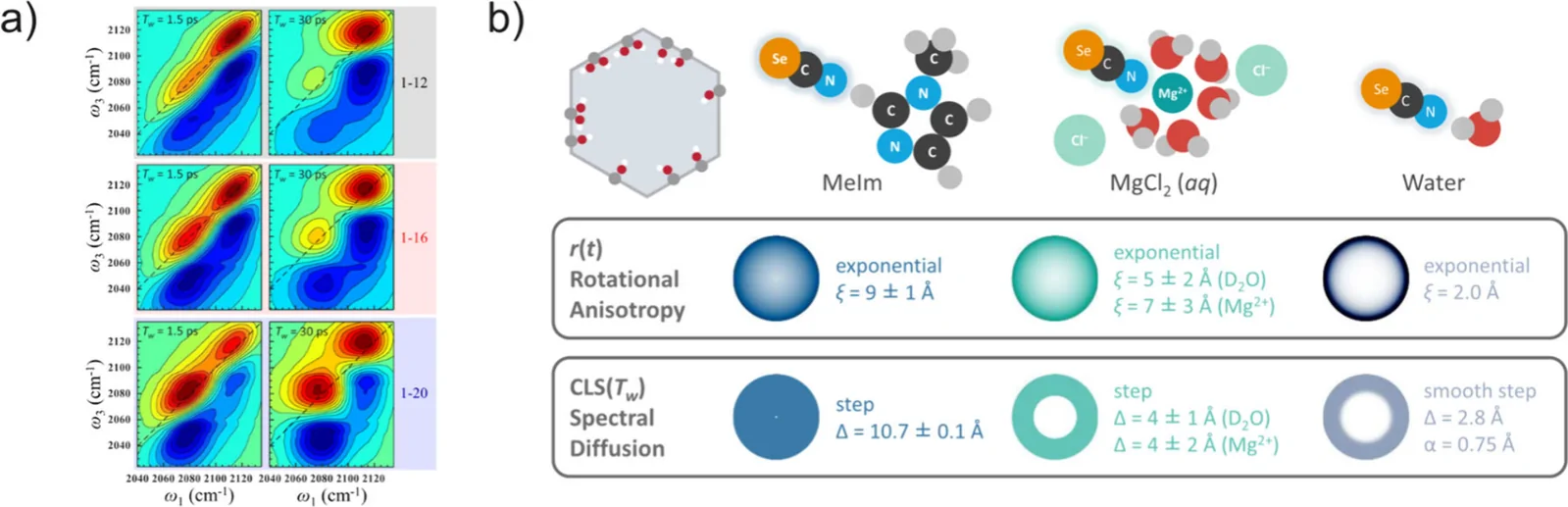
(a) 2D-IR spectroscopy of the CN stretch of SeCN^–^ in aqueous MgCl_2_ solutions of varying concentration confined within silica mesopores. Changes in rotational anisotropy and spectral diffusion reveal distinct core and interfacial shell regions in nanoconfined water. (b) Comparison of two-state model results describing the dynamics of SeCN^–^ in different nanoconfined solutions. Adapted with permission from Hung et al., *J. Phys. Chem. B* 2024, 128, 5513–5527.^537^ Copyright 2024 American Chemical Society.

#### 5.2.2 Acid/Base Equilibria under Nanoconfinement

In this section, we continue discussing changes in the acid/base equilibria, including the p*K*_a_ of molecules and water under nanoconfinement as well as changes in the acid–base properties of surface sites. These equilibria are ultimately determined by the self-ionization of water described by the equilibrium constant *K*_w_. Understanding this process in detail, which factors modify *K*_w_ and by how much, is essential because it affects the p*K*_a_ and reactivity of molecules as well as the protonation state of functional groups at the interface.

Despite its central importance, *K*_w_ has not yet been measured experimentally for nanoconfined water. Theoretically, the behavior of *K*_w_ in water confined between two-dimensional materials has instead been investigated extensively, mainly through MD simulations. At first glance, these studies have reported contradictory results, ranging from enhanced self-ionization,^556^ to inhibited dissociation,^557,558^ to an almost bulk-insensitive response.^559^ More recently, it has been suggested that part of this disagreement may arise from differences in the bulk reference states used for comparison, as well as from equilibration protocols that yield different effective pressures within the nanoslit.^560,561^ Thus, if considering the case of a nanoslit in equilibrium with a bulk water reservoir, the latest results show no appreciable change in the p*K*_w_. The p*K*_w_ depends sensitively on the details of the water-wall interaction—including whether ionic species undergo physisorption or chemisorption at the wall^558,560^ and much less is known for hydrophilic aprotic walls, including both organic interfaces and inorganic surfaces such as certain metal oxides, halides, nitrides, or chalcogenides, where water-wall interactions may be strong even in the absence of directly proton-donating surface groups. Despite this disagreement, it is generally accepted that thicker films recover bulk-like behavior within 1.5–2 nm,^559^ and that the molecular solvation structures of the proton and hydroxide appear more robust. For example, at graphene walls, the proton retains three strong donor interactions and one weak acceptor interaction, while hydroxide cannot adopt its usual hypercoordinated shell and is consequently destabilized.^557,559–561^

Based on these studies, the common assumption that the p*K*_w_ of water remains largely unchanged for confinement sizes above about 1.5–2 nm appears to be valid. Since, beyond the 2D slit-pore setups described above, most water-accessible confining materials rarely exhibit dimensions smaller than the characteristic dimensions of the surface sites, the p*K*_a_ values of surface sites in these systems are unlikely to be strongly perturbed by confinement-induced changes in the p*K*_w_ of water. Instead, the acid–base chemistry of hydrated confined environments should be determined mainly by the intrinsic acidity of interfacial sites and by their local solvation structure. This is particularly relevant for materials containing Brønsted acid sites (BAS). Zeolites are a particularly prominent example; due to their environmentally benign nature, they are widely used in catalysis,^562,563^ separations, and ion transport.^564^ In particular, in aluminosilicate zeolites, substitution of Si by Al generates negatively charged framework sites that are compensated by protons, giving rise to strong BAS. Because water can strongly affect the structure, protonation state, and reactivity of these sites, a microscopic description of hydrated zeolites is essential for understanding their molecular mechanisms and guiding the rational design of materials.

IR has been extensively used to characterize the hydration of Brønsted acid sites inside zeolites. This technique is particularly useful because the OH stretching frequency is highly sensitive to the local hydrogen-bonding environment and can therefore report on both water adsorption and proton transfer. In hydrated HZSM-5, the IR spectrum is extremely broad and displays an asymmetric low-frequency wing, with features centered near 3650 cm^–1^, a shoulder around 3600 cm^–1^, and broader bands near 3400 and 3200 cm^–1^ (Figure 18).^467^ The band at 3400 cm^–1^ is assigned to hydrogen-bonded water OH stretches; the band around 3200 cm^–1^ is usually assigned to a Fermi resonance between the water OH stretch and the overtone of the HOH bending mode. The feature near 3650 cm^–1^ is consistent with terminal, or free, OH vibrations, in line with assignments previously reported for the water–air interface^565^ and for water confined in carbon nanotubes.^546^

**Figure 18:**
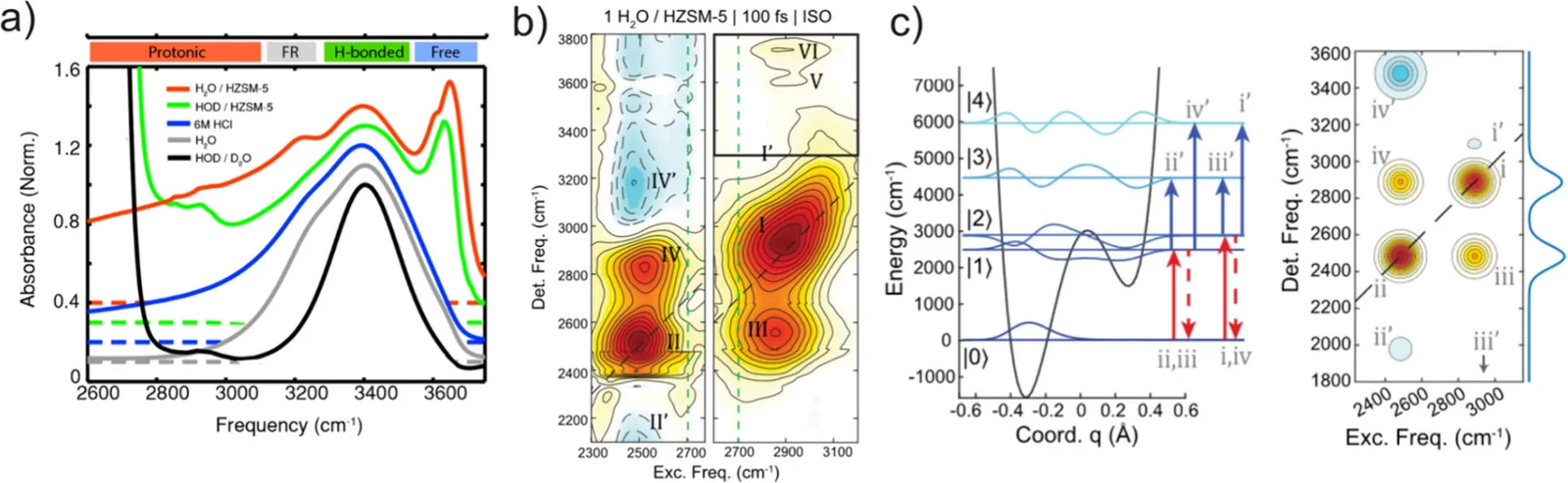
Infrared signatures of water adsorption at Brønsted acid sites in HZSM-5 and their interpretation from two-dimensional spectroscopy. (a) FTIR spectra of hydrated HZSM-5 showing the broad and structured response associated with water adsorption at the acid site, including contributions from protonic, Fermi-resonant, hydrogen-bonded, and free OH vibrations.^467^ (b) 2D-IR measurements and (c) corresponding model analysis used to identify the underlying proton-transfer potential energy surface of the Brønsted acid site–water complex. Comparison with theoretical models supports an anharmonic double-well proton-transfer picture rather than a simple Fermi-resonance assignment for the observed doublet.^464^ Adapted with permission from Hack et al., *J. Am. Chem. Soc.* 2021, 143, 10203–10213 ^467^ and Hack et al., *J. Phys. Chem. B* 2023, 127, 11054–11063.^464^ Copyright 2021 and 2023 American Chemical Society.

However, the strong broadening and overlap of the linear IR bands limit the structural detail that can be extracted. This difficulty is further compounded by the coexistence of different hydration states at a given average water loading, which makes the quantitative analysis of protonation equilibria challenging. These limitations motivated the use of time-resolved vibrational spectroscopy. Some of the earliest time-resolved vibrational studies in zeolites focused on the lifetimes of OD modes of water adsorbed at Brønsted acid sites in deuterated mordenite. Using tunable picosecond infrared pump–probe spectroscopy, Domen et al. observed a short-lived transient absorption feature with a lifetime of about 15 ps, assigned to a newly formed unstable surface species.^566^ In a follow-up study, they showed that the vibrational lifetime depends strongly on the local environment of the adsorbed D_2_O molecule. In particular, the more weakly interacting OD group displayed a much longer lifetime of 43 ± 5 ps, whereas the OD group strongly hydrogen-bonded to the zeolite surface relaxed on a time scale of about 15 ps, which was interpreted as evidence for the formation of a short-lived surface species, possibly D_3_O^+^.^567^ Fujino et al. later supported this picture by assigning the distinct dynamical components to water molecules adsorbed at acidic OD sites in the main channels and small cavities of the zeolite framework.^568^

Related work confirmed that the OD vibrational lifetime in mordenite is frequency-dependent, consistent with variations in hydrogen bonding among the deuterated hydroxyl species present in the zeolite.^569^ Their temperature-dependent measurements further showed that the OD absorption band redshifts and broadens with increasing temperature, reflecting coupling between the local hydroxyl stretch and thermally populated low-frequency lattice modes. They concluded that, after IR excitation, the excess vibrational energy is rapidly redistributed into delocalized framework modes, so that the zeolite lattice strongly influences relaxation of the excited hydroxyl. This picture was refined by further measurements of the lifetime of the OH stretching mode of a water molecule hydrogen-bonded to a Brønsted acidic hydroxyl group in mordenite over a range of temperatures.^568^ The unusually strong temperature dependence they observed was attributed to a relaxation pathway involving the excitation of an adsorbate librational mode together with two bulk phonons of the zeolite.^568^

A recent advance in this area was in situ femtosecond 2D-IR spectroscopy, which enabled the distinction of several types of hydroxyl species in zeolites and the quantification of their relative abundances.^359^ In particular, Brønsted acid sites, silanol defects, and water of hydration were shown to display distinct 2D-IR signatures. Brønsted acid hydroxyls exhibited characteristically inhomogeneously broadened band shapes, whereas water of hydration and partially hydrogen-bonded silanol groups gave rise to off-diagonal cross peaks. Hydrogen-bonded silanol groups associated with vacancy defects, that is, silanol nests, displayed yet another distinctive signature, including picosecond anisotropy decay and spectral diffusion. Notably, these ultrafast dynamics were found to be essentially identical in ZSM-5 and silicalite.

Beyond enabling this spectral separation, the 2D-IR measurements were combined with a spectrophotometric concentration-ratio analysis specific to pump–probe and 2D-IR methods, allowing the relative population of silanol-nest deuteroxyl groups and Brønsted ZOD groups to be quantified without requiring independent knowledge of their absorption coefficients. For the zeolite ZSM-5 sample studied, the silanol-nest hydroxyls were found to be present in proportions comparable to those of the Brønsted hydroxyls.^359^ This result highlights that defect-associated silanol species can constitute a substantial fraction of the hydroxyl population and should therefore be explicitly included in both the interpretation of vibrational spectra and atomistic models of these materials.

Although these observables did not provide conclusive structural insight, they highlighted the need for more detailed time-resolved spectroscopic approaches. 2D-IR measurements^467^ showed that the water speciation statistics are dominated by molecules donating one or zero H-bonds, indicating short, branched hydrogen-bonding networks that terminate after only two to four water molecules, consistent with the hydrophobic character of HZSM-5 pores.

Subsequent work focused more directly on the protonation state of the Brønsted acid site as a function of hydration. Experiments combining linear IR and 2D-IR spectroscopy and analyzing the evolution of the doublet peaks near 2500 and 2850 cm^–1^ associated with the BAS as a function of hydration showed that a single water molecule is insufficient to fully deprotonate the acid site, whereas two or more water molecules are required to induce deprotonation.^465^ By modeling the filling of the pores using the Brunauer–Emmett–Teller theory, the authors demonstrated that even when the average number of water molecules per BAS is two or more, there remains a distribution of pore fillings, such that some pores still contain only one water molecule. This rationalizes the persistence of the doublet even at high water loading. A further study focused specifically on the origin of the doublet and, by comparing the measured spectra with several theoretical models, provided strong evidence that this feature is better explained by an anharmonic double-well proton-transfer potential than by a stretch–bend Fermi resonance picture.^464^

A related but structurally distinct class of porous materials is represented by metal–organic frameworks (MOFs), porous molecular architectures in which metal clusters are bridged by organic linkers to form three-dimensional coordination polymers with nanometer-sized cavities. MOFs present a unique combination of structural elasticity, spatial regularity, and tunable surface chemistry—where the balance of hydrophobic and hydrophilic interactions can be systematically varied by changing the metal nodes or organic linkers—that makes them a particularly versatile class of porous materials. A few studies have employed ultrafast vibrational spectroscopy to investigate molecular dynamics within these frameworks. Computational 2D-IR spectroscopy of MIL-53­(Cr) revealed that at low water loadings, molecules are pinned to the framework and undergo rapid H-bond rearrangement faster than in bulk water, whereas at higher loadings the growing water–water interactions progressively suppress reorientational dynamics, such that spectral diffusion slows significantly. Water relaxation remains slower than in the bulk even when pores are fully occupied.^538^ In contrast, ultrafast IR measurements on Ni_2_Cl_2_BTDD, a MOF with larger pores of approximately 2.2 nm, showed that the confined H-bond network retains the ability to rearrange on a picosecond time scale resembling bulk dynamics, distinguishing this system from other confined geometries where such rearrangements are hindered.^570^ 2D-IR spectroscopy with pulse-shaping techniques has been shown to probe ultrafast structural fluctuations of the MOF scaffold itself,^571^ suggesting that combining framework dynamics with water dynamics measurements could provide a more complete picture of how structural elasticity and confinement jointly couple and ultimately govern the molecular behavior in these systems (Figure 19).

**Figure 19:**
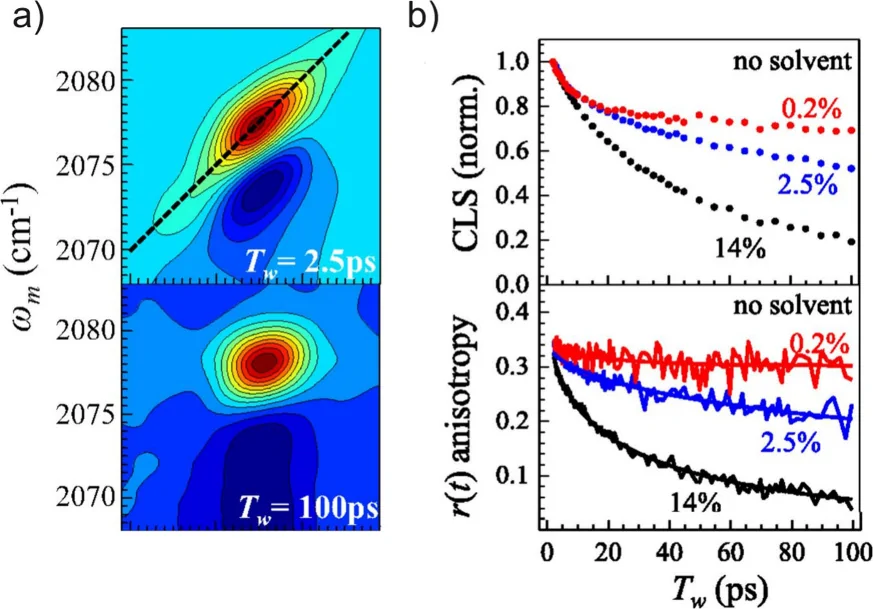
Ultrafast structural dynamics of a functionalized UiO-66 metal–organic framework probed by 2D-IR spectroscopy of the symmetric CO stretching mode (∼2077 cm^–1^) of a FeFe­(CO)_6_ complex incorporated as a framework linker. (a) 2D-IR spectra at waiting times of *T*_w_ (= *τ*_2_) = 2.5 and 100 ps. At early *T*_w_, the 0–1 band is elongated along the diagonal (dashed line), reflecting inhomogeneous broadening from the distribution of framework environments; by 100 ps the band has become nearly round, indicating essentially complete spectral diffusion driven by structural fluctuations of the framework. (b) Normalized center line slope (CLS) decays (top) and pump–probe anisotropy decays *r*(*t*) (bottom) for probe loadings of 14%, 2.5%, and 0.2% in the solvent-free MOF. The pronounced concentration dependence of both observables identifies Förster vibrational excitation transfer as the dominant contribution at higher loadings, whereas at 0.2% loading excitation transfer is negligible and the CLS decay reports purely on structural spectral diffusion of the framework. Adapted with permission from Nishida et al., *Proc. Natl. Acad. Sci. U.S.A.* 2014, 111, 18442–18447.^571^ Copyright 2014 National Academy of Sciences.

### 5.3 Soft Confinement in Polymer Membranes

While the preceding sections addressed confinement within rigid, well-defined geometries, a distinct regime arises when the confining walls are themselves flexible and chemically heterogeneous—as in polymer membranes and biological channels. Ultrafast vibrational spectroscopy is particularly well suited to probe these environments, as it can directly report on water structure and hydrogen-bond dynamics within such disordered matrices. Two important examples are perfluorinated sulfonic-acid membranes and biological ion channels; since the latter have been thoroughly reviewed elsewhere,^572,573^ we focus in the following on perfluorinated sulfonic-acid membranes.

Perfluorinated sulfonic acid ionomers are a class of ion-conducting polymers known for their high ionic conductivity together with excellent chemical and mechanical stability.^528^ Because of this combination of properties, they are widely used as solid electrolytes in electrochemical technologies, most notably as proton-exchange membranes in polymer–electrolyte fuel cells and as sodium-ion conductors in the chlor-alkali industry. Nafion, the first commercially available member of this family, remains the most widely studied example. Its structure combines a nonpolar fluorinated backbone with polar side chains terminated with sulfonic acid groups. This strong polarity contrast drives nanoscale phase separation into hydrophobic and hydrophilic domains, with the latter hosting water molecules and mobile counterions. Considerable effort has been devoted to determining the morphology of hydrated domains in Nafion and relating this morphology to the behavior of confined water. The classical model proposed by Hsu and Gierke describes Nafion as a network of spherical clusters, roughly 4 nm in diameter, connected by narrower channels ∼ 1 nm wide.^574^ With the accumulation of experimental data and advances in simulation methods, a broader range of structural motifs has been proposed.^527^ For example, X-ray studies have supported a picture in which fibrillar hydrophobic polymer aggregates organize the hydrophilic side chains into irregular water-filled channels that expand or contract with hydration.^575^

Upon hydration, the hydrophilic domains swell and progressively form an interconnected network of channels across the membrane. Above the percolation threshold, these water-filled pathways enable ion transport through the material. In protonated Nafion, hydration also mobilizes the acidic protons associated with the sulfonic groups, a process that is central to the membrane function in fuel cells. As a result, proton transport depends strongly on water content.^576^ More broadly, the performance of polymer–electrolyte membranes such as Nafion is governed by the properties of the confined water, making its structure and dynamics key to understanding proton transfer and transport.

Previous infrared studies of water in Nafion often relied on pure H_2_O, whose spectrum is highly congested because of the large number of overlapping vibrational bands.^577^ To obtain a clearer view of the water dynamics, later work used the O-D stretch of dilute HOD in H_2_O as a local vibrational probe and followed its relaxation with ultrafast infrared pump–probe spectroscopy.^578^ These measurements provided evidence for two distinct water ensembles in Nafion and showed that the orientational dynamics of confined water are significantly slower than those in bulk water, with the slowdown becoming more pronounced at lower hydration. Wavelength-dependent vibrational lifetime measurements at different water contents further showed that the properties of these two ensembles evolve with hydration, indicating that the hydrophilic domains do not retain a fixed interfacial structure. Instead, the side chains and sulfonate groups appear to be sufficiently flexible to rearrange as the membrane swells. Related infrared and fluorescence studies over hydration levels ranging from dry to fully hydrated further highlighted the strong coupling between the water structure and dynamics in the hydrophilic regions of Nafion.^579^ Similarly, strong coupling between interfacial water population and water hydrogen-bond network dynamics has been reported in swollen lipidic mesophases.^580^

A more detailed dynamical picture was later obtained from polarization-selective pump–probe and two-dimensional infrared spectroscopies on fully hydrated Nafion membranes.^581^ In these experiments, the acidic protons were exchanged for sodium ions to avoid the extremely broad hydronium absorption, thereby isolating the dynamics of water itself. A two-component analysis of the population and orientational dynamics confirmed the presence of core and interfacial water subensembles within the hydrophilic domains. The analysis of orientational dynamics showed that water in the core reorients on nearly bulk-like time scales (∼2.8 ps), whereas interfacial water is much slower (∼21 ps), likely due to the interactions with the sulfonate groups. Two-dimensional infrared measurements also revealed spectral diffusion, showing that structural rearrangements at the interface are considerably slower than in bulk water.^581^ Comparison of the population lifetimes and reorientation time scales with those of aerosol-OT reverse micelles and bulk water suggests that the traditional spherical-cluster morphology, while widely used,^582^ provides an oversimplified description of Nafion. More specifically, some dynamical observables resemble those of smaller reverse micelles, whereas others are closer to larger ones, indicating that Nafion cannot be described adequately as a single-size reverse micelle (Table 1), and morphologies with less restricted confinement, such as channel-like or cylindrical domains, are more consistent with the observed dynamics.^581^ A complementary perspective was provided by coherent Raman spectroscopy, which was used to quantify in situ the diffusivities of bulk-like and interfacial water in five different PFSA membranes.^583^ Despite the chemical diversity of the membranes, the total water diffusivity could be described as a linear combination of the diffusivities of these two water populations. Interestingly, interfacial water was found to diffuse faster than bulk-like water.

**Table 1:** Summary of vibrational population and orientational dynamics of dilute HOD in Nafion, aerosol reverse micelles, and bulk water. Here, *τ*_1_ and *τ*_2_ correspond to the population lifetimes of the core and interfacial water populations, whereas *τ*_or,1_ and *τ*_or,2_ denote their orientational relaxation times, respectively. Reproduced with permission from Roget et al., *J. Phys. Chem. B* 2019, 123, 9408–9417.^581^ Copyright 2019 American Chemical Society.

System	***τ***_1_ (ps)	***τ***_2_ (ps)	***τ***_or,1_ (ps)	***τ***_or,2_ (ps)
Nafion	2.4 ± 0.2	5.9 ± 0.3	2.8 ± 0.2	21 ± 3
Reverse micelle (4 nm)	2.2 ± 0.2	5.3 ± 0.1	4.0 ± 0.2	26 ± 3
Reverse micelle (6 nm)	1.8	4.3 ± 0.2	2.6	18 ± 3
Bulk water	1.8 ± 0.1	–	2.6 ± 0.1	–

In water, excess protons can be transported either by the motion of hydronium-like species or by the transfer of the protonic charge along the hydrogen-bond network. The latter process, known as structural diffusion or the Grotthuss mechanism, is what gives protons their anomalously high mobility in aqueous environments. Because this mechanism depends on suitable solvation configurations and hydrogen bond rearrangements, it is closely tied to the dynamical fluctuations of the surrounding water structure.

Two limiting hydration structures are often used to describe the excess proton. In the Eigen motif, the proton is localized on a hydronium ion that is strongly hydrogen-bonded to three neighboring water molecules, forming an H_9_O_4_^+^ complex. In the Zundel motif, the proton is shared between two water molecules, forming an H_5_O_2_^+^ complex (cf. Section 2). Although the real hydrated proton continuously interconverts between such configurations, these idealized structures provide a useful language for discussing proton solvation. In Nafion, MD simulations suggest that the proton is more Zundel-like within the first hydration shell of the sulfonate group, while more Eigen-like configurations are favored farther away from the counterion.^584^ The balance between these two motifs is sensitive to the local hydration environment. Under minimal hydration, the conformation of the fluorocarbon backbone, the flexibility of the side chains, and the degree of aggregation of the sulfonic acid groups collectively control proton dissociation and the relative population of Zundel and Eigen configurations.^585,586^ As water content increases, solvent-separated Eigen ions begin to appear, and the dominant proton diffusion pathway shifts to a region approximately 4 Å away from the sulfonate groups. MD simulations further reveal that proton diffusion in Nafion is more complex than in bulk water, with hopping and vehicular contributions being anticorrelated rather than additive, highlighting how confinement can fundamentally alter transport mechanisms.^584^

Further insight into proton dynamics in Nafion across different hydration levels has been obtained using photoacids. Spry and co-workers showed that proton-transfer dynamics and equilibrium proton concentrations vary substantially across different regions of the water domains in Nafion.^587^ More specifically, measurements with the highly anionic probe 8-hydroxypyrene-1,3,6-trisulfonate (HPTS) indicated that this region behaves much like bulk water under well-hydrated conditions, namely for *λ* values above 12, where *λ* is the number of water molecules per sulfonate group. At high hydration, the proton-transfer kinetics of HPTS in Nafion were found to be nearly identical to those in bulk solution, and the proton concentrations in the center of the water pools were estimated to be 0.54 and 0.95 M for *λ* = 22 and 12, respectively. In contrast, measurements with rhodamine-6G suggested that the hydronium concentration near the water-interface region is considerably higher, reaching about 1.4 M.^587^

In other studies using similar probes, excited-state proton transfer and recombination were monitored by measuring the decay of photoacid fluorescence.^579^ The large negative charges of these photoacids preferentially place them near the center of the aqueous domains, away from the sulfonate-rich interface. Because proton dissociation from the excited probe and the subsequent charge separation depend on how efficiently the surrounding water solvates the released proton, the measured kinetics provide a sensitive probe of local hydrogen bond rearrangements. Complementary ultrafast infrared measurements of the O-D stretch of dilute HOD in H_2_O were used to characterize the local environment and H-bond dynamics of water in the hydrophilic regions of Nafion. Together, these measurements showed that both water reorientation and proton transfer over nanoscopic distances are markedly slowed as the membrane becomes less hydrated and that the extent of excited-state proton transfer is strongly suppressed at low hydration. This behavior is consistent with the picture that, at low water content, protons are more strongly associated with the sulfonate groups, and diffusion proceeds mainly through vehicular motion, whereas increasing hydration provides sufficient solvation and connectivity for structural diffusion to become more important. At the same time, more recent measurements across a series of perfluorosulfonic acid membranes at fully hydrated conditions found a positive correlation between proton conductivity and the apparent diffusivity of interfacial water.^583^ This points to a more active role for sulfonate groups than for simple counterions, a picture further supported by simulations of sulfonated graphene bilayers, which suggest that under optimal hydration conditions, the sulfonate end groups can enhance proton conductivity.^588^ Taken together, these studies suggest that core and interfacial water are dynamically coupled and that this coupling plays an important role in determining proton transport.

An especially striking example of how confinement modifies proton behavior in Nafion was provided by femtosecond mid-IR experiments showing that, in these nanochannels, single-quantum excitation of a proton vibration can directly trigger proton transfer at room temperature.^589^ Such vibration-driven chemistry is extremely uncommon in the condensed phase, where intermolecular interactions usually dissipate the vibrational energy before a reaction can occur. Polarization-resolved measurements indicated that excitation of an OH stretch in an Eigen-like proton environment induces a structural reorganization in which the initially excited hydrogen bond contracts and the proton charge becomes more localized along a special pair, leading to a more Zundel-like configuration. A subsequent study examined how this process depends on hydration.^590^ At low hydration, the excited proton vibration has a relatively long lifetime, with a vibrational relaxation time constant *T*_1_ of about 490 ± 40 fs at *λ* = 3, making vibration-induced proton transfer clearly observable. However, as the water content increases, *T*_1_ decreases to 230 ± 30 fs at *λ* = 16. Thus, with increasing hydration, a larger fraction of the excited Eigen states E_1_ relaxes to the locally hot ground state E_0_* instead of proceeding to the first excited Zundel state Z_1_ (Figure 20). As a consequence, the population of Z_1_ decreases, and vibration-induced proton transfer becomes less clearly observed at higher hydration levels. As a reference, in bulk water, the proton vibrational lifetime is only about 110 ± 20 fs, and no excitation-induced proton transfer is observed.^591^

**Figure 20:**
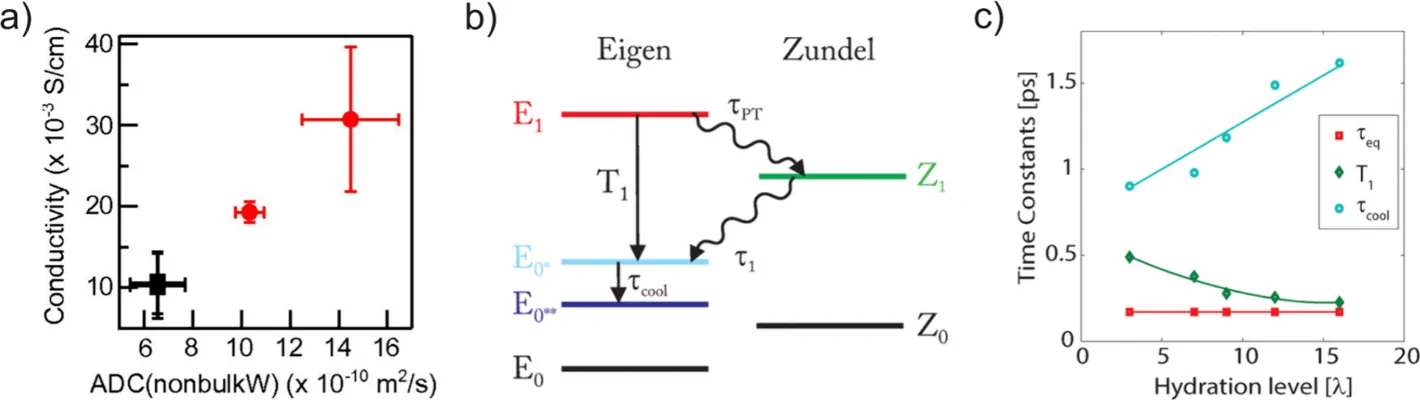
Role of interfacial water and proton vibrational dynamics in hydrated PFSA membranes. (a) Linear correlation between the apparent diffusion coefficient (ADC) of nonbulk interfacial water and through-plane proton conductivity, highlighting the contribution of interfacial water to transport. Black (red) symbols refer to extruded Aquivion (Nafion) membranes with short (long) side chains.^583^ (b) Kinetic model for IR-induced proton transfer between Eigen-like and Zundel-like configurations in Nafion and (c) associated hydration-dependent time constants.^590^ The decrease of the vibrational lifetime *T*_1_ with increasing hydration shows that faster relaxation increasingly competes with proton transfer, making excitation-induced proton transfer less probable at higher water content. Adapted with permission from the authors from Ling et al., *Proc. Natl. Acad. Sci. U.S.A.* 2019, 116, 8715–8720, ref 583, published under the terms of the Creative Commons CC BY-NC-ND 4.0 license. Copyright 2019 The Authors. Adapted with permission from Liu and Bakker, *J. Phys. Chem. B* 2015, 119, 2628–2637, ref 590. Copyright 2015 American Chemical Society.

### 5.4 Conclusions and Outlook

Despite substantial progress, an understanding of electrolyte solutions under nanoconfinement remains in its early stages. While the structure and dynamics of confined water are now comparatively well-characterized, much less is known about the behavior of ions, their solvation environments, ion pairing, and their coupling to the confined hydrogen-bond network. This imbalance is not unusual: as in several areas, the field has first focused on establishing a molecular picture of water before moving on to the more complex case of electrolyte solutions, and it is likely that future work will increasingly shift in this direction. Addressing these questions will benefit from the continued development of model systems with increasingly well-defined geometries in which confinement dimensions, topology, and surface chemistry can be tuned independently and with high precision.

From a methodological perspective, time-resolved IR and 2D-IR spectroscopies appear particularly well suited for probing three-dimensionally confined environments such as pores, zeolites, and MOFs, whereas it remains an open question to what extent SFG may be better suited for one- and two-dimensional confinement geometries, where broken symmetry at interfaces can be exploited more directly. More broadly, this field has been strongly shaped by theory, partly because many confined systems are accessible to atomistic simulation and partly because simulations have predicted a wide range of unusual, and sometimes exotic, behaviors. Yet, many of these predictions still lack clear experimental verification. For instance, although vibrational spectroscopies cannot directly determine quantities such as p*K*_w_, they can access related observables by tracking the spectroscopic signatures of protonated and deprotonated species, thereby probing effective p*K*_a_ shifts under confinement. More generally, while water dynamics under confinement are now comparatively well characterized, the dynamics of ions—their solvation environments, mobility, and coupling to the confined hydrogen-bond network—remain much less explored experimentally. In this way, ultrafast vibrational spectroscopy is particularly well positioned to bridge the gap between theory and experiment by delivering molecular-level benchmarks of structure, heterogeneity, and dynamics in nanoconfined electrolyte solutions.

## 6 Outlook

Ultrafast nonlinear vibrational spectroscopy has transformed the study of aqueous solutions at surfaces and in heterogeneous environments from a largely static, phenomenological description to one in which molecular structure, heterogeneity, and dynamics can be interrogated on their native femtosecond-to-picosecond timescales. Across bulk reference systems, soft interfaces, solid–liquid boundaries, electrochemical cells, and nanoconfined geometries, these methods have shown that water is not merely a dielectric background or a host medium, but an active participant in various processes, like screening, ion specificity, proton transport, charge separation, and vibrational energy flow. The preceding sections make clear that no single conceptual or theoretical framework unifies dilute electrolytes, structured electric double layers, reactive interfaces, and Ångström-scale confinement. The central task for the coming years is not only to refine spectroscopic observables but also to translate them into predictive molecular rules for aqueous interfacial and confined chemistry.

### 6.1 Concepts

#### 6.1.1 Transferability of Bulk Benchmarks

A first conceptual issue concerns the transferability of bulk benchmarks to heterogeneous environments. Bulk water and simple electrolyte solutions provide important spectroscopic reference points. They were extensively investigated to extract information on frequency maps, linewidths, spectral diffusion timescales, vibrational lifetimes, and mode-coupling patterns.^27^ Yet, the usefulness of such benchmarks is necessarily conditional. Once translational symmetry is broken, electric fields become large and spatially varying, ion concentrations approach the water-in-salt regime, or confinement suppresses the emergence of a bulk-like interior, bulk-derived spectral–structural correlations can fail in subtle but important ways. A central conceptual challenge is therefore to determine not simply whether bulk benchmarks apply, but under what conditions, over which length scales, and for which observables they remain predictive.

Hydrogen-bond dynamics provide an instructive case study. For the OH stretch of dilute HOD in D_2_O, the frequency–frequency correlation function exhibits sub-200-fs local dynamics, including an oscillatory feature, and a ∼ 1.4 ps relaxation associated with collective reorganization of the hydrogen-bond network.^145^ At seemingly different interfaces, like the air–water interface^204^ or the phosphate–water interface of DNA,^292^ fast fluctuation components can occur on comparable timescales despite broken symmetry. This does not imply identical correlation functions: phosphate probes can also report much slower components, and different vibrational reporters weight different motions. This partial similarity suggests that the fastest component (∼100–200 fs) is governed by short-range forces, e.g., from librational motions, weakly perturbed by boundary conditions, while the slower component (∼1–3 ps) reflects hydrogen-bond exchange where reduced coordination may be partly offset by orientational constraints and connectivity to the surrounding network. Deviations emerge when interfacial perturbations alter either energetic barriers (strong electrostatic fields at charged interfaces, multivalent ion coordination) or configurational pathways (geometric frustration in rigid nanopores^344,539^ ). Understanding when bulk dynamics transfer therefore requires identifying how local hydrogen-bond topology, electrostatic perturbations, and geometric constraints combine to determine the effective energy landscape.

#### 6.1.2 Beyond the Hydrophobic/Hydrophilic Dichotomy

A second issue is whether hydrophobic versus hydrophilic hydration can serve as a unifying vocabulary across diverse interfaces. We believe that this distinction still remains powerful because it organizes many recurring spectroscopic motifs: dangling or weakly hydrogen-bonded OH groups, interfacial orientation, spectral diffusion, and the degree to which hydrogen bond rearrangements resemble or depart from bulk behavior. However, this review also shows that the binary picture of hydrophobicity vs hydrophilicity is incomplete. Macroscopically similar interfaces can differ microscopically because of chemical specificity, local roughness, polarizability, defects, charge regulation, and dimensionality.^265,394,397^ Conversely, spectrally similar interfaces can conceal different ion distributions or different microscopic hydrogen bond topologies.

Systematic cross-interface comparisons reveal both the utility and limitations of the hydrophobic/hydrophilic classification. The air–water interface, characterized by a narrow dangling OH peak near 3700 cm^–1^, serves as the canonical hydrophobic reference and is nearly replicated at suspended graphene–water interfaces, confirming minimal perturbation by atomically thin, weakly interacting sheets. However, oil–water interfaces show enhanced hydrogen-bonded intensity relative to air–water, suggesting stronger orientational ordering at condensed-phase boundaries—a distinction not captured by binary hydrophobicity.^229^ More strikingly, water in hydrophobic nanopores exhibits reduced tetrahedral coordination and slowed dynamics despite sharing the “hydrophobic” label.^546^ Similarly, ion-specific effects vary dramatically: at air–water, most salts produce subsurface enrichment with minimal free-OH perturbation;^247^ at graphene–water, strong NaCl accumulation causes only minor spectral changes; while at charged lipid interfaces, ion binding generates strong fields and pronounced water reorientation.^592^ These variations underscore that interfacial behavior depends not on hydrophobicity alone, but on coupled descriptors including polarizability, rigidity, confinement length scale, and charge distribution. A more useful classification of hydrophobicity may therefore be multidimensional, built around a small set of coupled descriptors such as hydrogen-bond topology, electrostatic boundary conditions, interfacial rigidity, spatial extent of the perturbed region, and the degree of chemical specificity in ion or water binding. Developing such a framework remains an open challenge, but it would provide a more transferable language for comparing results across the diverse systems discussed in this review.

Toward this goal, quantitative benchmarks across interface types require consistent definitions. Reported fields at the neat air–water interface,^593^ charged DNA interfaces,^291^ and oil–water emulsions,^230^ refer to different local environments, projections, and averaging procedures. They should not be ranked as a single interfacial-field scale or equated with a macroscopic double-layer field. Nanoconfinement introduces additional complexity through anisotropic dielectric response, modified image-charge effects, and suppression of diffuse-layer formation. Fast and slow spectral-diffusion components recur across several bulk and interfacial systems, but their values depend on the reporter, isotope composition, and analysis,^27,203,260,594^ whereas highly concentrated water-in-salt electrolytes can display a dominant ∼ 0.5 ps component^158^ a limiting case conceptually related to extreme nanoconfinement but not yet systematically compared. Establishing when each descriptor (field strength, confinement length, charge density, rigidity) dominates observed behavior would transform qualitative interface categories into predictive frameworks.

#### 6.1.3 The Double Layer as a Fluctuating Molecular Object

A third conceptual development concerns the EDL. The classical language of Stern and diffuse layers remains useful, but the molecular picture emerging from ultrafast nonlinear spectroscopy is richer and less separable than the mean-field picture suggests.^15^ It is worth being explicit about where continuum theory fails, because three of its tacit assumptions break down in measurable ways. First, the dielectric response need not be uniform: an out-of-plane dielectric constant of approximately 2 has been reported for molecularly thin confined water,^39^ but this confinement result should not be assigned universally to Stern-layer water or attributed solely to orientational saturation. Saturation of charge-induced water alignment provides a separate constraint on interfacial response.^592^ Second, ions are not point charges in a uniform medium, but objects that retain, partially shed, or share hydration shells, and HD-VSFG decomposition at charged oxide and lipid interfaces resolves Stern-layer and diffuse-layer subensembles with distinct hydrogen-bond strengths.^439,444,445^ Recent 2D HD-VSFG measurements sharpen this: spectral diffusion assigned to the diffuse layer is bulk-like at a cationic lipid interface (∼0.1 ps) but roughly an order of magnitude slower at an anionic one.^595^ The microscopic origin and spatial range of this difference remain unresolved. Lipid headgroup chemistry changes together with charge sign, and simulations show that the apparent range of an interfacial perturbation depends on the observable.^594^ The salt-dependent spectroscopic component is weighted by the χ^(3)^ response and optical interference, rather than being a spatially resolved measurement throughout a Debye-length-thick layer,^264^ and the slow time constant carries large uncertainty. These measurements therefore do not by themselves establish that reversing the field changes water dynamics several nanometers from the surface. Disentangling charge sign, headgroup chemistry, and depth weighting is a concrete experimental and theoretical challenge. Third, and most consequentially, the double layer is not static on the relevant timescales: hydrogen-bond fluctuations proceed on 0.1–1 ps,^595,596^ while the ionic atmosphere reorganizes on picoseconds to tens of picoseconds, rate-limited by ion conduction rather than water reorientation.^249^ A static potential profile alone does not specify the variance or correlation time of the field at a reacting species; whether an averaged description suffices depends on the coupling and time scale separation. Water orientation, hydrogen-bond connectivity, interfacial fluctuations, finite ion size, specific adsorption, and subsurface ion stratification can all affect the measured response. Therefore, the EDL should not be viewed only as a static charge-compensation profile, but as a fluctuating molecular object in which water, ions, and surface functionalities are dynamically coupled.

The practical consequence is a reinterpretation of the routinely extracted quantities. Stern-layer capacitance, conventionally a fitting parameter with a geometric interpretation, acquires a partly molecular one, reflecting the orientational compliance of a small water population whose response saturates, superimposed on ion-specific adsorption; the effective dielectric constant is a coarse-grained average over a strongly nonmonotonic profile. Field distributions and their correlation times may help explain rates that are not captured by mean-field analysis. Part of this is not new: within linear response, the reorganization energy is fixed by the mean-square fluctuation of the reaction energy gap scaled by the thermal energy. Relating this energy gap variance to a spectroscopically measured field variance additionally requires a calibrated coupling to the reaction coordinate, a compatible local environment, and an appropriate linear-response model. The testable content is quantitative: whether the field variance measured spectroscopically, converted through the reaction’s charge displacement, reproduces the reorganization energy obtained electrochemically for the same reaction on the same surface. Disagreement could reflect non-Gaussian statistics, different probe and reactant environments, an incomplete coupling model, or other contributions to reorganization.^597^ A complementary test concerns kinetic dispersion: slow environmental exchange relative to the reaction waiting time can produce heterogeneous kinetics, whereas rapid exchange can support an averaged rate description. Single-exponential kinetics are expected only under additional assumptions such as a single dominant reaction pathway. Locating that crossover would give more direct access to the coupling than any correlation with the mean-field. These comparisons are proposed tests rather than established consequences; relevant ingredients already exist: Stark probes measure interfacial fields under potential control,^598^ the mean-field/barrier correlation is established in enzymes,^599^ field inhomogeneity at electrodes is identified as a failure mode of mean-field treatment,^597^ and interfacial water reorganization already serves as a kinetic descriptor in hydrogen electrocatalysis,^600^ using the mean structure rather than its distribution.

Beyond model membranes, this breakdown is most severe in contemporary electrochemical energy systems. Concentrated battery electrolytes, redox-flow cells, and ionic–liquid interfaces contain polyatomic ions whose dimensions match or exceed those of the solvent, so steric repulsion, site-specific ion–solvent interactions, and solvation-shell rearrangement rather than the mean potential govern interfacial structure and charge transport, connecting to the water-in-salt limit noted above. The theory here is mature: modified Poisson–Boltzmann treatments with finite ion size^601^ and correlation-corrected continuum models predict overscreening at low bias, giving way to counterion crowding at high bias.^602^ What is missing is the molecular-level constraint on it, since these models are parametrized by an effective ion size and correlation length that interface-specific vibrational spectroscopy is among the few probes able to test.^603^ One of the most important conceptual questions is how the microscopic structure of this interfacial region controls macroscopic observables such as capacitance, charge-transfer kinetics, selectivity, interfacial friction, and chemical stability.

#### 6.1.4 Physical Reorganization versus Chemical Change

A fourth issue is the distinction between physical and chemical reactivity at interfaces.^265,407,411,462^ This distinction is easy to state but is often difficult to apply to a real situation. Field-induced reorientation, wetting transitions, phase changes, conformational rearrangements, or local dielectric reorganization can substantially alter surface-specific vibrational spectra and even reaction outcomes without involving bond making or breaking. On the other hand, proton transfer, chemical adsorption, ligand exchange, redox chemistry, and catalytic turnover are chemical processes even though they are sometimes preceded or accompanied by physical reorganization or transformation. Ultrafast nonlinear vibrational spectroscopy offers a promising approach to separate these regimes because it can simultaneously monitor spectral diffusion, chemical exchange, vibrational population dynamics, and the emergence of new resonant species at different frequencies. The broader conceptual opportunity is to use time-domain observables not just to describe interfacial change but also to determine whether an interface is merely reorganizing or genuinely changing the mechanism.

#### 6.1.5 Nanoconfinement and the Limits of the Term “Aqueous Phase”

Finally, nanoconfinement forces a conceptual reassessment of what is meant by an aqueous phase in general. In sufficiently narrow, strongly confined geometries, the distinction between interfacial and core water becomes vague, because electric double layers can spatially overlap, dielectric screening becomes nonlocal and anisotropic, and proton activity or ion transport can no longer be interpreted as perturbations of bulk liquid behavior.^344,539,542^ Consequently, one of the major goals would be to move beyond a case-by-case, exemplary study of unusual confined-water phenomena and toward a more general picture that is useful for relating confinement geometry, chemical boundary conditions, and hydration topology.

### 6.2 Theory and Computational Studies

Theoretical progress begins with a simple recognition that the frameworks useful for describing dilute bulk electrolytes are no longer sufficient for concentrated, interfacial, reactive, and confined aqueous systems. Traditional approaches such as Debye–Hückel theory, Poisson–Boltzmann treatments, and Gouy–Chapman–Stern concepts may remain valuable starting points because they clarify the effects of screening, charge compensation, and the mean-field electrostatic effects. In their simplest forms, however, these approaches do not capture finite ion size, ion polarizability, hydration-shell chemistry, nonlocal dielectric response, strong ion–ion correlations, ion network formation in high-salt electrolytes, or Ångström-scale heterogeneity that becomes decisive at interfaces and under confinement. What is ultimately needed is an encompassing liquid-state theory of aqueous heterogeneity that remains valid across a continuous range from dilute bulk electrolytes to water-in-salt networks, structured electric double layers, and confined electrolytes with no true bulk region.

Atomistic simulations have already become indispensable in the study of interfacial water structure and dynamics. However, their future role should be more ambitious than just the spectral interpretation of experiments. The most useful models will be those that reproduce not only equilibrium structure but also the observables that nonlinear vibrational spectroscopy measures directly, such as line shapes, phase-sensitive response, spectral diffusion, vibrational lifetimes, population transfer, photothermal effects, multipoint frequency correlation functions, and truly multidimensional vibrational/electronic spectra. The current state of agreement between simulations and experiments reveals both remarkable progress and persistent challenges. For bulk water, polarizable force fields such as AMOEBA^604^ and MB-pol,^100^ combined with machine-learning potentials trained on high-level electronic structure data,^105–110^ now achieve near-quantitative reproduction of linear IR spectra and successfully predict 2D-IR line shapes, frequency–frequency correlation functions,^99,100^ and even subtle nuclear quantum effects.^118,119^ This success reflects decades of iterative refinement and extensive experimental benchmarks from isotopically diluted systems that minimize the intermolecular coupling complications.

Despite such impressive success, certain challenges remain even in the description of bulk water and dilute electrolytes, such as the description of strong H-bonding configurations and associated enhancements of transition dipoles. A complete description of the proton continuum and its time-dependent nonlinear response in a fully fluctuating bulk aqueous environment remains challenging,^144^ and the influence of ions on proton hydration geometries is largely unaddressed. Overcoming the nonreactive nature of standard classical models to describe transient bond formation or breaking, together with the description of the strong anharmonic couplings underlying Fermi resonances remains a critical bottleneck.^113,114^

At interfaces, the picture is even more heterogeneous. Air–water and suspended graphene–water interfaces show excellent agreement between ab initio MD and phase-resolved SFG measurements, correctly reproducing spectral sign, relative intensities, and isotope-dependent orientational preferences.^84^ In contrast, oil–water interfaces present unresolved discrepancies:^227,229^ different experimental studies report conflicting H-bond strengths depending on the organic phase, sample preparation, and measurement geometry, while simulations show sensitivity to force-field parametrization and system-size effects. Whether enhanced SFG intensity at oil–water relative to air–water reflects stronger hydrogen bonding, altered orientation, or increased roughness remains unclear, in part because competing experimental results leave structural models underconstrained. It becomes clear that polarizable force fields, many-body descriptions of water, and machine-learning potentials have dramatically improved our understanding of ultrafast hydrogen-bond fluctuations and interfacial structure via accurate simulations. The next challenge is, then, to extend the application to systems where reactivity, extreme fields, heterogeneous surfaces, and compositionally complex electrolytes all matter simultaneously *in operando*.

Reactive processes pose an especially demanding challenge. Proton transport involving rapid bond breaking and forming, hydroxide dynamics, hydrolysis, field-driven dissociation, charge transfer, and electrocatalytic intermediates all require models that can move beyond fixed bonding structures. At highly charged interfaces, accurate decomposition of χ^(2)^ and χ^(3)^ contributions requires proper treatment of long-range electrostatics, depth-dependent dielectric response, and field-induced polarization—features that challenge classical force fields and continuum approximations alike. High-level anharmonic and quantum-dynamical calculations have provided detailed assignments of hydrated-proton spectra.^605^ Extending these descriptions to the time-dependent nonlinear response in a fully fluctuating aqueous environment, particularly at interfaces and under confinement, remains a demanding challenge.^606,607^ Machine-learning potentials offer promise by incorporating electronic polarization, charge transfer, and bond breaking at reduced computational cost, but their transferability to interfaces—where training data are sparse and electronic structure may differ qualitatively from bulk—has not been systematically validated. At present, there is still a significant gap between the systems we can spectroscopically interrogate and those we can predict with full molecular detail. The future likely lies in hybrid strategies: reactive or machine-learned potentials trained on high-level electronic-structure data, embedded approaches for charged or metallic interfaces, and multiscale descriptions that connect local chemistry to longer-range electrostatics and transport.

A parallel theoretical development should be the direct simulation of nonlinear responses themselves. In many cases, interfacial structure is still inferred by comparing measured spectra with structural motifs extracted from molecular dynamics trajectories. While that strategy has been enormously productive, the field is moving toward a more stringent standard: explicit calculation of linear and nonlinear response functions, including phase, tensor character, interference effects, and spectroscopic depth sensitivity. This requires validation protocols that extend beyond static spectra to include dynamical observables—spectral diffusion timescales, vibrational lifetimes, reorientation dynamics, and chemical exchange rates—recognizing that models may reproduce linear IR while failing to capture 2D-IR cross-peaks or SFG phase. Uncertainty quantification through ensemble averaging, force field comparison, and sensitivity analysis should become standard practice, enabling distinction between robust predictions and method-dependent artifacts. For interfacial and multidimensional spectroscopy in particular, this will require algorithms that can efficiently compute nonlinear optical response functions for large, heterogeneous, and often nonequilibrium systems. Such calculations should treat realistic experimental observables rather than idealized local spectra or indirect measures.

Bridging length and time scales remains the overarching theoretical problem. The relevant physics spans local H-bond geometry, picosecond spectral diffusion, nanometer-scale ion stratification, slow charge relaxation, and mesoscale confinement or porous architectures. No single model can capture all of this at the same level of fidelity and accuracy. The most promising route is therefore a hierarchy of mutually constrained descriptions, in which atomistic simulation informs coarse-grained or continuum models, while ultrafast spectroscopic observables serve as direct benchmarks and constraints at the molecular level. Closer coupling between experimentalists and theorists in designing and interpreting measurements will be essential for resolving ambiguities such as those at oil–water interfaces and for systematically documenting where current methods succeed and fail. In that sense, the long-term objective is an experiment–theory feedback loop in which spectroscopy is used to refine molecular models, and those models in turn generate testable predictions for new classes of heterogeneous aqueous systems under experimental investigations.

### 6.3 Experimental Methods and Opportunities

Experimentally, the most immediate priority is to push interface-specific nonlinear vibrational spectroscopy beyond the small set of well-controlled systems. Air–water and a handful of model-charged interfaces have played an essential role in method development and benchmark measurements. However, the field will increasingly be judged by its ability to address buried interfaces, realistic electrochemical cells in operation, reactive catalytic boundaries, membranes in living cells, nanopores used in chemical applications, and heterogeneous solids under operando conditions. Achieving this will require improvements in sensitivity, stability, and quantitative interpretation, particularly for systems where weak resonant signals compete with or are largely hidden under strong nonresonant backgrounds,^375^ pump-and-probe scattering at heterogeneous interfaces,^608^ strong attenuation due to absorption, photothermal heating, or, at times, plasmonic distortions.^374^

Time resolution and spectral coverage must also expand in both directions.^521,522^ First, sub-100 fs measurements in surface-specific nonlinear vibrational spectroscopy remain crucial for resolving ultrafast inertial and wobbling motions, vibrational coherence transfer, ultrafast dephasing, and the earliest steps of field-driven or reactive structural change involving light atoms such as protons. Second, many of the most interesting interfacial processes, such as chemical exchange in electrolytes, EDL relaxation, adsorption/desorption, phase transitions, structural aging, phase coexistence and segregation, and confined transport, all extend well beyond the picosecond regime. Future platforms should therefore connect ultrafast structural observables to slower nonequilibrium evolution without sacrificing the interfacial selectivity. Equally important is a broader spectral range, from low-frequency intermolecular and THz modes to localized reporter vibrations in the mid-IR range and the OH/OD stretching and bending regions, because no single vibrational window captures the full hierarchy of aqueous structure and dynamics.

A particularly important direction is the development of novel, versatile multidimensional vibrational/electronic spectroscopy for selectively studying interfacial aqueous solutions. Conventional and heterodyne-detected VSFG have already demonstrated their power in defining interfacial structure, such as molecular orientation and H-bonding networks, while time-resolved SFG has clarified mode-specific relaxation dynamics, including population relaxations and vibrational dephasing of interfacial water and probe molecules. The next step is to make multidimensional and correlation-based methods more broadly usable and more routinely quantitative for investigating mode–mode coupling, electron-vibration coupling, and vibrational dynamics on electronically excited states. That includes not only 2D-VSFG and related interface-specific approaches (e.g., electronic sum-frequency generation) but also strategies that combine the intrinsic selectivity of SFG with the reporter specificity of 2D-IR. Buried interfaces with optically transparent substrates, electrified boundaries, and weakly scattering confined systems are likely to be especially revealing systems of research interest because they are precisely those in which static spectra alone are difficult to interpret and in which nonequilibrium chemical or physical processes occur.

Another major opportunity lies in combining nonlinear vibrational spectroscopy with those of complementary methods. Electrochemistry, X-ray scattering, electron microscopy, AFM, scanning probe methods, and structural scattering techniques each provide information that vibrational spectroscopy alone cannot provide, including morphology, elemental distributions, long-range ordering, nanoscopic resolution, electronic state changes, etc. The most informative experiments of the next decade will likely be those in which these approaches are synchronized rather than merely juxtaposed sequentially.^425^ For example, spectroscopic measurements of water orientation or intermediate lifetimes become far more informative when paired with an independent control of surface potential, pore geometry, local composition, or structural phase.

Experimental design will also benefit from a more deliberate use of intrinsic and extrinsic vibrational reporters. Water itself, e.g., OH stretch and bending modes, is a powerful reporter but not always a sufficient one. Reporter modes of molecular ions, carbonyl-containing intermediates, nitriles, various nitrile analogues, or selectively placed vibrational probes on target surfaces or at interfaces can be particularly useful for mechanistic interpretation, particularly at electrochemical and biological interfaces.^137^ In confinement, reporter-based approaches may become essential for separating interfacial from core-like domains or for connecting spectroscopic signatures to effective acidity, pH, ion pairing, ion clusters, a more extended ion network, or charge-carrier transport pathways.

Finally, the field will need increasingly rigorous standards for quantitative analyses. At charged and metallic interfaces, surface structure, depth sensitivity, interference between resonant and nonresonant responses, and the optical consequences of time-dependent refractive indices can all complicate interpretation. As ultrafast nonlinear vibrational spectroscopy is extended to more realistic environments, careful control experiments, benchmark systems, and transparent analysis protocols will become as important as raw instrumental developments. The ultimate experimental goal is not just to detect more complex interfaces but to do so in a way that enables cumulative, constructive comparisons across laboratories, materials classes, and theoretical models.

Although outside the strict scope of nonlinear vibrational spectroscopy, complementary nonvibrational probes may also become increasingly valuable in this broader methodological development. In particular, techniques sensitive to long-range orientational correlations or mesoscopic electrostatic organization may help connect local structural information from vibrational spectroscopy to larger-scale interfacial order.

### 6.4 Grand Challenges

First, can we build a predictive multiscale theory of aqueous heterogeneity that remains valid from dilute bulk electrolytes to concentrated interfacial and nanoconfined systems? At present, this field still relies on a kind of patchwork of bulk benchmarks, interface-specific phenomenology, and system-dependent simulations. What is missing is a predictive framework that links nonlinear vibrational observables directly to molecular structure, dynamics, and thermodynamics across environments where the relevant physics and chemical compositions change. Such a framework must treat water as an active component of the problem and remain accurate when H-bond networks are fragmented, electric fields are heterogeneous, ion correlations are strong, and no true bulk reservoir of water exists. Solving this problem would elevate the field from system-specific interpretation to transferable molecular design.

What is the true molecular and ultrafast nature of the EDL? This is one of the most frequently asked questions in this field. The EDL remains one of the most consequential and least completely resolved research topics in interfacial science. The central unanswered question is not only how ions arrange on average but also how water orientation, hydrogen-bond connectivity, ion specificity, Stern-layer structure, fluctuations, and nonequilibrium relaxation together define the interfacial electrostatic environment and ultimately determine reactivity. Resolving this problem will require going beyond static potential profiles toward a molecular-level time-dependent description of how the EDL forms, fluctuates, screens, and reorganizes during adsorption, charge transfer, catalysis, and transport. A true molecular theory of the EDL would have consequences far beyond spectroscopy, directly informing electrocatalysis, membrane science, corrosion, energy storage, and interfacial self-assembly.

Similarly, how do confinement and interfacial heterogeneity reshape acidity, ion transport, proton transport, and chemical reactivity? When does an interface merely reorganize a reaction environment or fundamentally change the reaction mechanism? This is probably the broadest and most interdisciplinary of the challenges. Under nanoconfinement and in compositionally heterogeneous structures, water should not be treated as a small-scale bulk liquid. Due to boundaries, dielectric screening becomes geometry-dependent; acidity/basicity may shift; boundary layers, such as EDLs, overlap; and transport pathways become correlated with the local confined structure. Unfortunately, this research field lacks general rules for addressing questions such as when these changes remain quantitative and when they become qualitative. Equally unresolved is the distinction between interfaces that accelerate or suppress reactions by reorganizing solvation, concentration, and orientation and interfaces that create new mechanistic pathways. Ultrafast nonlinear vibrational spectroscopy is, in this regard, uniquely positioned to address some of these questions because it can probe both the local structural ensemble and, more importantly, its temporal evolution. The grand challenge is to turn those capabilities into a predictive framework for confined and interfacial aqueous chemistries.

In summary, the long-term promise of ultrafast nonlinear vibrational spectroscopy is that it can reveal how water and ions behave at complex boundaries and provide the molecular language needed to connect interfacial structure, fluctuations, transport, and reactivity across a wide range of aqueous systems. The field is transitioning from establishing individual case studies to constructing a general science of aqueous heterogeneity. Achieving this objective requires advancing concept formation, theoretical development, and experimental innovation.
